# Comparative effectiveness of regional analgesia techniques after gastrectomy for gastric cancer: a systematic review and network meta-analysis of randomized trials

**DOI:** 10.3389/fmed.2026.1829566

**Published:** 2026-06-15

**Authors:** Danling Hu, Weiwei Cai, Anwen Zheng, Shuaili You, Shan Zhong

**Affiliations:** Department of Anesthesiology, Children's Hospital of Nanjing Medical University, Nanjing, China

**Keywords:** gastric cancer, immune function, network meta-analysis, postoperative pain, radical gastrectomy, regional analgesia

## Abstract

**Objective:**

To compare the analgesic efficacy and immunomodulatory effects of regional analgesia techniques after radical gastrectomy.

**Methods:**

This network meta-analysis followed PRISMA-NMA guidelines. PubMed, EMBASE, Cochrane Library, and Web of Science were searched from inception to December 2025. Randomized controlled trials comparing epidural analgesia (EA) or other regional techniques with systemic analgesia (SA) were included. Primary outcomes were short- and long-term postoperative pain intensity assessed by the visual analog scale (VAS). Secondary outcomes included postoperative opioid consumption and CD4^+^/CD8^+^ T-cell ratio. Effects were estimated as mean differences (MDs) or standardized mean differences (SMDs) using a random-effects consistency model. Ranking probabilities were assessed using the surface under the cumulative ranking curve (SUCRA). Risk of bias and certainty of evidence were evaluated using RoB 2.0 and CINeMA–GRADE.

**Results:**

Twenty-three trials involving 1,700 patients and 11 analgesic strategies were included. Compared with SA, several regional techniques reduced postoperative pain. Intertransverse process plane block (ITPB; MD = −2.28; 95% CI −3.63 to −0.94) significantly improved long-term pain at rest. ITPB also reduced long-term pain during movement (MD = −1.86; 95% CI −2.81 to −0.90). EA reduced postoperative opioid consumption (SMD = −2.25; 95% CI −3.52 to −0.99) and increased CD4^+^/CD8^+^ ratio (MD = 0.18; 95% CI 0.03 to 0.34). Evidence certainty ranged from low to moderate.

**Conclusion:**

Regional analgesia may provide beneficial effects in reducing postoperative pain and opioid consumption after gastrectomy for gastric cancer. Intertransverse process plane block (ITPB) showed favorable ranking probabilities across several postoperative pain outcomes, although these findings should be interpreted cautiously given the limited certainty of evidence. Similarly, the potential immunomodulatory effects of transcutaneous electrical acupoint stimulation combined with transversus abdominis plane block (TEAS–TAP) require further confirmation. However, given the low- to moderate-certainty evidence for several comparisons, these findings should be interpreted cautiously. Further high-quality randomized controlled trials are needed to confirm these results.

## 1 Background

Gastric cancer remains one of the most prevalent malignancies in China, ranking among the leading causes of cancer-related morbidity and mortality (1–3). Owing to its high risk of recurrence and metastasis, surgical resection remains the primary curative treatment (4). In recent years, minimally invasive approaches, particularly laparoscopic radical gastrectomy, have been increasingly adopted. High-quality randomized clinical trials have demonstrated that laparoscopic gastrectomy provides oncologic outcomes comparable to those of open surgery, while offering perioperative advantages such as reduced blood loss and accelerated recovery (5). Despite these benefits, postoperative pain remains a clinically relevant concern even after laparoscopic procedures. A substantial proportion of patients experience moderate to severe incisional and visceral pain during the early postoperative period (6). Inadequately controlled pain may trigger sympathetic activation, contributing to adverse physiologic responses such as gastrointestinal dysmotility, delayed gastric emptying, and postoperative ileus. Furthermore, insufficient analgesia can impair effective coughing and deep breathing, thereby increasing the risk of pulmonary complications, including atelectasis, infection, and hypoxemia (7). Effective pain control is therefore a central component of enhanced recovery after surgery (ERAS) pathways, as it not only improves patient comfort but also facilitates early mobilization, restoration of gastrointestinal function, and overall postoperative recovery. Current guidelines advocate multimodal analgesia strategies that combine systemic non-opioid analgesics with regional analgesia techniques, while limiting opioid use to the lowest effective dose. This approach aims to provide adequate nociceptive control while minimizing opioid-related adverse effects, including respiratory depression, postoperative ileus, nausea, and vomiting (8, 9). However, local anesthetic infiltration alone often provides insufficient analgesia for major abdominal procedures. Although systemic opioids remain effective, their use is associated with dose-dependent adverse effects that may delay recovery and increase postoperative morbidity (10, 11). Against this background, regional analgesia techniques have emerged as an important component of perioperative pain management for abdominal surgery. By targeting afferent nociceptive pathways, these techniques may improve analgesic efficacy, reduce postoperative opioid consumption, and mitigate opioid-related complications. Nevertheless, the comparative effectiveness of different regional analgesia strategies in patients undergoing radical gastrectomy remains incompletely defined. A clearer understanding of their relative benefits is essential to optimize analgesic protocols and improve postoperative outcomes in this population.

Postoperative pain following laparoscopic radical gastrectomy primarily originates from the anterior branches of the thoracoabdominal intercostal nerves (T6–T10), which provide sensory innervation to the upper abdominal wall. Targeted blockade of these nerves has therefore emerged as a rational and effective strategy for postoperative analgesia. Although general anesthesia combined with thoracic epidural analgesia has traditionally been regarded as the gold standard for major abdominal surgery, epidural techniques are associated with well-recognized limitations, including hypotension, urinary retention, and potential neuraxial complications, which may restrict their use in certain patients (12). The introduction and widespread adoption of ultrasound guidance have substantially expanded the role of fascial plane blocks in perioperative pain management. Techniques such as transversus abdominis plane (TAP) block, erector spinae plane block (EspB), and other interfascial approaches are increasingly incorporated into multimodal analgesia protocols. Accumulating evidence suggests that these regional analgesia techniques can effectively reduce postoperative pain intensity, decrease postoperative opioid consumption and opioid-related adverse effects, and facilitate recovery of gastrointestinal function and overall postoperative recovery (13–15).

Because direct head-to-head randomized controlled trials comparing different regional analgesia techniques are scarce, the optimal strategy for postoperative analgesia following laparoscopic radical gastrectomy remains uncertain. Network meta-analysis provides a robust methodological framework that enables both direct and indirect comparisons across multiple interventions within a unified analytical model. Therefore, we conducted a systematic review and network meta-analysis to compare the analgesic efficacy and immunologic effects of available regional analgesia techniques and to provide evidence-based guidance for optimizing postoperative analgesic management in patients undergoing laparoscopic radical gastrectomy.

## 2 Materials and methods

This network meta-analysis was conducted in accordance with the Preferred Reporting Items for Systematic Reviews and Meta-Analyses extension statement for network meta-analyses (PRISMA-NMA; Supplementary Table S1) (16). To enhance transparency and methodological rigor, the study protocol was prospectively registered in the International Prospective Register of Systematic Reviews (PROSPERO; CRD420261299856) before data extraction and analysis.

### 2.1 Data sources and search strategy

A comprehensive literature search was performed in PubMed, EMBASE, the Cochrane Library, and Web of Science from database inception to 21 December 2025. The search strategy incorporated both controlled vocabulary terms (e.g., MeSH and Emtree) and free-text keywords related to gastric cancer, gastrectomy, regional anesthesia and analgesia, and randomized controlled trials. The full electronic search strategies for each database are provided in Supplementary Table S2. No language restrictions were applied.

### 2.2 Selection criteria

#### 2.2.1 Inclusion criteria

(1) Randomized controlled trials involving patients with a confirmed diagnosis of gastric cancer who were scheduled to undergo radical gastrectomy.
(2) Randomized controlled trials evaluating epidural analgesia or ultrasound-guided regional nerve block techniques—including transversus abdominis plane block (TAP), rectus sheath block (RSB), quadratus lumborum block (QLB), intertransverse process plane block (ITPB), erector spinae plane block (EspB), serratus anterior plane block (SAPB), paravertebral block (PVB), or external oblique intercostal block (EOI) —were eligible for inclusion.
(3) Randomized controlled trials comparing regional analgesia techniques with systemic analgesia alone were also included. Systemic analgesia was defined as intravenous patient-controlled analgesia or intravenous and/or oral opioid administration without the use of regional analgesia.
(4) Randomized controlled trials reporting at least one of the following predefined outcomes were eligible for inclusion:
  (a) Postoperative pain intensity was assessed using the visual analog scale (VAS) at rest and/or during movement. Movement-related pain was defined according to the criteria used in each original study (e.g., coughing, turning in bed, or ambulation). The specific definitions of movement-related pain across individual trials were extracted and are summarized in Supplementary Table S3.
  (b) Immunologic function was evaluated using the CD4^+^/CD8^+^ T-cell ratio, defined as the proportion of peripheral blood CD4^+^ helper T lymphocytes to CD8^+^ cytotoxic T lymphocytes. This parameter was used as an indicator of cellular immune status and immune balance.
  (c) Postoperative opioid consumption was defined as the cumulative amount of opioids administered after surgery, including agents such as morphine and fentanyl.

#### 2.2.2 Timing of outcome assessment

Short-term postoperative pain was defined as pain intensity assessed using the visual analog scale (VAS) within 0–2 h after surgery. When multiple measurements were reported within this time window in a single study, the value closest to 2 h postoperatively was preferentially extracted to ensure consistency across studies.

Long-term postoperative pain was defined as pain intensity assessed using the VAS between 24 and 48 h after surgery. When both 24-h and 48-h measurements were reported, the 24-h value was preferentially extracted, as this time point is more consistently reported across studies and is commonly used in postoperative pain assessment. If only one measurement within this interval was available, that value was used. Studies that did not report VAS data within the predefined 24–48-h window were excluded from the pooled analysis of long-term postoperative pain.

#### 2.2.3 Exclusion criteria

Studies were excluded if they met any of the following criteria:

(1) Duplicate publications or multiple reports derived from the same patient cohort, in which case only the most complete or most recent report was included.
(2) Randomized controlled trials with unclear, incompletely defined, or non-extractable outcome data.
(3) Non-original studies, including review articles, case reports, editorials, and conference abstracts without sufficient data.

Titles and abstracts of all identified records were initially screened for eligibility. Full-text articles of potentially relevant studies were subsequently assessed to confirm inclusion. To minimize duplication, all included randomized controlled trials were independently reviewed by two investigators, and when multiple publications from the same study population were identified, the most complete and most recent report was selected for inclusion.

### 2.3 Data extraction and quality assessment

Two investigators independently extracted data from all eligible randomized controlled trials using a standardized data extraction form in accordance with PRISMA recommendations. Any discrepancies were resolved through discussion, and when necessary, a third investigator was consulted to reach consensus. The following data were extracted from each study: first author, year of publication, sample size, patient demographics (including age and sex), geographic location, follow-up duration, details of the intervention and control protocols, timing of regional anesthesia administration, regional anesthesia techniques and regimens, postoperative analgesic strategies, use of prophylaxis for postoperative nausea and vomiting (PONV), and administration of adjuvant medications. For continuous outcomes, mean values and corresponding standard deviations (SDs) were extracted. When SDs were not directly reported, they were calculated from available data using established statistical conversion methods. To further improve transparency and reproducibility, the extracted outcome data and corresponding statistical calculation information were provided in the Supplementary Tables S4–S9.

The methodological quality of included studies was independently assessed by two investigators using the Cochrane Risk of Bias tool version 2.0 (RoB 2.0). This validated instrument evaluates the risk of bias across five domains: bias arising from the randomization process, bias due to deviations from intended interventions, bias due to missing outcome data, bias in the measurement of outcomes, and bias in the selection of the reported results. Each domain was rated as low risk of bias, some concerns, or high risk of bias, in accordance with the RoB 2.0 guidance (17). Disagreements between reviewers were resolved through discussion or consultation with a third investigator.

### 2.4 Statistical analysis

All network meta-analyses were performed using Stata version 17.0 MP (StataCorp, College Station, TX, USA). For continuous outcomes measured using the same scale, such as postoperative pain intensity assessed by the VAS, treatment effects were estimated as mean differences (MDs) with corresponding 95% confidence intervals (CIs). For outcomes reported using heterogeneous measurement scales or different units, particularly cumulative postoperative opioid consumption involving different opioid agents and dosing units across studies, standardized mean differences (SMDs) with 95% CIs were used to enable comparison across studies. For multi-arm trials, pairwise comparisons were generated using the *augment* method while preserving the within-study correlation structure to avoid underestimation of standard errors arising from shared comparator groups. The primary analyses were conducted under the consistency assumption using a random-effects model, with between-study variance (τ^2^) estimated using the restricted maximum likelihood (REML) method. When closed loops were present within the treatment network, global inconsistency was evaluated using the design-by-treatment interaction model. Local inconsistency was further assessed using the node-splitting approach, with a two-sided *P* value < 0.05 considered indicative of statistically significant inconsistency. Loop-specific inconsistency was quantified using inconsistency factors (IFs), with 95% CIs including zero interpreted as indicating no significant disagreement between direct and indirect evidence. Network geometry was visualized using network plots, in which node size was proportional to the total number of participants assigned to each intervention, and edge thickness reflected the number of studies contributing direct comparisons. Treatment rankings were evaluated using the surface under the cumulative ranking curve (SUCRA), the probability of being the best treatment (PrBest), and mean rank values, providing complementary measures of relative treatment performance. Publication bias and small-study effects were examined using comparison-adjusted funnel plots when at least 10 studies were available for a given outcome. Sensitivity analyses were conducted using a leave-one-out approach, in which each study was sequentially excluded and the random-effects consistency model was refitted to assess the stability of the pooled estimates. In addition, sensitivity analyses and network meta-regression analyses were used to further explore potential sources of heterogeneity and inconsistency within the treatment networks. Finally, univariable network meta-regression analyses were performed to explore the potential influence of study-level covariates on treatment effects. Regression coefficients with corresponding 95% CIs and Wald test *P* values were reported, with *P* < 0.05 considered to indicate statistically significant effect modification.

### 2.5 Certainty of evidence assessment (GRADE)

The certainty of evidence for network meta-analysis estimates was evaluated using the Grading of Recommendations Assessment, Development and Evaluation (GRADE) framework in conjunction with the Confidence in Network Meta-Analysis (CINeMA) approach. Because all included studies were randomized controlled trials, the certainty of evidence was initially rated as high. Evidence certainty was assessed across six domains: within-study bias, indirectness, imprecision, heterogeneity, incoherence, and across-study bias (including publication bias and small-study effects). Within-study bias was evaluated using the Cochrane Risk of Bias tool version 2.0 (RoB 2.0), and these assessments were incorporated into the CINeMA contribution matrix to reflect the relative influence of each study on the corresponding network estimates. Indirectness was assessed by examining the assumptions of transitivity and exchangeability, with prespecified potential effect modifiers—including baseline clinical characteristics, intervention characteristics, and follow-up duration—considered to evaluate the comparability of populations, interventions, comparators, and outcome definitions across studies. Imprecision was evaluated using prespecified minimal clinically important difference (MID). For continuous outcomes, thresholds were defined as SMD = 0.5, MD = 1.0 for VAS pain intensity, and MD = 0.2 for the CD4^+^/CD8^+^ T-cell ratio (18, 19). Judgments were based on whether 95% CIs crossed the null value and/or the corresponding MID thresholds. Heterogeneity was assessed using the estimated between-study variance (τ^2^) from random-effects models, along with consideration of prediction intervals relative to the predefined MID thresholds. Incoherence was evaluated using CINeMA's assessment of agreement between direct and indirect evidence, based on node-splitting and design-by-treatment interaction models. Across-study bias was assessed by considering the comprehensiveness of the literature search, trial registration status, and the presence of small-study effects, as evaluated using comparison-adjusted funnel plots when applicable. Each domain was rated as “no concerns,” “some concerns,” or “major concerns,” in accordance with CINeMA guidance. The overall certainty of evidence for each comparison was then graded as high, moderate, low, or very low, following the GRADE framework.

## 3 Result

### 3.1 Characteristics of included trials

**Figure 1:**
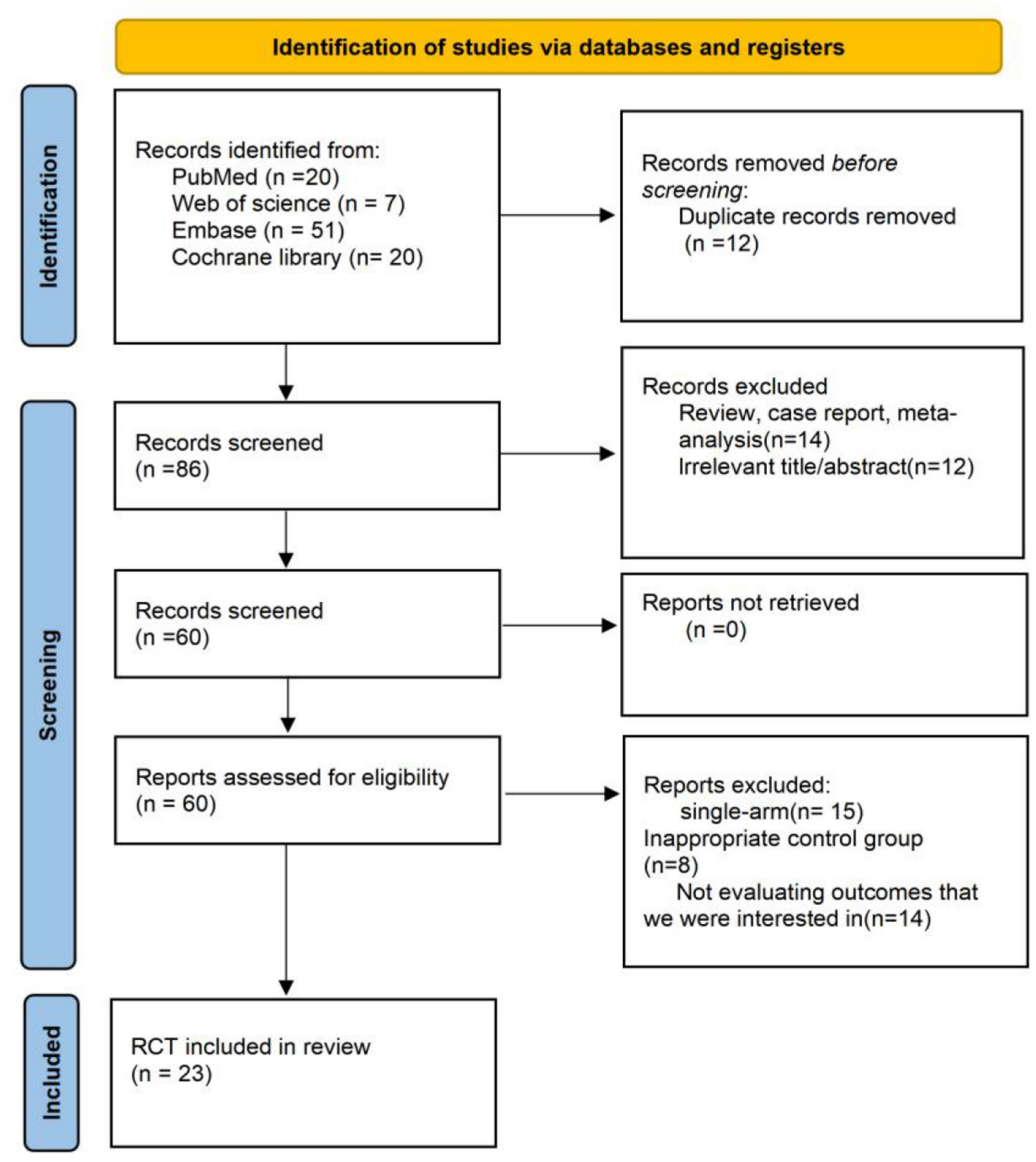
PRISMA 2020 flow diagram of study selection.

The initial database search identified 98 records. After removal of duplicates, 86 unique records remained for screening. Following title and abstract screening, 60 articles were deemed potentially eligible and underwent full-text review. Ultimately, 23 randomized controlled trials met the predefined inclusion criteria and were included in the network meta-analysis (Figure 1). Across the included trials, a total of 1,700 patients were randomized to one of 11 analgesic strategies: systemic analgesia (SA), epidural analgesia (EA), transversus abdominis plane block (TAP), rectus sheath block (RSB), quadratus lumborum block (QLB), intertransverse process plane block (ITPB), erector spinae plane block (EspB), external oblique intercostal block (EOI), serratus anterior plane block (SAPB), paravertebral block (PVB), transcutaneous electrical acupoint stimulation combined with transversus abdominis plane block (TEAS–TAP).

The included trials were published between 1995 and 2025 and were conducted predominantly in East Asian countries, including China, Japan, and South Korea. All enrolled patients underwent radical gastrectomy for gastric cancer, performed using either open or laparoscopic approaches. Study populations primarily consisted of middle-aged and older adults, with several trials specifically enrolling elderly patients (≥65 years). Male participants comprised the majority of the study populations. The regional analgesia techniques evaluated included EA, PVB, EspB, QLB, TAP, RSB, SAPB, and EOI, as well as combination approaches. Some studies also incorporated neuromodulatory interventions, such as TEAS. In most studies, control groups received systemic analgesia alone or sham block procedures. Postoperative analgesia was commonly administered using intravenous patient-controlled analgesia (PCIA) as part of a multimodal analgesic regimen. In addition, variability was observed across studies with respect to surgical approach (open vs. laparoscopic gastrectomy), timing and regimen of regional analgesia, and perioperative analgesic strategies. Regional blocks were typically performed before induction of general anesthesia or prior to surgical incision, with ropivacaine being the most frequently used local anesthetic. Follow-up durations ranged from the immediate postoperative period to 7 days after surgery. The most frequently reported outcomes included postoperative pain intensity, opioid consumption, and immunologic parameters, particularly the CD4^+^/CD8^+^ T-cell ratio. Although heterogeneity was observed across studies in terms of surgical approach, regional analgesia technique, anesthetic protocols, and follow-up duration, all included trials employed randomized controlled designs. Overall, these studies provide a robust evidence base for evaluating the analgesic efficacy and immunologic effects of regional analgesia in patients undergoing gastrectomy for gastric cancer. Detailed study characteristics are presented in Table 1 and Supplementary Table S10.

**Table 1:** Baseline characteristics of randomized controlled trials included in the network meta-analysis.

No.	References	Surgical procedure	Sample size (*n*)	Age (years)	Sex (male/female)	Country	Study design	BMI (kg/m^2^)	Body weight (kg)	Follow-up duration
1	Li et al. (33)	Open gastrectomy	20	61.26	31/9	China	Single-center	48.52	NR	48 h postoperatively
			20	57.64				46.14		
2	Liu et al. (17)	Open gastrectomy	48	56.3	47/49	China	Single-center	NR	59	72 h postoperatively
			48	58.6				NR	58.2	
3	Liu et al. (29)	Open gastrectomy	30	58.95	44/17	China	Single-center	NR	67.33	48 h postoperatively
			31	60.2				NR	65.9	
4	Wang et al. (22)	Open gastrectomy	20	59.7	21/19	China	Single-center	NR	64.3	7 days postoperatively
			20	59.3				NR	66.5	
5	Hu (23)	Open gastrectomy	70	72.25	109/31	China	Single-center	NR	52.26	24 h postoperatively
			70	71.89				NR	51.95	
6	Wu et al. (24)	Open gastrectomy	27	60	57/25	China	Single-center	21.6	58	72 h postoperatively
			29	61				21.6	58	
			26	62				21.3	56	
7	Xin et al. (25)	Open gastrectomy	100	60.74	138/62	China	Single-center	NR	61.27	72 h postoperatively
			100	59.84				NR	62.74	
8	Yoon et al. (26)	Laparoscopic gastrectomy	53	62.5	71/35	South Korea	Single-center	24.6	NR	7 days postoperatively
			53	58.8				24.2	NR	
9	Zgâia et al. (27)	Open gastrectomy	50	60.76	65/35	Romania	Single-center	28.53	NR	48 h postoperatively
			50	64.8				28.73	NR	
10	Zhou et al. (28)	Laparoscopic gastrectomy	40	65.4	52/28	China	Single-center	NR	58.5	3 days postoperatively
			40	66.3				NR	58	
11	Zhu et al. (21)	Not specified	30	61.1	32/28	China	Single-center	NR	NR	4 days postoperatively
			30	59.6				NR	NR	
12	Kawasaki et al. (30)	Not specified	10	52.2	14/6	Japan	Single-center	23.5	59	4 days postoperatively
			10	53.8				23.1	56.8	
13	Kun et al. (31)	Open gastrectomy	35	64	43/28	China	Single-center	NR	70	24 h postoperatively
			36	62				NR	72	
14	Hong et al. (32)	Open gastrectomy	21	57.8	31/10	South Korea	Single-center	NR	65.9	72 h postoperatively
			20	60.2				NR	65.7	
15	Wang et al. (34)	Laparoscopic gastrectomy	27	57.3	42/11	China	Single-center	22.2	65.1	48 h postoperatively
			26	54				23.1	67.7	
16	Hashimoto et al. (35)	Open gastrectomy	12	54.3	10/11	Japan	Single-center	NR	51.8	2 h after skin incision
			9	61.3				NR	54.4	
17	Chen et al. (20)	Laparoscopic gastrectomy	31	65.7	43/19	China	Single-center	23.5	66.5	48 h postoperatively
			31	65.6				24.6	67.2	
18	Wang et al. (36)	Laparoscopic gastrectomy	34	68.85	54/48	China	Single-center	22.49	61.74	7 days postoperatively
			34	68.88				22.82	62.47	
			34	67.03				22.48	62.47	
19	Tang (37)	Laparoscopic gastrectomy	30	58.3	41/19	China	Single-center	21.18	NR	48 h postoperatively
			30	60.9				20.25	NR	
20	Cheng et al. (38)	Laparoscopic gastrectomy	30	55	29/31	China	Single-center	NR	65	48 h postoperatively
			30	53				NR	63	
21	Shang et al. (39)	Open gastrectomy	30	66	40/20	China	Single-center	24.6	NR	3 days postoperatively
			30	65.5				24.5	NR	
22	Xing et al. (41)	Laparoscopic gastrectomy	29	69.45	64/23	China	Single-center	23.05	NR	7 days postoperatively
			29	68.48				22.99	NR	
			29	70.41				22.71	NR	
23	Jeong et al. (40)	Open gastrectomy	28	52	37/21	South Korea	Single-center	24.5	69	24 h postoperatively
			30	55				23.8	65	
			80	51.21				22.23	NR	

NR, not reported; BMI, body mass index.

### 3.2 Risk of bias assessment (RoB 2.0)

**Figure 2:**
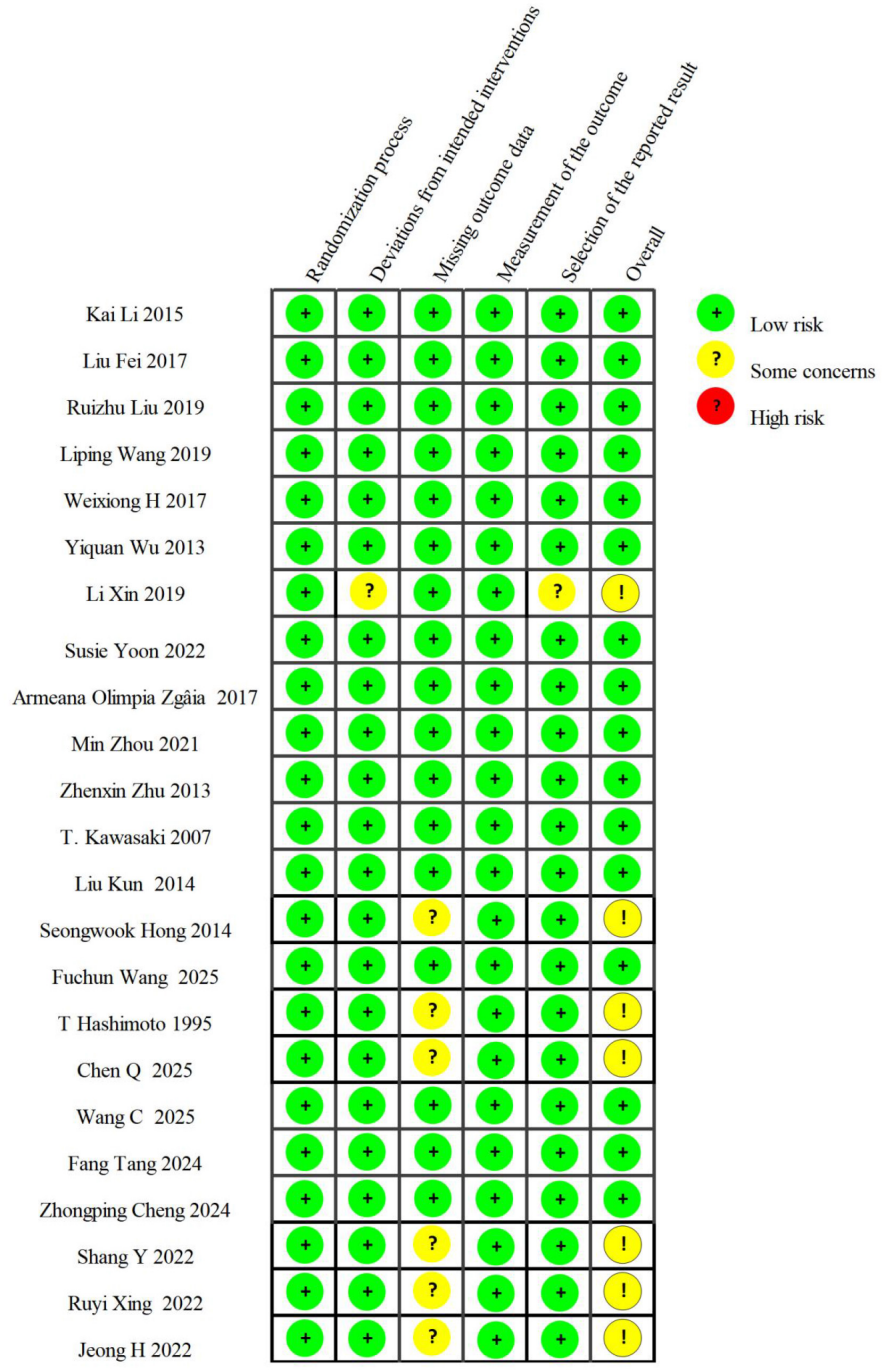
The risk of bias summery graph according to Cochrane risk of bias tool for randomized trials (RoB2).

Using the Cochrane Risk of Bias tool version 2.0 (RoB 2.0), 16 of the 23 included randomized controlled trials were judged to be at overall low risk of bias, whereas seven were rated as having some concerns, indicating generally acceptable methodological quality across studies. In the domain of bias arising from the randomization process, all trials reported the use of random number tables or computer-generated randomization methods and demonstrated baseline comparability between groups. Regarding deviations from intended interventions, a minority of studies did not clearly report whether personnel administering the interventions were blinded. This lack of clarity may have introduced performance bias, particularly with respect to intervention delivery or the administration of rescue analgesia. For missing outcome data, most trials reported high treatment completion rates and low attrition, with reasons for withdrawal adequately described. Although several studies did not provide detailed follow-up information, no clear evidence of selective exclusion of outcome data was identified, and overall data completeness was considered acceptable. In the domain of outcome measurement, the primary endpoint—pain intensity assessed using the VAS—is inherently subjective and may be susceptible to bias if participants or outcome assessors were aware of treatment allocation. Several studies did not clearly describe participant blinding, and in some cases, the procedures used to assess movement-related pain were insufficiently specified. With respect to selection of the reported results, a small number of trials did not provide publicly accessible protocols or prespecified statistical analysis plans. As a result, it was not possible to determine whether outcomes, time points, and analytical methods had been defined *a priori*, and selective reporting bias could not be excluded (Figure 2).

### 3.3 Network meta-analysis results

#### 3.3.1 Consistency and inconsistency

The primary outcomes of this network meta-analysis were postoperative pain intensity at rest and during movement, assessed using the VAS in both the short-term and long-term postoperative periods. Secondary outcomes included the CD4^+^/CD8^+^ T-cell ratio, as an indicator of cellular immune function, and cumulative postoperative opioid consumption. Closed loops were present in the treatment networks for short-term postoperative pain at rest, long-term postoperative pain at rest, long-term postoperative pain during movement, and opioid consumption, enabling formal assessment of inconsistency. Global inconsistency was evaluated using the design-by-treatment interaction model, and all corresponding *P* values were greater than 0.05 (Supplementary Table S11), indicating no evidence of statistically significant global inconsistency and supporting the assumption of consistency across the networks. Local inconsistency was further assessed using the node-splitting approach. For all pairwise comparisons, *P* values exceeded 0.05 (Supplementary Tables S12–S15), suggesting no statistically significant disagreement between direct and indirect estimates at the comparison level. Loop-specific inconsistency was evaluated using IFs. Two loops showed 95% CIs for the IF that did not include zero: EA–EspB–SA in short-term VAS pain scores at rest (95% CI: 0.38 to 1.57) and EA–SA–TAP in long-term VAS pain scores during movement (95% CI: 0.26 to 1.65). These findings indicate potential inconsistency between direct and indirect evidence within these specific loops and should be interpreted with caution. For all other loops across outcomes, the 95% CIs of the inconsistency factors included zero, indicating no statistically significant evidence of inconsistency (Supplementary Figures S1–S4).

Because the treatment networks for Short-term VAS pain scores during movement and the CD4^+^/CD8^+^ T-cell ratio did not contain closed loops, formal assessment of global, local, or loop-specific inconsistency was not feasible. For these outcomes, network meta-analyses were conducted under the assumption of consistency using a random-effects consistency model. The plausibility of the transitivity assumption was evaluated by comparing key clinical and methodological characteristics across studies. As summarized in Table 1 and Supplementary Table S2, baseline characteristics—including patient age, intervention characteristics, and follow-up duration—were broadly comparable across treatment comparisons, supporting the assumption of transitivity. However, because the network structure relied primarily on indirect comparisons and did not permit formal inconsistency assessment, the certainty of evidence for these outcomes was downgraded for indirectness within the GRADE–CINeMA framework. Accordingly, these findings should be interpreted with caution.

#### 3.3.2 Short-term VAS pain scores at rest

**Figure 3:**
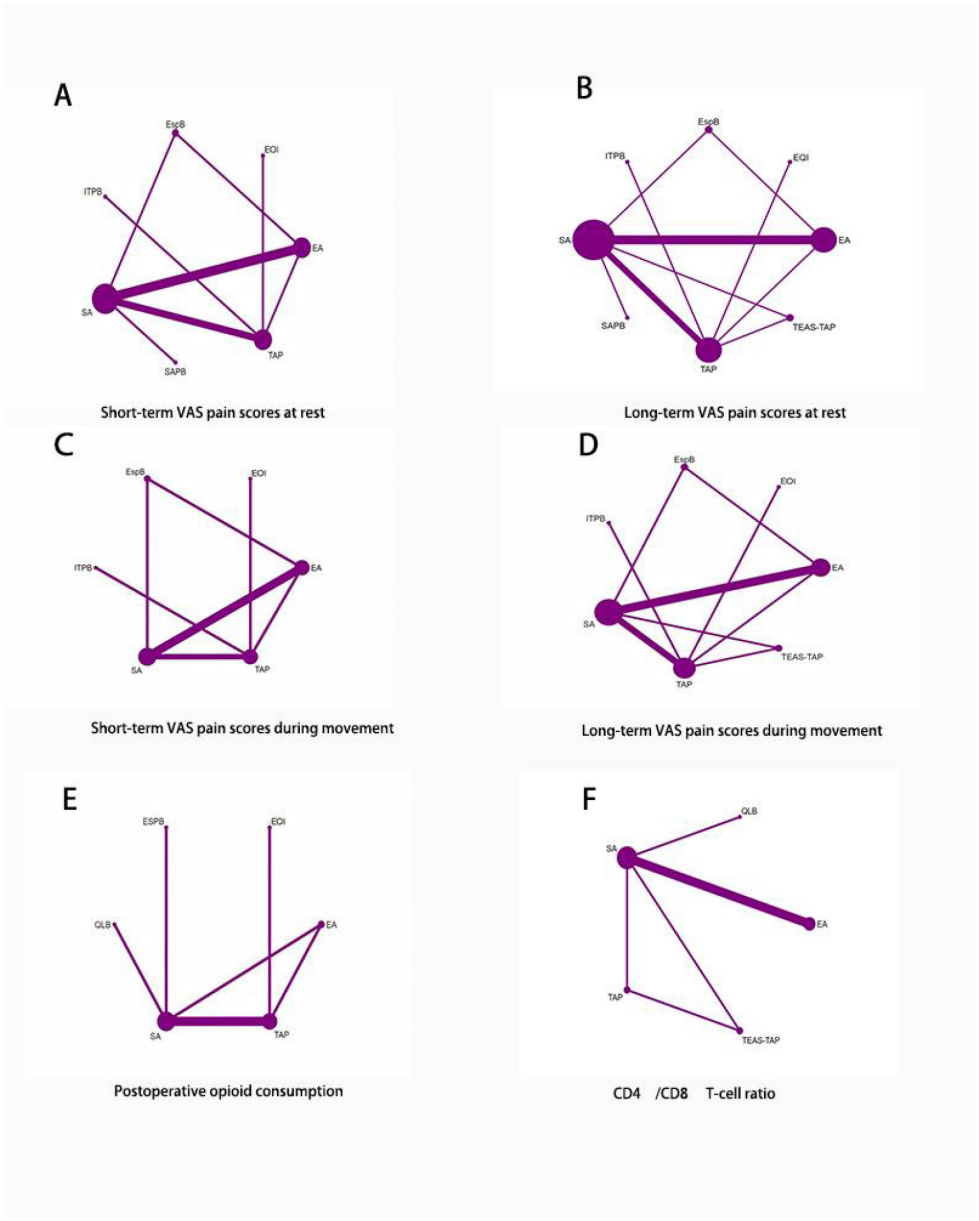
Network plot of treatment comparisons. **(A)** Short-term VAS pain scores at rest; **(B)** long-term VAS pain scores at rest; **(C)** short-term VAS pain scores during movement; **(D)** long-term VAS pain scores during movement; **(E)** postoperative opioid consumption; **(F)** CD4^+^/CD8^+^ T-cell ratio.

**Figure 4:**
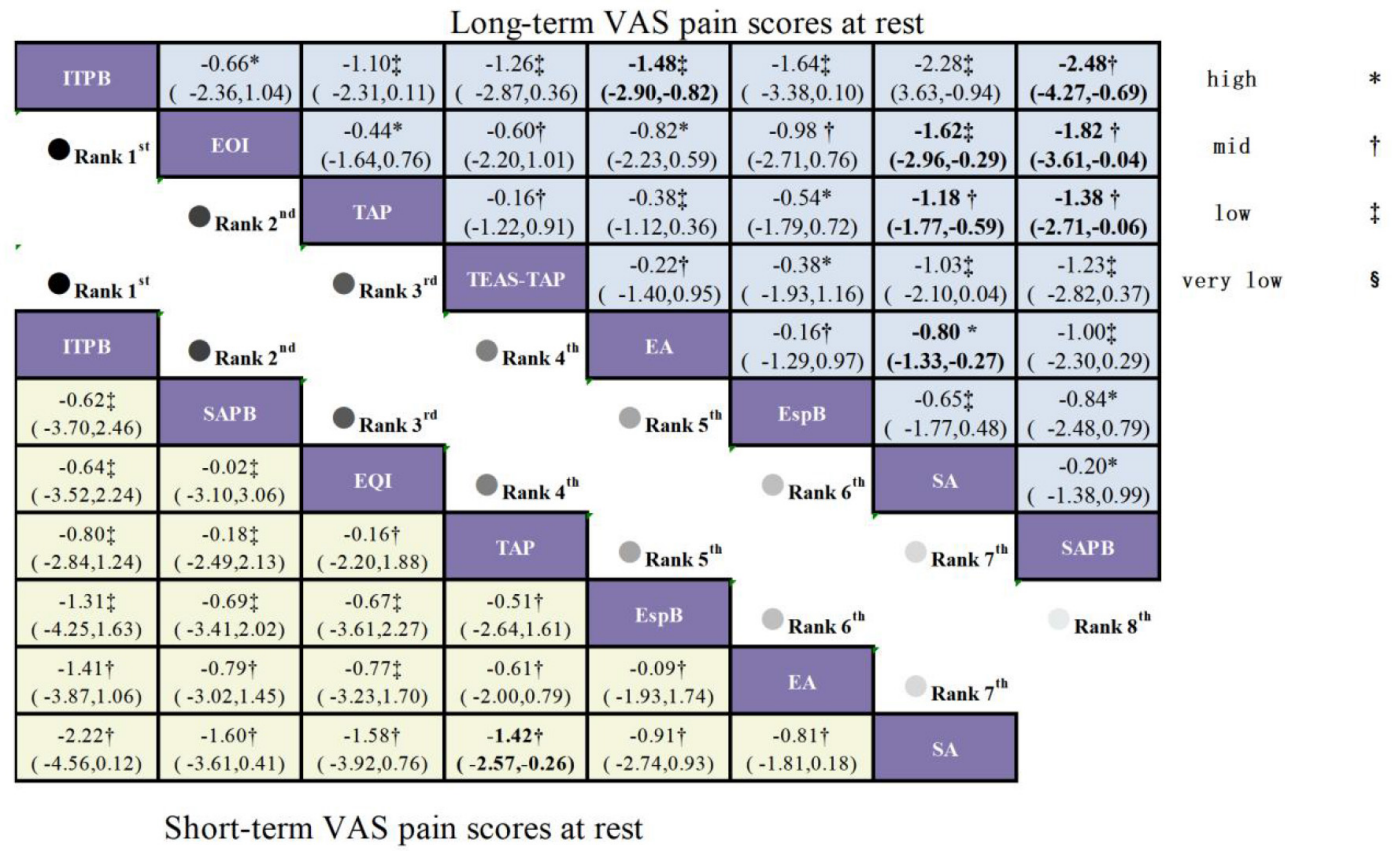
League table for short-term and long-term VAS pain scores at rest.

Nine trials comprising 668 patients and seven interventions contributed to the network for short-term postoperative pain at rest (Figure 3A). Compared with SA, TAP was associated with a statistically significant reduction in pain intensity (MD = −1.42; 95% CI, −2.57 to −0.36), with moderate-certainty evidence. Although ITPB, SAPB, and EOI were associated with lower pain intensity compared with SA, these differences did not reach statistical significance (ITPB: MD = −2.22; 95% CI, −4.56 to 0.12; SAPB: MD = −1.60; 95% CI, −3.61 to 0.41; EOI: MD = −1.58; 95% CI, −3.92 to 0.76), and the certainty of evidence for these comparisons was moderate (Figure 4).

#### 3.3.3 Long-term VAS pain scores at rest

In the assessment of long-term resting VAS scores, 12 studies involving 855 patients and 8 interventions were included (Figure 3B). Compared with SA, ITPB (MD = −2.28; 95% CI, −3.63 to −0.94), EOI (MD = −1.62; 95% CI, −2.96 to −0.29), TAP (MD = −1.18; 95% CI, −1.77 to −0.59), and EA (MD = −0.80; 95% CI, −1.33 to −0.27) significantly reduced VAS scores. The evidence for EA was rated as high, TAP as moderate, and ITPB and EOI as low. In contrast, TEAS-TAP (MD = −1.03; 95% CI, −2.10 to 0.04) and EspB (MD = −0.65; 95% CI, −1.77 to 0.48) showed trends toward reducing VAS scores, but the differences were not statistically significant and were rated as low-certainty evidence (Figure 4).

#### 3.3.4 Short-term VAS pain scores during movement

**Figure 5:**
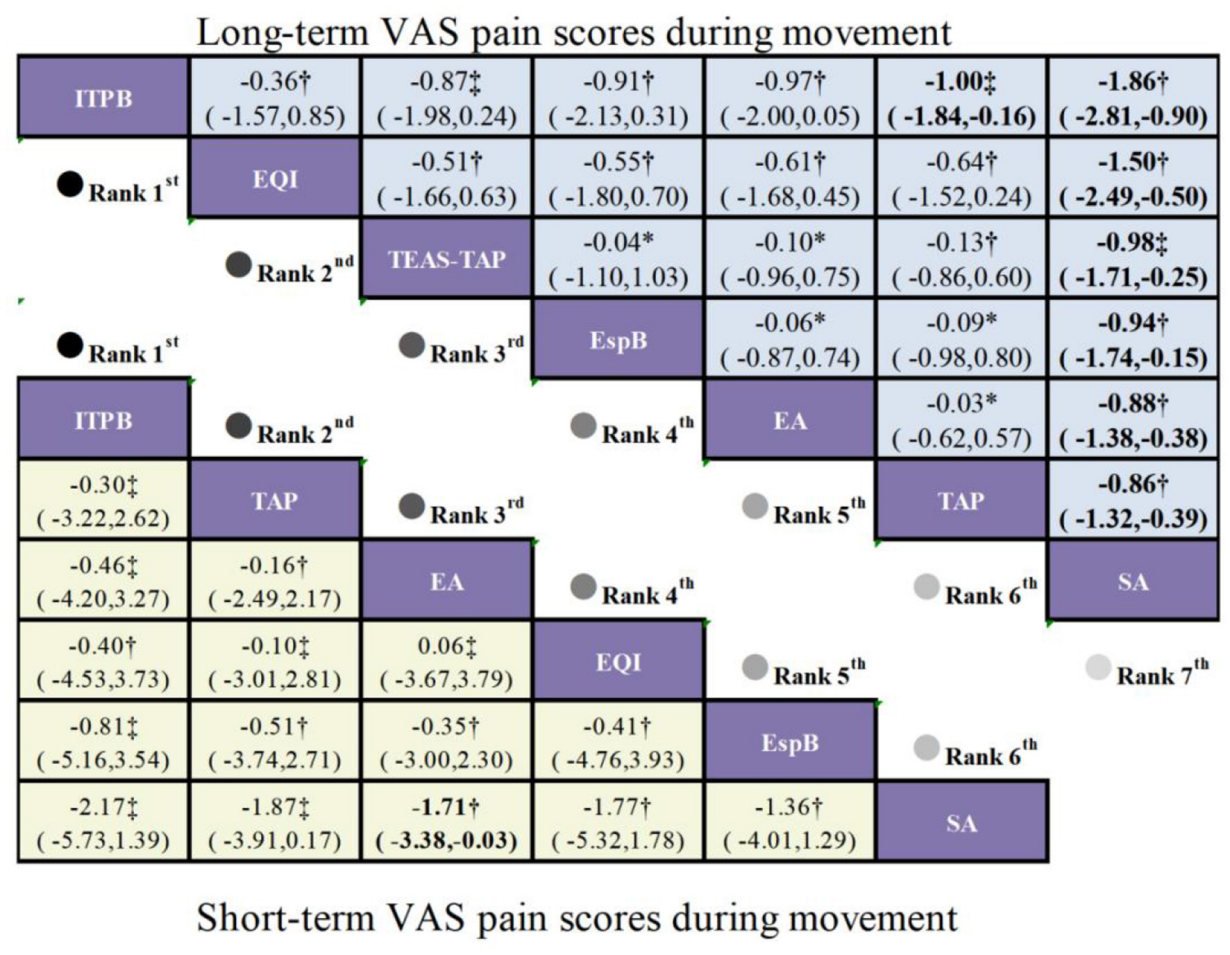
League table for short-term and long-term VAS pain scores during movement. *High certainty evidence. ^†^Moderate certainty evidence. ^‡^Low certainty evidence.

Six trials comprising 428 patients and six interventions contributed to the network for short-term postoperative pain during movement (Figure 3C). Compared with SA, EA was associated with a statistically significant reduction in pain intensity (MD = −1.71; 95% CI, −3.38 to −0.03), with moderate-certainty evidence. Although ITPB, TAP, EOI and EspB were associated with lower pain intensity compared with SA, these differences did not reach statistical significance (ITPB: MD = −2.17; 95% CI, −5.73 to 1.39; TAP: MD = −1.87; 95% CI, −3.91 to 0.17; EOI: MD = −1.77; 95% CI, −5.32 to 1.78; EspB: MD = −1.36; 95% CI, −4.01 to 1.29). The certainty of evidence was low for ITPB and moderate for the remaining comparisons (Figure 5).

#### 3.3.5 Long-term VAS pain scores during movement

Eight trials comprising 575 patients and seven interventions contributed to the network for long-term postoperative pain during movement (Figure 3D). Compared with SA, several regional analgesia techniques were associated with statistically significant reductions in pain intensity. ITPB demonstrated the largest reduction in pain intensity (MD= −1.86; 95% CI, −2.81 to −0.90), followed by EOI (MD = −1.50; 95% CI, −2.49 to −0.50), TEAS–TAP (MD = −0.98; 95% CI, −1.71 to −0.25), EspB (MD = −0.94; 95% CI, −1.74 to −0.15), EA (MD = −0.88; 95% CI, −1.38 to −0.38), and TAP alone (MD = −0.86; 95% CI, −1.32 to −0.39). The certainty of evidence was moderate for all comparisons except TEAS–TAP, which was supported by low-certainty evidence (Figure 5).

#### 3.3.6 Postoperative opioid consumption

**Figure 6:**
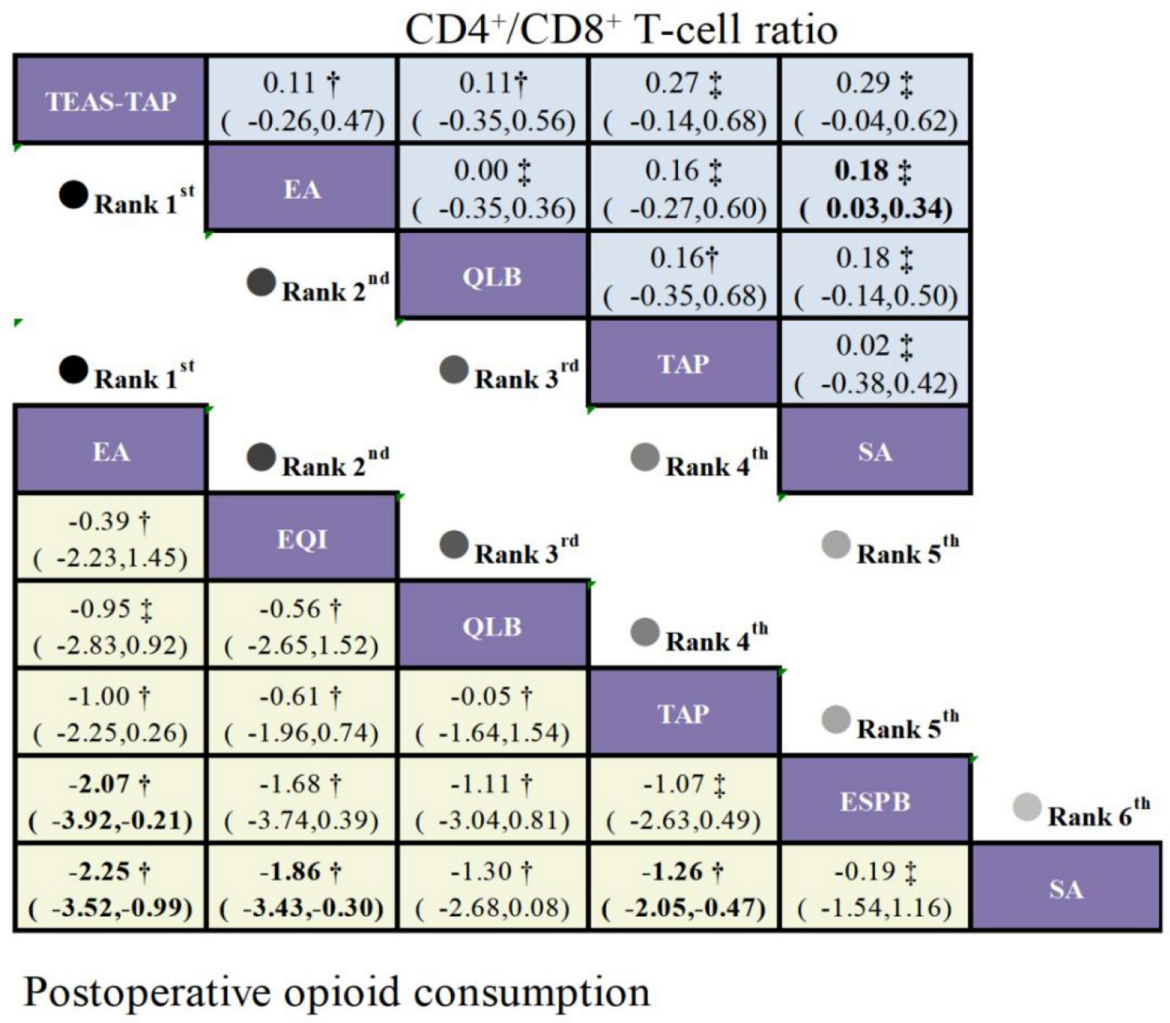
League table for postoperative opioid consumption and CD4^+^/CD8^+^ T-cell ratio. *High certainty evidence. ^†^Moderate certainty evidence. ^‡^Low certainty evidence.

Six trials comprising 376 patients and six interventions contributed to the network for postoperative opioid consumption (Figure 3E). Compared with SA, EA was associated with a statistically significant reduction in cumulative opioid consumption (SMD= −2.25; 95% CI, −3.52 to −0.99), as were EOI (SMD = −1.86; 95% CI, −3.43 to −0.30) and TAP (SMD =-1.26; 95% CI, −2.05 to −0.47). The certainty of evidence for these comparisons was moderate. QLB was associated with a reduction in opioid consumption compared with SA (SMD = −1.30; 95% CI, −2.68 to 0.08), although the confidence interval crossed the null value, indicating no statistically significant difference (moderate-certainty evidence). EspB was not associated with a statistically significant difference in opioid consumption (SMD = −0.19; 95% CI, −1.54 to 1.16; low-certainty evidence; Figure 6).

#### 3.3.7 CD4+/CD8+T-cell ratio

Six trials comprising 481 patients and five interventions contributed to the network evaluating the CD4^+^/CD8^+^ T-cell ratio (Figure 3F). Compared with SA, EA was associated with a statistically significant increase in the CD4^+^/CD8^+^ T-cell ratio (MD = 0.18; 95% CI, 0.03 to 0.34), with low-certainty evidence. TEAS–TAP, QLB, and TAP alone were associated with higher CD4^+^/CD8^+^ T-cell ratios compared with SA; however, these differences did not reach statistical significance (TEAS–TAP: MD = 0.29; 95% CI, −0.04 to 0.62; QLB: MD = 0.18; 95% CI, −0.14 to 0.50; TAP: MD = 0.02; 95% CI, −0.38 to 0.42), and the certainty of evidence for these comparisons was low (Figure 6).

### 3.4 Interpretation of SUCRA rankings

**Figure 7:**
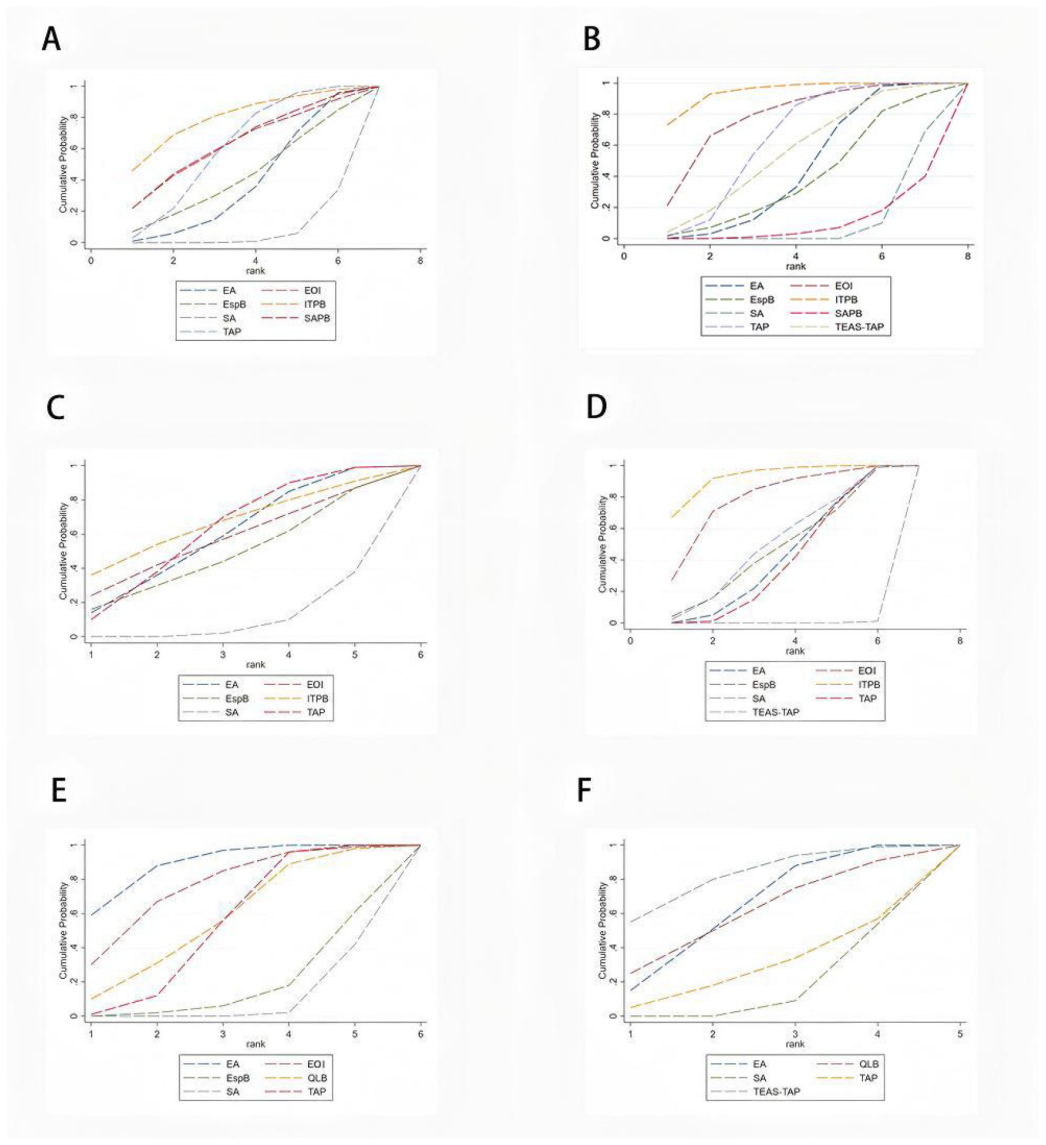
SUCRA diagrams for different outcome measures. SUCRA, surface under the cumulative ranking curve; **(A)** short-term VAS pain scores at rest; **(B)** long-term VAS pain scores at rest; **(C)** short-term VAS pain scores during movement; **(D)** long-term VAS pain scores during movement; **(E)** postoperative opioid consumption; **(F)** CD4^+^/CD8^+^ T-cell ratio.

According to the SUCRA ranking probabilities, ITPB showed the most favorable ranking profile for short-term VAS pain scores at rest (SUCRA = 79.3%), followed by SAPB (62.8%) and EOI (61.8%; Figure 7A; Supplementary Table S16). For long-term VAS pain scores at rest, SUCRA rankings indicated that ITPB (94.3%) ranked highest, followed by EOI (78.4%) and TAP (64.2%; Figure 7B; Supplementary Table S17). For short-term VAS pain scores during movement, ITPB again showed the highest ranking probability (SUCRA = 65.8%), followed by TAP (61.2%) and EA (58.6%; Figure 7C; Supplementary Table S18). Similarly, for long-term VAS pain scores during movement, ITPB remained the highest-ranked intervention (SUCRA = 66.9%), followed by TEAS–TAP (50.6%) and EOI (26.6%; Figure 7D; Supplementary Table S19). With respect to postoperative opioid consumption, EA demonstrated the greatest probability of ranking as the optimal intervention (SUCRA = 88.5%), followed by EOI (75.6%) and QLB (56.7%; Figure 7E; Supplementary Table S20). For the CD4^+^/CD8^+^ ratio, TEAS–TAP showed the highest ranking probability (SUCRA = 81.8%), followed by EA (63.5%) and QLB (60.3%; Figure 7F; Supplementary Table S21). However, because the certainty of evidence for several outcomes and comparisons was low-to-moderate, these SUCRA-based rankings should be interpreted cautiously and considered exploratory rather than definitive evidence of treatment superiority.

### 3.5 Sensitivity analyses, network meta-regression, and publication bias

The robustness of the network estimates was evaluated using leave-one-out sensitivity analyses, in which each study was sequentially excluded and the random-effects consistency model was re-estimated using the remaining data. This approach was applied across all outcomes to assess the influence of individual trials on the pooled network estimates. For the primary outcome of long-term postoperative pain during movement, the leave-one-out analyses did not identify any individual study exerting a disproportionate influence on the overall findings. Exclusion of any single trial did not alter the direction of treatment effects for any intervention compared with SA. The magnitude of the pooled estimates remained largely unchanged, with substantial overlap in confidence intervals across iterations, and the statistical significance of the comparisons was not materially affected. Overall, these findings indicate that the network estimates were robust and not driven by any single study (Supplementary Tables S22–S27). Where potential inconsistency or heterogeneity was identified within specific network loops, additional sensitivity analyses and network meta-regression analyses were performed to explore possible sources of instability. However, no individual study or evaluated covariate was identified as a major contributor to inconsistency or instability across outcomes.

To explore potential sources of effect modification, univariable network meta-regression analyses were performed across all outcomes. For short-term postoperative pain, prespecified covariates included the timing of outcome assessment (approximately 1 h postoperatively) and mean patient age, and surgical approach (laparoscopic vs. open gastrectomy). For long-term postoperative pain outcomes, mean patient age and surgical approach were included as covariates. For postoperative opioid consumption, covariates included opioid type (morphine vs. other agents), mean patient age, study country, and surgical approach. For the CD4^+^/CD8^+^ T-cell ratio, mean patient age and study country were evaluated as potential effect modifiers. These covariates were examined to assess their association with treatment effects relative to the reference comparator, SA. No statistically significant effect modification was identified for any of the evaluated covariates across outcomes, and none of the examined variables were significantly associated with treatment effects compared with SA (Supplementary Tables S28–S49).

Comparison-adjusted funnel plots were constructed for each outcome to assess potential small-study effects and publication bias. Visual inspection of the funnel plots showed no clear evidence of asymmetry or extreme outliers, and the distribution of studies appeared broadly symmetrical. These findings suggest no apparent evidence of substantial small-study effects or publication bias (Supplementary Figures S5–S10).

### 3.6 Certainty of evidence (GRADE–CINeMA)

The certainty of evidence for network estimates was evaluated using the GRADE framework implemented through the CINeMA approach. Across all outcomes and treatment comparisons, 13 estimates were rated as high certainty, 57 as moderate certainty, and 40 as low certainty; no comparisons were rated as very low certainty. High-certainty evidence was generally supported by multiple direct randomized controlled trials with consistent findings and relatively narrow confidence intervals that did not cross either the null value or the prespecified MID. In contrast, lower-certainty ratings were primarily attributable to network structures with limited direct evidence or greater reliance on indirect comparisons, often accompanied by wider confidence intervals and, in some cases, concerns related to imprecision or small-study effects (Supplementary Tables S50–S55).

## 4 Discussion

### 4.1 Principal findings

To our knowledge, this is the first network meta-analysis to comprehensively evaluate perioperative regional analgesia strategies in patients undergoing radical gastrectomy for gastric cancer. We included 23 randomized controlled trials comprising 1,700 patients and 11 interventions, integrating both direct and indirect evidence to compare the effects of regional analgesia techniques on postoperative pain intensity, opioid consumption, and recovery of cellular immune function (20–42). Evidence certainty was comprehensively evaluated using SUCRA rankings and the CINeMA/GRADE framework. Significant differences in pain management and immune modulation were observed among the regional techniques, though most comparisons were supported by moderate to low-certainty evidence. For pain outcomes, ITPB consistently ranked highly in both short- and long-term VAS scores, suggesting potentially favorable analgesic performance across multiple postoperative pain outcomes, although these findings remain hypothesis-generating given the limited certainty of evidence. Certain fascial plane blocks (e.g., TAP, EspB) showed trends toward better outcomes compared to systemic analgesia (SA), but these techniques ranked lower and were predominantly supported by moderate to low-certainty evidence. Regarding immune function, TEAS-TAP ranked highly for restoring the CD4^+^/CD8^+^ ratio, although the evidence certainty was low and the finding remains exploratory. Overall, our findings provide the best available evidence for optimizing regional analgesia strategies in gastric cancer surgery. However, further high-quality randomized controlled trials are necessary to confirm these results. In addition, variability across studies in surgical approach (open vs. laparoscopic gastrectomy), perioperative analgesic protocols, and regional block techniques may also have contributed to differences in treatment effects across the included trials. Although network meta-regression analyses did not identify statistically significant effect modification, these clinical and methodological differences should be considered when interpreting the present findings.

### 4.2 Interpretation of findings

With respect to pain outcomes, ITPB consistently ranked among the most effective interventions across short-term postoperative pain at rest, long-term postoperative pain at rest, short-term pain during movement, and long-term pain during movement, suggesting a stable analgesic profile across clinically relevant time points and functional conditions. Collectively, these findings suggest that ITPB may provide both broad and sustained analgesic coverage in patients undergoing radical gastrectomy. The analgesic performance of ITPB is anatomically and physiologically plausible. Postoperative pain following gastrectomy arises primarily from the anterior branches of the T6–T10 intercostal nerves and visceral afferent input from the upper abdominal wall. Injection within the intertransverse process plane allows local anesthetic to spread toward the thoracic paravertebral space and adjacent sympathetic chain, potentially blocking both somatic and visceral nociceptive pathways. This dual mechanism may explain the consistent analgesic benefit observed across multiple postoperative pain endpoints (43). These mechanistic considerations are consistent with the ranking patterns observed in the present network meta-analysis. Nevertheless, because treatment rankings are probabilistic and several comparisons were supported by limited certainty of evidence, these findings should be interpreted as hypothesis-generating rather than definitive proof that ITPB is superior to other regional analgesia techniques. With regard to postoperative opioid consumption, EA demonstrated the most pronounced opioid-sparing effect, consistent with its established role as a highly effective regional analgesic technique. By acting at the level of the spinal dorsal horn, epidural local anesthetics inhibit nociceptive transmission and reduce central sensitization. However, epidural analgesia is also associated with well-recognized adverse effects, including hypotension, urinary retention, and motor blockade, which may limit its applicability in certain patients (44). In contrast, fascial plane blocks such as QLB and TAP block may provide effective analgesia with fewer systemic effects, simpler technical performance, and greater hemodynamic stability. These characteristics may make fascial plane blocks particularly well suited for incorporation into ERAS protocols, which emphasize effective analgesia while minimizing procedure-related morbidity and promoting early mobilization (45). Notably, in the analysis of immunologic outcomes, TEAS–TAP ranked highest for improvement in the CD4^+^/CD8^+^ T-cell ratio. Surgical stress and uncontrolled postoperative pain are known to contribute to perioperative immunosuppression through activation of the sympathetic–adrenal axis and release of pro-inflammatory mediators, which may impair cellular immune function and potentially influence oncologic outcomes (46, 47). TEAS may modulate these responses through neuromodulatory pathways, including activation of endogenous opioid systems and attenuation of neuroendocrine stress signaling, thereby promoting recovery of T-lymphocyte subset balance (48). Although this finding suggests a potential immunomodulatory role for adjunctive neuromodulation strategies, these results should be interpreted cautiously given the limited certainty of evidence.

### 4.3 Implications for clinical practice

The present findings highlight the important role of regional analgesia as a key component of perioperative care in patients undergoing radical gastrectomy for gastric cancer. Beyond providing effective pain relief, regional techniques may contribute to enhanced postoperative recovery by reducing opioid exposure, facilitating early mobilization, and supporting multimodal analgesia strategies. While traditional perioperative analgesia has relied heavily on epidural analgesia or systemic opioid-based regimens, the present network meta-analysis provides a comparative evaluation of multiple regional approaches by integrating both direct and indirect evidence, thereby offering a more comprehensive foundation for individualized analgesic planning. Several regional techniques, particularly fascial plane blocks, offer practical and clinically relevant advantages. These include technical feasibility, minimal hemodynamic effects, meaningful opioid-sparing benefits, and a favorable safety profile. Such characteristics align closely with the principles of ERAS pathways, which emphasize effective analgesia while minimizing procedure-related morbidity and promoting early functional recovery. As a result, these techniques may represent viable alternatives or complements to epidural analgesia in appropriately selected patients. Importantly, different regional analgesia techniques may offer distinct clinical benefits depending on specific perioperative priorities. ITPB demonstrated consistently favorable performance across multiple pain outcomes and treatment rankings, suggesting that it may provide effective and sustained analgesia. In addition, TEAS–TAP ranked favorably in analyses of immune recovery, suggesting a potential adjunctive role in perioperative immune modulation, although this finding should be interpreted cautiously given the limited certainty of evidence. Despite these findings, the certainty of evidence for many comparisons was moderate or low, and no single technique demonstrated clear superiority across all clinically relevant outcomes. Accordingly, selection of regional analgesia should be individualized, taking into account patient characteristics, surgical factors, analgesic goals, risk of opioid-related adverse effects, technical expertise, and institutional resources. Incorporation of regional analgesia into multimodal perioperative care pathways should be guided by a balanced assessment of potential benefits and risks, as well as patient preferences. Further high-quality randomized controlled trials are needed to refine the optimal selection and implementation of regional analgesia techniques in this patient population.

### 4.4 Comparison with previous evidence

Compared with conventional pairwise meta-analyses, the present study used a network meta-analytic framework to integrate both direct and indirect evidence, allowing simultaneous comparison of multiple regional analgesia techniques in the absence of extensive head-to-head randomized trials. This approach enabled estimation of relative treatment effects across a broad range of interventions and facilitated clinically interpretable treatment rankings using SUCRA probabilities. As a result, this analysis provides a more comprehensive comparative assessment than traditional pairwise meta-analyses, which are limited to isolated comparisons between two interventions. Previous systematic reviews have primarily evaluated individual regional techniques or focused on specific surgical populations. For example, Hamid et al. reported comparable analgesic efficacy between TAP block and thoracic epidural analgesia in colorectal surgery, with TAP block associated with fewer complications and improved early functional recovery (49). Other meta-analyses have demonstrated that TAP block reduces postoperative pain intensity and opioid consumption across various abdominal procedures (50). Similarly, comparative analyses of QLB and TAP block in gynecologic surgery have suggested differences in analgesic efficacy depending on surgical context and outcome timing (51). However, these studies were generally restricted to pairwise comparisons involving one or two techniques, limiting their ability to inform clinical decision-making when multiple regional analgesia options are available. The present study expands upon prior evidence in several important respects. First, we evaluated 11 regional analgesia strategies, enabling a broader and more clinically relevant comparison across commonly used techniques. Second, outcomes were assessed across multiple clinically meaningful dimensions, including pain intensity at rest and during movement, short- and long-term postoperative time points, cumulative opioid consumption, and recovery of cellular immune function as reflected by the CD4^+^/CD8^+^ T-cell ratio. This multidimensional assessment provides a more comprehensive evaluation of analgesic effectiveness and perioperative recovery than analyses limited to pain outcomes alone. Third, unlike previous reviews that predominantly examined colorectal or gynecologic surgery, this analysis specifically focused on patients undergoing radical gastrectomy for gastric cancer. This population may exhibit distinct postoperative pain profiles and immunologic responses due to the extent of surgical dissection, perioperative stress, and underlying malignancy-related immune dysregulation. Taken together, the present network meta-analysis extends the existing literature by providing a comprehensive, procedure-specific comparison of regional analgesia techniques in gastric cancer surgery. These findings complement prior evidence and offer a more detailed and clinically applicable framework to support perioperative analgesic decision-making in this patient population.

### 4.5 Strengths and limitations

This study has several important methodological strengths. First, we evaluated a broad range of clinically relevant outcomes, including postoperative pain intensity, cumulative opioid consumption, and immunologic parameters, allowing a more comprehensive assessment of perioperative recovery beyond analgesic efficacy alone. Second, the use of a network meta-analysis framework enabled simultaneous comparison of multiple regional analgesia techniques by integrating both direct and indirect evidence, thereby enhancing the scope and clinical relevance of the findings. Third, the validity of the network estimates was rigorously evaluated. Consistency assumptions were examined using global inconsistency testing, node-splitting analyses, and loop-specific inconsistency assessment, while robustness was supported through leave-one-out sensitivity analyses and network meta-regression exploring potential sources of effect modification. In addition, risk of bias was assessed using the RoB 2.0, and the certainty of evidence was evaluated using the CINeMA approach in conjunction with GRADE, strengthening the methodological transparency and credibility of the results. Several limitations should also be considered. First, although network meta-analysis enables indirect comparisons, the certainty of evidence for many comparisons was moderate or low, primarily due to limited sample sizes and reliance on indirect evidence in parts of the network. Second, heterogeneity in study design, including variation in regional analgesia techniques, timing of block administration, local anesthetic regimens, and perioperative analgesic protocols, may have contributed to between-study variability. Third, relatively few trials reported immunologic outcomes, limiting the precision and certainty of estimates related to immune function recovery. Fourth, most included studies were conducted in East Asian populations, which may affect the generalizability of the findings to other geographic regions and healthcare settings. Finally, although no clear evidence of publication bias was identified, the possibility of small-study effects cannot be completely excluded. Taken together, while this analysis provides a comprehensive and methodologically robust synthesis of available evidence, the findings should be interpreted in the context of these limitations, and further high-quality, adequately powered randomized controlled trials are warranted to confirm the comparative effectiveness of regional analgesia techniques in this clinical setting.

Despite these strengths, several limitations should be considered. First, some interventions—particularly ITPB, TEAS–TAP, and QLB—were supported by a limited number of trials with relatively small sample sizes, which may have reduced the precision and certainty of the corresponding estimates. Second, not all included studies implemented rigorous blinding procedures. Because postoperative pain intensity assessed using the VAS is inherently subjective, the absence of adequate blinding may have introduced measurement bias. Third, clinical and methodological heterogeneity was present across trials, including differences in surgical approach, local anesthetic regimens, timing of block administration, and concomitant analgesic protocols, which may have contributed to variability in treatment effects. In minimally invasive gastrectomy, local and fascial plane blocks may reduce postoperative pain primarily by attenuating somatic pain originating from trocar sites, upper abdominal wall incisions, and abdominal wall manipulation. However, their ability to control visceral pain associated with pneumoperitoneum, gastric mobilization, lymphadenectomy, and peritoneal irritation may be limited. Therefore, although regional blocks may provide clinically meaningful opioid-sparing and analgesic benefits after laparoscopic surgery, they may not be sufficient as standalone analgesic strategies in all patients. In contrast, open gastrectomy is generally associated with larger abdominal incisions, more extensive tissue injury, and greater postoperative pain intensity. Therefore, postoperative pain management in open surgery has traditionally relied more heavily on epidural analgesia or systemic opioid-based patient-controlled analgesia because these approaches provide broader somatic and visceral analgesic coverage. Although local and fascial plane blocks are increasingly incorporated into multimodal analgesia pathways, they are more commonly used as opioid-sparing adjuncts rather than complete substitutes for epidural or systemic analgesia in open gastrectomy. These differences in surgical approach and analgesic strategy may have contributed to clinical heterogeneity across the included studies and should be considered when interpreting the present findings. In addition, variations in regional analgesia delivery strategies, including single-shot vs. continuous techniques, may represent potential sources of clinical heterogeneity across studies, although no statistically significant effect modification was identified in the network meta-regression analyses. Moreover, immunologic outcomes and longer-term follow-up data were infrequently reported, limiting assessment of sustained immune recovery and longer-term clinical implications. Furthermore, for certain outcomes, including short-term pain during movement and the CD4^+^/CD8^+^ T-cell ratio, the network lacked closed loops, and estimates were therefore based primarily on indirect comparisons. Although sensitivity analyses and network meta-regression did not identify major sources of instability, the absence of closed-loop structures limits full evaluation of network consistency. In particular, residual concerns regarding clinical heterogeneity, the transitivity assumption, and reliance on indirect comparisons in parts of the network may have influenced the robustness and interpretability of certain treatment estimates. Taken together, these limitations warrant cautious interpretation of the findings. Further large-scale, well-designed randomized controlled trials with standardized protocols and comprehensive outcome reporting are needed to strengthen the evidence base and clarify the comparative effectiveness of regional analgesia techniques in this population.

### 4.6 Conclusion

In summary, this network meta-analysis demonstrates that regional analgesia provides clinically meaningful pain relief and reduces opioid consumption after radical gastrectomy for gastric cancer. Among the evaluated techniques, ITPB demonstrated favorable ranking performance across postoperative pain outcomes, although the certainty of evidence for several comparisons remained limited. In addition to analgesic benefits, TEAS–TAP showed a potential advantage in promoting recovery of the CD4^+^/CD8^+^ T-cell ratio, although the certainty of evidence was limited. Overall, these findings support the role of regional analgesia as a key component of multimodal perioperative pain management. However, further large-scale, high-quality randomized controlled trials are needed to confirm these results and to clarify the comparative effectiveness and immunologic effects of individual techniques.

## Data Availability

Publicly available datasets were analyzed in this study. This data can be found at: The datasets analyzed in this study were derived from previously published randomized controlled trials. These articles are publicly available in scientific databases such as PubMed, EMBASE, the Cochrane Library, and Web of Science.
