# Supplementary material for: Comparative effectiveness of regional analgesia techniques after gastrectomy for gastric cancer: a systematic review and network meta-analysis of randomized trials
Source: Front Med (Lausanne). 2026 Jun 15;13:1829566. doi: 10.3389/fmed.2026.1829566 (PMC13311095; doi:10.3389/fmed.2026.1829566)
Supplement: Supplementary file 1 [file Data_Sheet_1.pdf]

Table S1 PRISMA NMA Checklist of Items to Include When Reporting a Systematic Review Involving a Network Meta-analysis

| Section/Topic | Item # | Checklist Item | Reported on Page # |
|---|---|---|---|
| **TITLE** | | | |
| Title | 1 | Identify the report as a systematic review *incorporating anetwork meta-analysis (or related form of meta-analysis).* | **1** |
| **ABSTRACT** | | | |
| Structured summary | 2 | Provide a structured summary including, as applicable: **Background:** main objectives **Methods:** data sources; study eligibility criteria, participants, and interventions; study appraisal; and *synthesis methods, such as network meta-analysis.* **Results:** number of studies and participants identified; summary estimates with corresponding confidence/credible intervals; *treatment rankings may also be discussed. Authors may choose to summarize pairwise comparisons against a chosen treatment included in their analyses for brevity.* **Discussion/Conclusions:** limitations; conclusions and implications of findings. **Other:** systematic review registration number with registry name. | 2 |
| **INTRODUCTION** | | | |
| Rationale | 3 | Describe the rationale for the review in the context of what is already known*, including mention of why a network meta-analysis has been conducted.*_ | **3** |
| Objectives | 4 | Provide an explicit statement of questions being addressed, with reference to participants, interventions, comparisons, outcomes, and study design (PICOS). | 3 |
| **METHODS** | | | |
| Protocol and registration | 5 | Indicate whether a review protocol exists and if and where it can be accessed (e.g., Web address); and, if available, provide registration information, including registration number. | 4 |
| Eligibility criteria | 6 | Specify study characteristics (e.g., PICOS, length of follow-up) and report characteristics (e.g., years considered, language, publication status) used as criteria for eligibility, giving rationale. *Clearly describe eligible treatments included in the treatment network, and note whether any have been clustered or merged into the same node (with justification).*_ | 5 |
| Information sources | 7 | Describe all information sources (e.g., databases with dates of coverage, contact with study authors to identify additional studies) in the search and date last searched. | 5 |
| Search | 8 | Present full electronic search strategy for at least one database, including any limits used, such that it could be repeated. | 3，Supplementary TableS2 |
| Study selection | 9 | State the process for selecting studies (i.e., screening, eligibility, included in systematic review, and, if applicable, included in the meta-analysis). | **35，** Figure 1 |
| Data collection process | 10 | Describe method of data extraction from reports (e.g., piloted forms, independently, in duplicate) and any processes for obtaining and confirming data from investigators. | 7 |
| Data items | 11 | List and define all variables for which data were sought (e.g., PICOS, funding sources) and any assumptions and simplifications made. | 7 |
| **Geometry of the network** | **S1** | Describe methods used to explore the geometry of the treatment network under study and potential biases related to it. This should include how the evidence base has been graphically summarized for presentation, and what characteristics were compiled and used to describe the evidence base to readers. | **8** |
| Risk of bias within individual studies | 12 | Describe methods used for assessing risk of bias of individual studies (including specification of whether this was done at the study or outcome level), and how this information is to be used in any data synthesis. | 9 |

| | | | |
|---|---|---|---|
| Summary measures | 13 | State the principal summary measures (e.g., risk ratio, difference in means). *Also describe the use of additional summary measures assessed, such as treatment rankings and surface under the cumulative ranking curve (SUCRA) values, as well as modified approaches used to present summary findings from meta-analyses.* | 8 |
| Planned methods of analysis | 14 | Describe the methods of handling data and combining results of studies for each network meta-analysis. This should include, but not be limited to:<br>• *Handling of multi-arm trials;*<br>• *Selection of variance structure;*<br>• *Selection of prior distributions in Bayesian analyses; and*<br>• *Assessment of model fit.* | 8 |
| **Assessment of Inconsistency** | **S2** | Describe the statistical methods used to evaluate the agreement of direct and indirect evidence in the treatment network(s) studied. Describe efforts taken to address its presence when found. | 8 |
| Risk of bias across studies | 15 | Specify any assessment of risk of bias that may affect the cumulative evidence (e.g., publication bias, selective reporting within studies). | 9 |
| Additional analyses | 16 | Describe methods of additional analyses if done, indicating which were pre-specified. This may include, but not be limited to, the following:<br>• Sensitivity or subgroup analyses;<br>• Meta-regression analyses;<br>• *Alternative formulations of the treatment network; and*<br>• *Use of alternative prior distributions for Bayesian analyses (if applicable).*_ | 8-9 |
| **RESULTS†** | | | |
| Study selection | 17 | Give numbers of studies screened, assessed for eligibility, and included in the review, with reasons for exclusions at each stage, ideally with a flow diagram. | 35，Fig1 |
| **Presentation of network structure** | **S3** | Provide a network graph of the included studies to enable visualization of the geometry of the treatment network. | 37，Fig 3 |
| **Summary of network geometry** | **S4** | Provide a brief overview of characteristics of the treatment network. This may include commentary on the abundance of trials and randomized patients for the different interventions and pairwise comparisons in the network, gaps of evidence in the treatment network, and potential biases reflected by the network structure. | 10 |
| Study characteristics | 18 | For each study, present characteristics for which data were extracted (e.g., study size, PICOS, follow-up period) and provide the citations. | 31-33,Table 1, Supplementary Table S4 |
| Risk of bias within studies | 19 | Present data on risk of bias of each study and, if available, any outcome level assessment. | 53-54,Supplementary Figure S1-4 |
| Results of individual studies | 20 | For all outcomes considered (benefits or harms), present, for each study: 1) simple summary data for each intervention group, and 2) effect estimates and confidence intervals. *Modified approaches may be needed to deal with information from larger networks.* | 10 |
| Synthesis of results | 21 | Present results of each meta-analysis done, including confidence/credible intervals. *In larger networks, authors may focus on comparisons versus a particular comparator (e.g. placebo or standard care), with full findings presented in an appendix. League tables and forest plots may be considered to summarize pairwise comparisons.* If additional summary measures were explored (such as treatment rankings), these should also be presented. | 13-16 |
| **Exploration for inconsistency** | **S5** | Describe results from investigations of inconsistency. This may include such information as measures of model fit to compare consistency and inconsistency models, *P* values from statistical tests, or summary of inconsistency estimates from different parts of the treatment network. | 12,Supplementary Table S5 |
| Risk of bias across studies | 22 | Present results of any assessment of risk of bias across studies for the evidence base being studied. | 11 |
| Results of additional analyses | 23 | Give results of additional analyses, if done (e.g., sensitivity or subgroup analyses, meta-regression analyses*, alternative network geometries studied, alternative choice of prior distributions for Bayesian analyses,* and so forth). | 16-17 |

| | | | |
|---|---|---|---|
| **DISCUSSION** | | | |
| Summary of evidence | 24 | Summarize the main findings, including the strength of evidence for each main outcome; consider their relevance to key groups (e.g., healthcare providers, users, and policy-makers). | **17-18** |
| Limitations | 25 | Discuss limitations at study and outcome level (e.g., risk of bias), and at review level (e.g., incomplete retrieval of identified research, reporting bias). *Comment on the validity of the assumptions, such as transitivity and consistency. Comment on any concerns regarding network geometry (e.g., avoidance of certain comparisons).* | 22 |
| Conclusions | 26 | Provide a general interpretation of the results in the context of other evidence, and implications for future research. | 23 |
| **FUNDING** | | | |
| Funding | 27 | Describe sources of funding for the systematic review and other support (e.g., supply of data); role of funders for the systematic review. This should also include information regarding whether funding has been received from manufacturers of treatments in the network and/or whether some of the authors are content experts with professional conflicts of interest that could affect use of treatments in the network. | 24 |

PICOS = population, intervention, comparators, outcomes, study design.

* Text in italics indicate S wording specific to reporting of network meta-analyses that has been added to guidance from the PRISMA statement.

† Authors may wish to plan for use of appendices to present all relevant information in full detail for items in this section.

## Table S2 Literature Search Strategy

| **Table S2.Literature Search Strategy** | |
|---|---|
| Pubmed | (("stomach neoplasms"[MeSH Terms] OR "neoplasm stomach"[Title/Abstract] OR "stomach neoplasm"[Title/Abstract] OR "gastric neoplasms"[Title/Abstract] OR "gastric neoplasm"[Title/Abstract] OR "neoplasm gastric"[Title/Abstract] OR "neoplasms gastric"[Title/Abstract] OR "neoplasms stomach"[Title/Abstract] OR "cancer of stomach"[Title/Abstract] OR "stomach cancers"[Title/Abstract] OR "cancer of the stomach"[Title/Abstract] OR "gastric cancer"[Title/Abstract] OR "cancer gastric"[Title/Abstract] OR "cancers gastric"[Title/Abstract] OR "gastric cancers"[Title/Abstract] OR "stomach cancer"[Title/Abstract] OR "cancers stomach"[Title/Abstract] OR "cancer stomach"[Title/Abstract] OR (("stomach neoplasms"[MeSH Terms] OR ("Stomach"[All Fields] AND "Neoplasms"[All Fields]) OR "stomach neoplasms"[All Fields] OR ("Gastric"[All Fields] AND "Cancer"[All Fields]) OR "gastric cancer"[All Fields]) AND "familial diffuse"[Title/Abstract])) AND ("surgical procedures, operative"[MeSH Terms] OR "operative procedure"[Title/Abstract] OR "procedure operative"[Title/Abstract] OR "procedures operative"[Title/Abstract] OR "operative surgical procedure"[Title/Abstract] OR "operative surgical procedures"[Title/Abstract] OR (("methods"[MeSH Terms] OR "methods"[All Fields] OR "Procedure"[All Fields] OR "methods"[MeSH Subheading] OR "Procedures"[All Fields] OR "procedural"[All Fields] OR "procedurally"[All Fields] OR "procedure s"[All Fields]) AND "operative surgical"[Title/Abstract]) OR "procedures operative surgical"[Title/Abstract] OR "surgical procedure operative"[Title/Abstract] OR "surgical procedures"[Title/Abstract] OR "procedures surgical"[Title/Abstract] OR "procedure surgical"[Title/Abstract] OR "surgical procedure"[Title/Abstract] OR (("Surgery"[MeSH Subheading] OR "Surgery"[All Fields] OR "surgical procedures, operative"[MeSH Terms] OR ("Surgical"[All Fields] AND "Procedures"[All Fields] AND "Operative"[All Fields]) OR "operative surgical procedures"[All Fields] OR "general surgery"[MeSH Terms] OR ("general"[All Fields] AND "Surgery"[All Fields]) OR "general surgery"[All Fields] OR "surgery s"[All Fields] OR "surgerys"[All Fields] OR "surgeries"[All Fields]) AND "Ghost"[Title/Abstract]) OR "ghost surgery"[Title/Abstract]) AND ("anesthesia, conduction"[MeSH Terms] OR "conduction anesthesia"[Title/Abstract] OR "anesthesia regional"[Title/Abstract] OR "regional anesthesia"[Title/Abstract])) |
| Web of Science | #1 TS=("Gastric Neoplasms" or "Neoplasm, Stomach" or "Stomach Neoplasm" or "Gastric Neoplasms" or "Gastric Neoplasm" or "Neoplasm, Gastric" or "Neoplasms, Gastric" or "Neoplasms, Stomach" or"Cancer of Stomach" or"Stomach Cancers" or "Cancer of the Stomach" or "Gastric Cancer" or"Cancer, Gastric" or "Cancers, Gastric" or "Gastric Cancers" or"Stomach Cancer" or"Cancers, Stomach" or "Cancer, Stomach" or "Gastric Cancer, Familial Diffuse") 110513<br>#2 ((TS=("Gastric Neoplasms" or "Neoplasm, Stomach" or "Stomach Neoplasm" or "Gastric Neoplasms" or "Gastric Neoplasm" or "Neoplasm, Gastric" or "Neoplasms, Gastric" or "Neoplasms, Stomach" or"Cancer of Stomach" or"Stomach Cancers" or "Cancer of the Stomach" or "Gastric Cancer" or"Cancer, Gastric" or "Cancers, Gastric" or "Gastric Cancers" or"Stomach Cancer" or"Cancers, Stomach" or "Cancer, Stomach" or "Gastric Cancer, Familial Diffuse")) AND TS=("Surgery" or "Operative Procedure" or"Procedure, Operative" or "Procedures, Operative" or "Operative Surgical Procedure" or"Operative Surgical Procedures" or "Procedure, Operative Surgical" or"Procedures, Operative Surgical" or "Surgical Procedure, Operative" or "Surgical Procedures" or"Procedures, Surgical" or "Procedure, Surgical" or"Surgical Procedure" or "Surgery, Ghost" or "Ghost Surgery")) AND TS=("Anesthesia, Conduction" or "Conduction Anesthesia" or "Anesthesia, Regional" or "Regional Anesthesia") 11<br>#3 TS=(random* OR randomi* OR randoml OR placebo* OR shamOR trial OR "clinical trial" OR "controlled clinical trial"OR "double blind" OR "single blind" OR "triple blindOR crossover OR "cross over" OR |

| | |
|---|---|
| | "parallel group" OR cluster random* OR RCT) 3334263<br>#4 TS=( random* OR randomi* OR randomlyOR placebo* OR shamOR trial OR "clinical trial" OR "controlled clinical trial"OR "double blind" OR "single blind" OR "triple blind"OR crossover OR "cross over" OR "parallel group"OR cluster random*OR RCT) 3334263<br>#5 #2 and #3 7 |
| Cochrane | #1 MeSH descriptor: [Stomach Neoplasms] explode all trees 4281<br>#2 "Cancers, Stomach" OR "Gastric Cancer" OR "Cancer of the Stomach" OR "Cancer, Gastric" OR "Cancers, Gastric" OR " Cancer, Stomach" OR " Stomach Cancer" OR " Gastric Cancers" OR " Cancer of Stomach" OR "Stomach Cancers" OR " Gastric Cancer, Familial Diffuse" OR "Neoplasm, Gastric" OR " Neoplasms, Stomach" OR " Gastric Neoplasm" OR "Neoplasms, Gastric" OR " Gastric Neoplasms" OR "Neoplasm, Stomach" OR "Stomach Neoplasm" 9341<br>#3 #1 OR #2 10249<br>#4 MeSH descriptor: [Surgical Procedures, Operative] explode all trees 177132<br>#5 "Surgical Procedure;" OR "Surgical Procedure, Operative" OR "Operative Surgical Procedures" OR "Operative Surgical Procedure" OR "Procedures, Surgical" OR "Operative Procedures" OR " Procedure, Operative" OR " Procedures, Operative Surgical" OR "Procedures, Operative" OR "Surgical Procedures" OR " Procedure, Operative Surgical" OR "Operative Procedure" OR "Procedure, Surgical" OR "Surgery, Ghost" OR "Ghost Surgery" 35868<br>#6 MeSH descriptor: [Anesthesia, Conduction] explode all trees 13257<br>#7 "Regional Anesthesia" OR "Anesthesia, Regional" OR "Conduction Anesthesia" 7642<br>#8 #6 OR #7 19221<br>#9 #4 OR #5 190439<br>#10 #3 AND #8 AND #9 20 |
| Embase | #1 'stomach cancer'/exp OR 'cancer of the cardia' OR 'cancer of the gastric antrum' OR 'cancer of the gastric body' OR 'cancer of the gastric cardia' OR 'cancer of the gastric fundus' OR 'cancer, stomach' OR 'cardia cancer' OR 'gastric antral cancer' OR 'gastric antrum cancer' OR 'gastric body cancer' OR 'gastric cancer' OR 'gastric cardia cancer' OR 'gastric cardiac cancer' OR 'gastric malignancies' OR 'gastric malignancy' OR 'malignancies of the stomach' OR 'malignancy of the stomach' OR 'malignant gastric neoplasm' OR 'malignant gastric tumor' OR 'malignant neoplasm of the stomach' OR 'malignant neoplasms of the stomach' OR 'malignant tumor of the stomach' OR 'malignant tumors of the stomach' OR 'malignant tumour of the stomach' OR 'malignant tumours of the stomach' OR 'pyloric cancer' OR 'stomach malignancies' OR 'stomach malignancy' OR 'stomach cancer' 219045<br>#2 'surgery'/exp OR 'diagnosis, surgical' OR 'diagnostic techniques, surgical' OR 'operation' OR 'operation care' OR 'operative intervention' OR 'operative repair' OR 'operative restoration' OR 'operative surgery' OR 'operative surgical procedure' OR 'operative surgical procedures' OR 'operative treatment' OR 'research surgery' OR 'resection' OR 'resective surgery' OR 'specialties, surgical' OR 'surgery, operative' OR 'surgical care' OR 'surgical correction' OR 'surgical diagnosis' OR 'surgical diagnostic techniques' OR 'surgical exposure' OR 'surgical intervention' OR 'surgical management' OR 'surgical operation' OR 'surgical practice' OR 'surgical procedures, operative' OR 'surgical repair' OR 'surgical research' OR 'surgical restoration' OR 'surgical service' OR 'surgical speciality' OR 'surgical specialties' OR 'surgical specialty' OR 'surgical therapy' OR 'surgical treatment' OR 'surgery' 9927101<br>#3 'regional anesthesia'/exp OR 'anaesthesia regionalis' OR 'anaesthesia, conduction' OR 'anesthesia, conduction' OR 'conduction anaesthesia' OR 'conduction anesthesia' OR 'region anaesthesia' OR 'region anesthesia' OR 'regional anaesthesia' OR 'regional anesthesia'<br>#7 'clinical article'/de OR 'randomized controlled trial'/de 4845048<br>#8 #1 AND #2 AND #3 AND #7 51 |

**Table S3** Definitions of dynamic pain assessment across included trials

| No. | First Author (Year) | Dynamic pain definition |
|---|---|---|

| | | |
|---|---|---|
| 1 | Liu Fei 2017 | during coughing |
| 2 | Ruizhu Liu 2019 | during coughing or body rotation |
| 3 | Yiquan Wu 2013 | during coughing or turning |
| 4 | Zhenxin Zhu 2013 | during coughing |
| 5 | Chen Q 2025 | during coughing |
| 6 | Wang C 2025 | during movement |
| 7 | Fang Tang 2024 | during activity; coughing if activity was not feasible |
| 8 | Ruyi Xing 2022 | during movement |

## Table S4 Extracted data for Short-term VAS pain scores at rest

| id | t | n | mean | sd |
|---|---|---|---|---|
| 1 | TAP | 20 | 0.55 | 0.17 |
| 1 | SA | 20 | 2.13 | 0.11 |
| 2 | EA | 30 | 0.7 | 1 |
| 2 | SA | 31 | 3.5 | 2.1 |
| 3 | TAP | 30 | 2.2 | 0.9 |
| 3 | SA | 31 | 2.9 | 0.9 |
| 5 | EA | 70 | 2.11 | 0.29 |
| 5 | SA | 70 | 2.1 | 0.3 |
| 6 | TAP | 29 | 1.5 | 2.2 |
| 6 | EA | 27 | 3 | 0.741 |
| 6 | SA | 26 | 3 | 0.37 |
| 17 | ITPB | 31 | 1.9 | 0.8 |
| 17 | TAP | 31 | 2.7 | 0.9 |
| 18 | EspB | 34 | 1 | 0.9748 |
| 18 | EA | 34 | 1 | 0.8398 |
| 18 | SA | 34 | 2 | 0.6898 |

| | | | | |
|---|---|---|---|---|
| 19 | EOI | 30 | 2.77 | 0.9 |
| 19 | TAP | 30 | 2.93 | 0.78 |
| 20 | SAPB | 30 | 0.3 | 0.1 |
| 20 | SA | 30 | 1.9 | 0.7 |

Abbreviations: id, study identification number; t, treatment group; n, sample size; mean, mean outcome value; sd, standard deviation.

**Table S5** Extracted data for Long-term VAS pain scores at rest

| id | t | n | mean | sd |
|---|---|---|---|---|
| 1 | TAP | 20 | 1.49 | 0.15 |
| 1 | SA | 20 | 3.59 | 0.25 |
| 2 | EA | 30 | 1 | 0.8 |
| 2 | SA | 31 | 2.1 | 1.2 |
| 3 | TAP | 30 | 2.2 | 1 |
| 3 | SA | 31 | 2.7 | 1 |
| 4 | EA | 20 | 2 | 2.2 |
| 4 | SA | 20 | 3 | 2.2 |
| 5 | EA | 70 | 1.32 | 0.31 |
| 5 | SA | 70 | 2.55 | 0.57 |
| 6 | TAP | 29 | 1 | 0.741 |
| 6 | EA | 27 | 2 | 1.11 |
| 6 | SA | 26 | 2.25 | 0.741 |
| 11 | EA | 30 | 2.96 | 1.548 |
| 11 | SA | 30 | 3.99 | 1.496 |
| 17 | ITPB | 31 | 1.2 | 0.7 |
| 17 | TAP | 31 | 2.3 | 0.6 |
| 18 | EspB | 34 | 4 | 1.0347 |
| 18 | EA | 34 | 4 | 1.1997 |
| 18 | SA | 34 | 4.5 | 0.8998 |
| 19 | EOI | 30 | 1.23 | 0.43 |
| 19 | TAP | 30 | 1.67 | 0.71 |
| 20 | SAPB | 30 | 3.5 | 0.4 |
| 20 | SA | 30 | 3.3 | 0.5 |
| 22 | TAP | 29 | 1.4 | 0.52 |
| 22 | TEAS-TAP | 29 | 1.18 | 0.42 |
| 22 | SA | 29 | 1.83 | 0.55 |

Abbreviations: id, study identification number; t, treatment group; n, sample size; mean, mean outcome value; sd, standard deviation.

**Table S6** Extracted data for Short-term VAS pain scores during movement

| id | t | n | mean | sd |
|---|---|---|---|---|
| 2 | EA | 30 | 1.2 | 1.5 |
| 2 | SA | 31 | 5.2 | 2.7 |
| 3 | TAP | 30 | 2.7 | 1.2 |
| 3 | SA | 31 | 4.3 | 0.8 |
| 6 | TAP | 29 | 2.5 | 2.963 |
| 6 | EA | 27 | 3.5 | 0.741 |
| 6 | SA | 26 | 4 | 0.741 |
| 17 | ITPB | 31 | 3.2 | 1 |
| 17 | TAP | 31 | 3.5 | 1.2 |
| 18 | EspB | 34 | 2 | 0.8848 |
| 18 | EA | 34 | 2 | 0.7348 |
| 18 | SA | 34 | 3 | 1.0497 |
| 19 | EOI | 30 | 5.33 | 1.09 |
| 19 | TAP | 30 | 5.23 | 0.9 |

Abbreviations: id, study identification number; t, treatment group; n, sample size; mean, mean outcome value; sd, standard deviation.

**Table S7** Extracted data for Long-term VAS pain scores during movement

| id | t | n | mean | sd |
|---|---|---|---|---|
| 2 | EA | 30 | 2.4 | 1.6 |
| 2 | SA | 31 | 4.2 | 1.8 |
| 3 | TAP | 30 | 4.1 | 0.6 |
| 3 | SA | 31 | 4.6 | 0.8 |
| 6 | TAP | 29 | 3.5 | 0.741 |
| 6 | EA | 27 | 4 | 0.37 |
| 6 | SA | 26 | 4.5 | 0.741 |
| 11 | EA | 30 | 5 | 1.504 |
| 11 | SA | 30 | 6.02 | 2.21 |
| 17 | ITPB | 31 | 2.4 | 0.8 |
| 17 | TAP | 31 | 3.4 | 0.7 |
| 18 | EspB | 34 | 4 | 0.8848 |
| 18 | EA | 34 | 4 | 1.1847 |
| 18 | SA | 34 | 5 | 0.9448 |
| 19 | EOI | 30 | 2.93 | 1.2 |
| 19 | TAP | 30 | 3.57 | 0.5 |

| | | | | |
|---|---|---|---|---|
| 22 | TAP | 29 | 2.85 | 0.53 |
| 22 | TEAS-TAP | 29 | 2.67 | 0.55 |
| 22 | SA | 29 | 3.6 | 0.57 |

Abbreviations: id, study identification number; t, treatment group; n, sample size; mean, mean outcome value; sd, standard deviation.

**Table S8** Extracted data for Postoperative opioid consumption during movement

| id | t | n | mean | sd |
|---|---|---|---|---|
| 3 | TAP | 30 | 46.6 | 8.4 |
| 3 | SA | 31 | 66.8 | 10.9 |
| 6 | TAP | 29 | 55 | 19 |
| 6 | EA | 27 | 32 | 23 |
| 6 | SA | 26 | 79 | 22 |
| 15 | QLB | 27 | 91 | 7.7 |
| 15 | SA | 26 | 100.5 | 6.6 |
| 19 | EOI | 30 | 226.83 | 32.68 |
| 19 | TAP | 30 | 247.67 | 34.88 |
| 23 | ESPB | 28 | 901 | 291.5 |
| 23 | SA | 30 | 956.9 | 294.6 |
| 17 | TAP | 31 | 27.8 | 5.7 |
| 17 | SA | 31 | 31.2 | 4.4 |

Abbreviations: id, study identification number; t, treatment group; n, sample size; mean, mean outcome value; sd, standard deviation.

**Table S9** Extracted data for $CD4^+/CD8^+$ T-cell ratio

| id | t | n | mean | sd |
|---|---|---|---|---|
| 4 | EA | 20 | 1.2 | 0.2 |
| 4 | SA | 20 | 1 | 0.2 |
| 7 | EA | 100 | 1.61 | 0.24 |
| 7 | SA | 100 | 1.52 | 0.33 |
| 10 | EA | 40 | 1.47 | 0.21 |
| 10 | SA | 40 | 1.43 | 0.35 |
| 15 | QLB | 27 | 1.289 | 0.211 |

| | | | | |
|---|---|---|---|---|
| 15 | SA | 26 | 1.106 | 0.191 |
| 16 | EA | 12 | 1.1 | 0.1 |
| 16 | SA | 9 | 0.7 | 0.1 |
| 22 | TAP | 29 | 0.85 | 0.71 |
| 22 | TEAS-TAP | 29 | 1.12 | 0.29 |
| 22 | SA | 29 | 0.83 | 0.2 |

Abbreviations: id, study identification number; t, treatment group; n, sample size; mean, mean outcome value; sd, standard deviation.

**Table S10** Baseline characteristics of the included studies

| No. | First Author (Year) | Regional anesthesia technique | RA-Timing | RA-Medication | Postoperative analgesia | PONV-Prophylaxis | Adjuvants |
|---|---|---|---|---|---|---|---|
| 1 | Kai Li 2015 | Ultrasound-guided bilateral subcostal transversus abdominis plane (TAP) block performed using an in-plane technique with a 5–10 MHz probe and a 20-gauge needle. A continuous subcostal plane was established through two sequential needle insertions: the initial injection was | Performed before skin incision; surgery commenced Approximately 30 min after block. | A total of 40 mL of 0.375% ropivacaine was administered, delivered as two separate 20-mL injections to establish a continuous fascial plane. | PCIA | No routine prophylaxis reported; nausea/vomiting recorded as adverse events; PCIA pump connected (analgesic regimen, | Induction: midazolam 0.02 mg/kg + cisatracurium 0.15 mg/kg + sufentanil 0.3 μg/kg + propofol 2.0–2.5 mg/kg; Maintenance: sevoflurane (BIS 45–60); Intraoperative rescue: sufentanil 5–10 μg for HR/BP >20% above baseline; nicardipine/esmolol as needed; |

| | | | | | | | |
|---|---|---|---|---|---|---|---|
| | | delivered adjacent to the lateral border of the rectus sheath, followed by a second injection at the junction of the costal margin and the anterior axillary line to ensure adequate longitudinal spread within the transversus abdominis plane.<br>The same ultrasound-guided bilateral subcostal TAP block technique as used in the R group was performed; however, an equivalent volume of normal saline was administered as placebo. | | | | dose, background infusion, and lockout interval not reported). | Possible adjunct: parecoxib 40 mg, 30 min before surgery end. |
| 2 | Liu Fei 2017 | Thoracic epidural anesthesia (T8–9): catheter placement ~30 min before skin incision;<br>Test dose: 3 mL of 2% lidocaine; | Approximately 30 min before skin | Maintenance: continuous epidural infusion of 0.2% ropivacaine with | PCEA | Tropisetron 40 mg every 12 h for 3 days. | fentanyl, sufentanil, cisatracurium, propofol, sevoflurane |

| | | | | | | | |
|---|---|---|---|---|---|---|---|
| | | Loading dose: 4–6 mL of 1% ropivacaine; Maintenance: continuous epidural infusion of 0.2% ropivacaine combined with sufentanil (0.1–0.2 µg/kg). | incision | sufentanil (0.33 µg/mL). | | | |
| | | No preoperative epidural analgesia. | | | PCIA | | |
| 3 | Ruizhu Liu 2019 | Ultrasound-guided bilateral subcostal transversus abdominis plane (TAP) block performed after induction of general anesthesia and ~30 min before skin incision; 0.375% ropivacaine, total volume 40 mL (bilateral). | Approximately 30 min before skin incision | 0.375% ropivacaine, 40 mL (bilateral; multi-point injection to achieve continuous fascial spread). | PCIA | NR | Intraoperative agents: opioids (sufentanil), inhalational anesthetics (sevoflurane, nitrous oxide), neuromuscular blocking agents (cisatracurium). |
| | | No regional anesthesia performed; standard general anesthesia. | | | PCIA | | |
| 4 | Liping Wang | Thoracic epidural catheterization at T8–10 before induction of general | Pre-incision | Intraoperative: 0.5% ropivacaine 5–7 mL | PCEA | No fixed prophylactic | General anesthetic agents: propofol, cisatracurium, remifentanil, sufentanil. |

| | | | | | | | |
|---|---|---|---|---|---|---|---|
| | 2019 | anesthesia; loading dose: 0.5% ropivacaine 5–7 mL (dose adjusted to height/weight); maintenance: continuous epidural infusion of ropivacaine. | | loading dose, followed by continuous epidural infusion. | | regimen; PONV incidence recorded. | |
| | | No preoperative epidural analgesia. | | | PCIA | | |
| 5 | Weixiong H 2017 | Intervention group: intravenous access established; 500 mL balanced crystalloid preload; epidural catheterization at T8; 0.4% bupivacaine administered epidurally; general anesthesia initiated after sensory block reached T10. | Pre-incision | 0.4% ropivacaine, epidural administration (sensory block to T10). | NR | NR | General anesthetic agents: atropine, propofol, sevoflurane. |
| | | No regional block; standard general anesthesia. | | | | | |
| 6 | Yiquan Wu 2013 | TAP group: ultrasound-guided bilateral subcostal transversus abdominis plane | Pre-incision | TAP: 0.375% ropivacaine 20 mL/side. | PCIA | NR | Induction: propofol 1.5–2.0 mg/kg, sufentanil 0.3 μg/kg, rocuronium 0.9 |

| | | | | | | | |
|---|---|---|---|---|---|---|---|
| | | (TAP) block after induction of general anesthesia; 0.375% ropivacaine 20 mL per side.<br>EA group: thoracic epidural catheterization at T8–9 before induction of general anesthesia; test dose: 1.5% lidocaine 4 mL; pre-induction loading: 0.25% ropivacaine 8 mL; maintenance: continuous epidural infusion of 0.25% ropivacaine at 5 mL/h intraoperatively; general anesthesia induced and maintained per protocol. | | EA: pre-induction epidural loading with 0.25% ropivacaine 8 mL; intraoperative infusion of 0.25% ropivacaine 5 mL/h. | PCIA＋PCEA | | mg/kg;<br>Maintenance: propofol/remifentanil/sevoflurane;<br>TAP and GA groups: supplemental sufentanil 0.1 μg/kg every 1.5 h intraoperatively;<br>EA group: additional epidural test dose with 1.5% lidocaine 4 mL. |
| | | No regional block. | | | PCIA | | |
| 7 | Li Xin 2019 | Thoracic epidural (T7–T8): initial epidural injection of 2% lidocaine 3 mL; sensory level assessed after 5 | Pre-incision | Epidural: 2% lidocaine 3 mL test dose; additional 2% lidocaine | PCEA | NR | Preoperative medication: phenobarbital sodium 0.1 g + atropine 0.5 mg (IM);<br>Induction: vecuronium 0.12 mg/kg + |

| | | | | | | | |
|---|---|---|---|---|---|---|---|
| | | min; additional 2% lidocaine 4–6 mL administered after tracheal intubation; maintenance: continuous epidural infusion of 2% lidocaine 3–5 mL/h until end of surgery; sensory block maintained at T4–T10. | | 4–6 mL; continuous epidural infusion of 2% lidocaine 3–5 mL/h until end of surgery. | | | fentanyl 2–3 μg/kg + propofol 2 mg/kg; Maintenance: propofol 2–4 mg/kg/h (continuous IV infusion) + inhalational anesthetic (enflurane* 1–2%, end-tidal 0.4–0.9 MAC) + pancuronium for neuromuscular blockade; |
| | | No preoperative epidural analgesia. | | | PCIA | NR | Intraoperative supplementation: fentanyl 3 μg/kg and vecuronium 0.05–0.08 mg/kg. |
| 8 | Susie Yoon 2022 | Ultrasound-guided bilateral subcostal transversus abdominis plane block (TAPB) performed at the end of surgery under general anesthesia; 0.375% ropivacaine 15 mL/side. | At the end of surgery (after skin closure), under general anesthesia. | 0.375% ropivacaine 15 mL/side (total 30 mL). | PCIA | PONV prophylaxis (induction): palonosetron 0.075 mg for all patients; dexamethasone 5 mg for Apfel score ≥2. | General anesthesia: induction with propofol/remifentanil/rocuronium; maintenance with desflurane + remifentanil + rocuronium; intraoperative nefopam 20 mg IV infusion (30 min); IV-PCA loading dose: fentanyl 50 μg after skin closure. |
| | | No TAPB. | | | PCIA | | |

| | | | | | | | |
|---|---|---|---|---|---|---|---|
| 9 | Armeana Olimpia Zgâia 2017 | Epidural catheterization pre-induction (T8–L2 based on incision level); test dose: 1% lidocaine + epinephrine 3 mL; epidural injection of 0.3% ropivacaine 5–7 mL 30 min before end of surgery. | Pre-incision | Epidural catheter test dose: 1% lidocaine + epinephrine 3 mL; Epidural injection 30 min before surgery end: 0.3% ropivacaine 5–7 mL; Postoperative epidural infusion: Day 1: 0.3% ropivacaine + fentanyl 2 µg/mL; Day 2: 0.2% ropivacaine + fentanyl 2 µg/mL. | PCEA | No fixed prophylactic regimen reported; nausea/vomiting incidence recorded. | 30 min before surgery end: PCEA group: epidural 0.3% ropivacaine 5–7 mL; IVMO group: ketorolac 100 mg IV + morphine 5 mg IV. |
| | | No epidural analgesia; 30 min before end of surgery: ketorolac 100 mg IV + morphine 5 mg IV. | | | PCIA | | |
| 10 | Min Zhou 2021 | Epidural catheterization at T8–T9; 1.5% lidocaine injection; followed by general anesthesia induction/maintenance for surgery. | Pre-incision | 1.5% lidocaine | NR | NR | Both groups received fentanyl (supplemented as needed). |

| | | | | | | | |
|---|---|---|---|---|---|---|---|
| 11 | Zhenxin Zhu 2013 | No epidural procedure. Epidural catheterization at T8–T9 pre-induction; postoperative continuous epidural infusion: 0.05% bupivacaine + morphine 100 µg/mL, base rate 4 mL/h for 48 h, with patient-controlled bolus of 4 mL/each, lockout 30 min; both groups can use pethidine for breakthrough pain management. | Pre-incision | Epidural: 0.05% bupivacaine + morphine 100 µg/mL; base rate 4 mL/h for 48 h; PCEA bolus 4 mL per dose, lockout 30 min. | PCIA | NR | Epidural mixture with morphine (as adjunct for analgesia); breakthrough pain management: pethidine. |
| 12 | T. Kawasaki 2007 | 术后使用静脉吗啡 PCIA：Epidural catheterization at T6–T9 (inserted 3–5 cm cephalad); pre-incision: 2% mepivacaine 8–12 mL, sensory block confirmed at T4–S5; intraoperative: continuous epidural infusion of 2% mepivacaine | Pre-incision | Induction: 2% mepivacaine 8–12 mL initial dose; Intraoperative: continuous 2% mepivacaine 5–7 mL/h; | PCIA PCEA | NR | Broteplazol 0.25 mg PO, administered 2 h preoperatively. |

| | | | | | | | |
|---|---|---|---|---|---|---|---|
| | | 5–7 mL/h, combined with general anesthesia (propofol + fentanyl + vecuronium for induction). | | Postoperative: 1% mepivacaine 5 mL/h + fentanyl 20 μg/h;<br>Rescue: VAS >4, additional 1% mepivacaine 5 mL;<br>POD5: extubation. | | | |
| | | General anesthesia only; no epidural block. | | | PCEA | | |
| 13 | Liu Kun 2014 | Epidural catheterization at T9–T10 pre-induction; test dose: 1% lidocaine 10 mL; sensory block confirmed; pre-incision loading dose: 0.375% ropivacaine 5 mL; intraoperative maintenance: continuous epidural local anesthesia, sensory block level approximately T4–L2. | Pre-incision | Test dose: 1% lidocaine 10 mL;<br>Loading dose: 0.375% ropivacaine 5 mL;<br>Intraoperative: continuous epidural infusion. | PCEA | NR | Sedation/induction: propofol, fentanyl, vecuronium;<br>Maintenance: propofol + sufentanil. |
| | | No regional nerve block; standard general anesthesia only. | | | PCIA | | |

| | | | | | | | |
|---|---|---|---|---|---|---|---|
| 14 | Seongwook Hong 2019 | After induction of general anesthesia and before skin incision, ultrasound-guided bilateral rectus sheath block performed; 0.375% ropivacaine 40 mL, injected in 3–4 points within the rectus sheath; local anesthetic confirmed to spread evenly within the incision area. | Pre-incision | Bilateral rectus sheath injection: 0.375% ropivacaine 40 mL. | PCIA | PCA includes ramosetron (5-HT3 receptor antagonist) as routine antiemetic. | General anesthesia: propofol + remifentanil (TCI); Neuromuscular blockade: rocuronium; Circulatory regulation: phenylephrine, nicardipine, atropine, esmolol (as needed). |
| | | At RSB site, 40 mL normal saline injected as placebo. | | | PCIA | | |
| 15 | Fuchun Wang 2025 | Pre-induction, lateral position: ultrasound-guided bilateral QLB III at L2 transverse process level; needle inserted between quadratus lumborum and psoas fascia; 0.25% ropivacaine 30 mL per side; block confirmed via needle test after ~30 min, followed by | Performed approximately 30 min before skin incision. | Injection per side: 0.25% ropivacaine 30 mL; Injection plane: between quadratus lumborum and psoas fascia at L2 transverse | PCIA | Palonosetron 0.25 mg (added to PCA, 5-HT3 receptor antagonist). | Induction: sufentanil, propofol, cisatracurium; Maintenance: propofol TCI + remifentanil continuous infusion; Circulatory support: deoxymetanephrine, esmolol, as needed. |

| | | | | | | | |
|---|---|---|---|---|---|---|---|
| | | general anesthesia induction. | | process level. | | | |
| | | Control group: no regional nerve block; standard general anesthesia induction and maintenance. | | | PCIA | | |
| 16 | T Hashimoto 1995 | Epidural catheterization at T6–T9 pre-induction, 3–5 cm cephalad advancement; pre-incision: 1.5% lidocaine 7–10 mL, sensory block confirmed at T4–S5; maintenance: continuous epidural infusion of 1.5% lidocaine 5–7 mL/h. | Pre-incision | Test/loading dose: 1.5% lidocaine 7–10 mL; Maintenance: continuous epidural infusion of 1.5% lidocaine 5–7 mL/h. | PCEA | NR | Preoperative: hydroxyzine 1 mg/kg + atropine 0.1 mg/kg IM; Induction: thiopental sodium 5 mg/kg, succinylcholine 1 mg/kg; Maintenance: $N_2O/O_2$, pancuronium for neuromuscular blockade. |
| | | No regional nerve block performed. | | | PCIA | | |
| 17 | Chen Q 2025 | After induction of general anesthesia and before skin incision, ultrasound-guided bilateral two-point thoracic interfascial plane block (ITPB) at T6/7 and T9/10; needle | Pre-incision | 0.3% ropivacaine 15 mL per side at T6/7 and T9/10 (two-point injection, total 60 mL). | PCIA | Palonosetron 75 µg IV bolus at end of surgery; added to PCA pump for maintenance: 0.15 | Preoperative: midazolam 1 mg + fentanyl 50 µg (pre-block sedation and analgesia); Intraoperative: parecoxib 40 mg (NSAID, 30 min before surgery end); |

| | | | | | | | |
|---|---|---|---|---|---|---|---|
| | | inserted between the posterior edge of the transverse process and pleura at each level; 0.3% ropivacaine 15 mL per side (total 60 mL) for extensive paravertebral spread, blocking somatic and sympathetic afferent nerves. Standard general anesthesia subsequently performed. Control group: Post-induction, pre-incision: ultrasound-guided bilateral subcostal TAPB; 0.3% ropivacaine 30 mL/side (total 60 mL); all other anesthesia and analgesia identical to ITPB group. | | Subcostal plane injection: 0.3% ropivacaine 30 mL per side (single injection, total 60 mL). | | mg. | Anesthesia maintenance: propofol, remifentanil, sevoflurane; Neuromuscular reversal: sugammadex 3 mg/kg. |
| 18 | Wang C 2025 | EspB: ultrasound-guided bilateral erector spinae plane block at T8 pre-induction; 0.375% ropivacaine 15 | Pre-incision | At T8 level, bilateral injection of 0.375% ropivacaine 15 mL/side. | PCIA | Routine postoperative as-needed use of | Induction: sufentanil 0.4 μg/kg + etomidate 0.2–0.3 mg/kg + rocuronium 0.6 mg/kg; |

| | | | | | |
|---|---|---|---|---|---|
| | mL per side (total 30 mL); sensory block covering T4–T10; followed by standard general anesthesia induction. EA group: epidural catheterization at T7–8 or T8–9 pre-induction; test dose: lidocaine 3 mL, followed by 0.375% ropivacaine 6–8 mL for block establishment; postoperative maintenance: continuous epidural infusion of 0.375% ropivacaine at approximately 5 mL/h; general anesthesia induction and maintenance identical to ESP group. No effective regional block; subcutaneous injection of 2% lidocaine 1 mL at T8–9 as placebo epidural. | EA group: test dose: lidocaine 3 mL; Loading dose: 0.375% ropivacaine 6–8 mL; Postoperative maintenance: continuous epidural infusion of 0.375% ropivacaine (approximately 5 mL/h). | | ondansetron 4 mg. | Maintenance: propofol 1–3 mg/kg/h + remifentanil 0.05–0.5 µg/kg/min + sevoflurane 1–2%; Circulatory support: ephedrine 5 mg, atropine 0.02 mg/kg (as needed). |

| | | | | | | | |
|---|---|---|---|---|---|---|---|
| 19 | Fang Tang 2024 | Pre-induction: ultrasound-guided bilateral external oblique intercostal block at the 6th rib level, between external oblique and intercostal muscles; each side injected with a mixture of 10 mL 1% ropivacaine, 5 mL 2% lidocaine, and 5 mL normal saline (total 20 mL/side, 40 mL total), targeting the anterior lateral cutaneous branches of T6/7–T10/11 intercostal nerves; followed by standard general anesthesia induction.<br>Control group: Pre-induction, ultrasound-guided bilateral subcostal transversus abdominis plane (TAP) block; same volume and composition of local anesthetic mixture injected per | Pre-incision | Both groups received the same local anesthetic mixture per side:<br>1% ropivacaine 10 mL, 2% lidocaine 5 mL, normal saline 5 mL;<br>Total per side: 20 mL; total for both sides: 40 mL. | PCIA | NR | Induction and maintenance of general anesthesia: propofol, opioids, neuromuscular blocking agents (routine);<br>Rescue analgesia: flurbiprofen axetil (NSAID). |

| | | | | | | | |
|---|---|---|---|---|---|---|---|
| | | side (total 40 mL); all other anesthesia induction, maintenance, and postoperative PCA analgesia identical to EOI group, with the only difference being the regional block plane. | | | | | |
| 20 | Zhongping Cheng 2024 | Pre-induction: ultrasound-guided bilateral low-level serratus anterior plane block at the 8th rib along the midaxillary line, using in-plane needle insertion technique; needle positioned at the superficial fascia between the serratus anterior muscle; 0.375% ropivacaine 25 mL per side; sensory block confirmed at T5–T11 after 30 min; followed by standard general anesthesia induction. | Pre-incision | Injection at the superficial layer of the serratus anterior muscle: 0.375% ropivacaine 25 mL per side (total 50 mL bilateral). | PCIA | NR | Induction: midazolam 0.02 mg/kg, sufentanil 0.3 μg/kg, etomidate 0.2–0.3 mg/kg, cisatracurium 0.15 mg/kg; Maintenance: propofol 3–6 mg/(kg·h), remifentanil 0.1–0.3 μg/(kg·min). |
| | | Control group: No effective regional | | | PCIA | | |

| | | | | | | | |
|---|---|---|---|---|---|---|---|
| | | block; equal volume of normal saline injected at the same site as placebo block. | | | | | |
| 21 | Shang Y 2022 | Pre-induction, lateral position: ultrasound-guided bilateral thoracic paravertebral block at T8 level; needle inserted into the paravertebral space with pleural sliding confirmed; 0.375% ropivacaine 20 mL per side (total 40 mL); sensory block level confirmed with needle test after 20 min; followed by total intravenous anesthesia (TIVA).<br><br>Control group: No regional nerve block; standard total intravenous anesthesia (TIVA) induction and | Pre-incision | Single-shot bilateral T8 paravertebral block: 0.375% ropivacaine 20 mL per side (total 40 mL); sustained effect during surgery. | PCIA | Tropisetron 10 mg (added to PCA pump). | Induction: midazolam, etomidate, sufentanil, cisatracurium; Maintenance: propofol + remifentanil (BIS 40–60); Postoperative: parecoxib 40 mg; Circulatory support: deoxymetanephrine, ephedrine (as needed). |

| | | | | | | | |
|---|---|---|---|---|---|---|---|
| 22 | Ruyi Xing 2022 | maintenance, identical to PVB group. Control group: No true TEAS; electrodes placed on acupoints without electrical stimulation; Post-induction: bilateral TAPB with 0.375% ropivacaine 20 mL per side. Pre-induction: Electrodes placed at bilateral Hegu (LI4), Neiguan (PC6), and Zusanli (ST36) acupoints 30 min before anesthesia; transcutaneous electrical acupoint stimulation at 2/100 Hz, with intensity adjusted to produce mild local muscle twitching, tolerated by the patient, and continued until the end of surgery. Control group: General anesthesia only; no effective TEAS or TAPB. | Pre-incision | Bilateral injection between internal oblique and transversus abdominis muscles: 0.375% ropivacaine 20 mL per side (total 40 mL). | PCIA | Prophylactic medication: azasetron 10 mg at the end of surgery; Antiemetic strategy in PCA: included in the PCA regimen. | Induction: midazolam 0.04 mg/kg, etomidate 0.3 mg/kg, sufentanil 0.3 μg/kg, rocuronium 1 mg/kg; Maintenance: propofol TCI + remifentanil TCI (BIS 40–60); Postoperative: flurbiprofen axetil 50 mg (analgesia) + azasetron 10 mg (antiemetic). |

| | | | | | | | |
|---|---|---|---|---|---|---|---|
| 23 | Jeong H 2022 | Pre-induction, prone position: ultrasound-guided bilateral erector spinae plane block at T7 level; in-plane needle insertion technique, with the needle tip positioned between the erector spinae muscle and the transverse process; 0.375% ropivacaine 30 mL per side (total 60 mL); sensory block confirmed at the back and midaxillary line 15 min after injection, followed by general anesthesia induction. | Pre-incision | 0.375% ropivacaine 30 mL per side. | PCIA | Ramosetron 0.3 mg for as-needed PONV treatment (PACU and ward). | Induction: propofol 2–3 mg/kg, rocuronium 0.8 mg/kg; Maintenance: sevoflurane (BIS 40–60); Postoperative: hydromorphone 0.01 mg/kg; Neuromuscular reversal: neostigmine + glycopyrrolate. |
| | | Control group: No regional nerve block; standard general anesthesia induction and maintenance, identical to experimental group. | | | PCIA | Ramosetron 0.4 mg for as-needed PONV treatment (PACU and ward). | |
| | | Control group: No regional nerve | | | PCIA | | |

| | |
|---|---|
| | block performed. |

Abbreviations:

RA-Timing, timing of regional anesthesia administration;

RA-Medication, regional anesthesia regimen;

PCIA, patient-controlled intravenous analgesia;

PCEA, patient-controlled epidural analgesia.

**Table S11** P values of the global inconsistency test for closed loops for each outcome measure

| Outcome Measure | p-value |
|---|---|
| Short-term VAS pain scores at rest | 0.8362 |
| Long-term VAS pain scores at rest | 0.4321 |
| Long-term VAS pain scores during movement | 0.2043 |
| Postoperative opioid consumption | 0.8479 |

**Table S12** P values of local inconsistency assessed by the node-splitting method for Short-term VAS pain scores at rest

| Side | Direct | | Indirect | | Difference | | | tau |
|---|---|---|---|---|---|---|---|---|
| | Coef. | Std. Err. | Coef. | Std. Err. | Coef. | Std. Err. | P>z | |
| EA vs EspB | 1.39e-09 | 1.167252 | -0.462551<br>2 | 2.397278 | 0.4625512 | 2.663646 | 0.862 | 1.146205 |
| EA vs SA | 0.8889421 | 0.5504529 | -0.338843<br>9 | 2.223249 | 1.227786 | 2.286642 | 0.591 | 1.072374 |
| EA vs TAP | -1.530089 | 1.085044 | 0.049147 | 0.9164676 | -1.579236 | 1.41547 | 0.265 | 0.9974871 |
| EOI vs TAP | 0.1600001 | 1.03936 | -1.212886 | 53.17516 | 1.372886 | 53.18519 | 0.979 | 1.016361 |
| EspB vs SA | 1 | 1.164357 | 0.5374488 | 2.401497 | 0.4625512 | 2.663646 | 0.862 | 1.146205 |
| ITPB vs TAP | 0.8000001 | 1.039109 | -1.213313 | 53.80731 | 2.013313 | 53.81745 | 0.97 | 1.016353 |
| SA vs SAPB | -1.6 | 1.025246 | -1.627919 | 25.3803 | 0.0279189 | 25.40163 | 0.999 | 1.017086 |
| SA vs TAP | -1.256001 | 0.592638 | -3.747917 | 2.103171 | 2.491916 | 2.163521 | 0.249 | 0.9912853 |

**Table S13** P values of local inconsistency assessed by the node-splitting method for Long-term VAS pain scores at rest

| Side | Direct | | Indirect | | Difference | |
|---|---|---|---|---|---|---|
| | Coef. | Std. Err. | Coef. | Std. Err. | Coef. | Std. Err. |
| EA vs EspB | -1.56E-12 | 0.6801633 | 0.7362458 | 1.311269 | -0.7362458 | 1.484499 |

| | | | | | | |
|---|---|---|---|---|---|---|
| EA vs SA | 0.8440853 | 0.2847463 | -0.0874667 | 1.31087 | 0.931552 | 1.338322 |
| EA vs TAP | -0.9899267 | 0.6358782 | -0.0597457 | 0.4603973 | -0.930181 | 0.7859632 |
| EOI vs TAP | 0.4399999 | 0.6124175 | -0.7591791 | 36.07273 | 1.199179 | 36.07822 |
| EspB vs SA | 0.5 | 0.6664109 | 1.236246 | 1.332285 | -0.7362459 | 1.484499 |
| ITPB vs TAP | 1.1 | 0.6160271 | -0.7592817 | 41.12967 | 1.859282 | 41.13419 |
| SA vs SAPB | 0.2 | 0.6048019 | -1.60936 | 28.48468 | 1.80936 | 28.49121 |
| SA vs TAP | -1.096892 | 0.3003957 | -2.680728 | 1.24186 | 1.583836 | 1.277866 |
| SA vs TEAS-TAP | -0.6500001 | 0.5454372 | -2.655384 | 1.135006 | 2.005383 | 1.26271 |
| TAP vs TEAS-TAP | -0.22 | 0.5444317 | 1.78577 | 1.13669 | -2.00577 | 1.262914 |

**Table S14** P values of local inconsistency assessed by the node-splitting method for Long-term VAS pain scores during movement

| Side | Direct | | Indirect | | Difference | | | tau |
|---|---|---|---|---|---|---|---|---|
| | Coef. | Std. Err. | Coef. | Std. Err. | Coef. | Std. Err. | P>z | |
| EA vs EspB | -1.68E-09 | 0.5074549 | -0.2607894 | 1.086301 | 0.2607894 | 1.214112 | 0.83 | 0.4395509 |
| EA vs SA | 0.9487416 | 0.2449533 | -0.2882963 | 0.8294805 | 1.237038 | 0.8679755 | 0.154 | 0.327387 |
| EA vs TAP | -0.5272588 | 0.1566641 | 0.4711953 | 0.2476834 | -0.9984542 | 0.2939329 | 0.07 | 0.0358846 |
| EOI vs TAP | 0.6399999 | 0.4495054 | 0.0516945 | 53.76818 | 0.5883054 | 53.76932 | 0.991 | 0.3817352 |
| EspB vs SA | 1 | 0.4924276 | 0.7392107 | 1.106855 | 0.2607893 | 1.214113 | 0.83 | 0.4395509 |
| ITPB vs TAP | 1 | 0.4268195 | 0.0516193 | 47.44165 | 0.9483807 | 47.44347 | 0.984 | 0.3817372 |
| SA vs TAP | -0.740697 | 0.1332866 | -2.399788 | 0.5819329 | 1.659091 | 0.5971368 | 0.15 | 0.1520551 |
| SA vs TEAS-TAP | -0.9299998 | 0.4847903 | -1.258062 | 1.073229 | 0.328062 | 1.178298 | 0.781 | 0.4619385 |
| TAP vs TEAS-TAP | -0.1799998 | 0.4832351 | 0.1474547 | 1.075493 | -0.3274545 | 1.178436 | 0.781 | 0.4619512 |

**Table S15** P values of local inconsistency assessed by the node-splitting method for Postoperative opioid consumption

| Side | Direct | | Indirect | | Difference | | | tau |
|---|---|---|---|---|---|---|---|---|

| | Coef. | Std. Err. | Coef. | Std. Err. | Coef. | Std. Err. | P>z | |
|---|---|---|---|---|---|---|---|---|
| EA vs SA | 2.181901 | 0.9784625 | 2.59132 | 2.133064 | -0.4094186 | 2.347642 | 0.862 | 0.9225391 |
| EA vs TAP | 1.067579 | 0.9651457 | 0.5940452 | 2.155902 | 0.4735336 | 2.351551 | 0.84 | 0.9233693 |
| EOI vs TAP | 0.6084456 | 0.6880895 | 1.996818 | 63.26909 | -1.388372 | 63.27283 | 0.982 | 0.6351775 |
| EspB vs SA | 0.1880993 | 0.6876311 | 4.515728 | 63.26744 | -4.327629 | 63.27114 | 0.945 | 0.6351825 |
| QLB vs SA | 1.30274 | 0.7046557 | 4.484182 | 63.27129 | -3.181441 | 63.27524 | 0.96 | 0.6351819 |
| SA vs TAP | -1.255842 | 0.4032571 | 0.7840401 | 36.52451 | -2.039882 | 36.52681 | 0.955 | 0.6351564 |

**Table S16** Treatment ranking based on SUCRA for Short-term VAS pain scores at rest

| Treatment | SUCRA | PrBest | MeanRank |
|---|---|---|---|
| ITPB | 79.3 | 45.7 | 2.2 |
| SAPB | 62.8 | 22.1 | 3.2 |
| EOI | 61.8 | 21.5 | 3.3 |
| TAP | 59.9 | 3 | 3.4 |
| EspB | 41.8 | 6.7 | 4.5 |
| EA | 37.5 | 1.1 | 4.8 |
| SA | 6.9 | 0 | 6.6 |

**Table S17** Treatment ranking based on SUCRA for Long-term VAS pain scores at rest

| Treatment | SUCRA | PrBest | MeanRank |
|---|---|---|---|
| ITPB | 94.3 | 73.2 | 1.4 |
| EOI | 78.4 | 21 | 2.5 |
| TAP | 64.2 | 0.5 | 3.5 |
| TEAS-TAP | 56.2 | 3.5 | 4.1 |
| EA | 45.8 | 0.3 | 4.8 |

| | | | |
|---|---|---|---|
| EspB | 39.7 | 1.5 | 5.2 |
| SA | 11.4 | 0 | 7.2 |
| SAPB | 9.9 | 0 | 7.3 |

**Table S18** Treatment ranking based on SUCRA for Short-term VAS pain scores during movement

| Treatment | SUCRA | PrBest | MeanRank |
|---|---|---|---|
| ITPB | 65.8 | 35.8 | 2.7 |
| TAP | 61.2 | 10 | 2.9 |
| EA | 58.6 | 14.2 | 3.1 |
| EOI | 56.4 | 24.3 | 3.2 |
| EspB | 47.7 | 15.8 | 3.6 |
| SA | 10.2 | 0 | 5.5 |

**Table S19** Treatment ranking based on SUCRA for Long-term VAS pain scores during movement

| Treatment | SUCRA | PrBest | MeanRank |
|---|---|---|---|
| ITPB | 92.3 | 66.9 | 1.5 |
| EOI | 78.4 | 26.6 | 2.3 |
| TEAS-TAP | 50.6 | 2.3 | 4 |
| EspB | 47.3 | 3.8 | 4.2 |
| EA | 42.2 | 0.4 | 4.5 |
| TAP | 39 | 0 | 4.7 |
| SA | 0.2 | 0 | 7 |

**Table S20** Treatment ranking based on SUCRA for Postoperative opioid consumption

| Treatment | SUCRA | PrBest | MeanRank |
|---|---|---|---|
| EA | 88.5 | 58.5 | 1.6 |
| EOI | 75.6 | 30.4 | 2.2 |
| QLB | 56.7 | 10 | 3.2 |
| TAP | 52.9 | 0.7 | 3.4 |
| ESPB | 17.5 | 0.4 | 5.1 |
| SA | 8.7 | 0 | 5.6 |

**Table S21** Treatment ranking based on SUCRA for $CD4^+/CD8^+$ T-cell ratio.

| Treatment | SUCRA | PrBest | MeanRank |
|---|---|---|---|
| TEAS-TAP | 81.8 | 54.6 | 1.7 |
| EA | 63.5 | 15.3 | 2.5 |
| QLB | 60.3 | 24.8 | 2.6 |
| TAP | 28.6 | 5.3 | 3.9 |
| SA | 15.8 | 0 | 4.4 |

**Table S22** Sensitivity Analysis Using Short-term VAS pain scores at rest as the Outcome Measure

| dropped_id | comparison | eff | lci | uci | connected |
|---|---|---|---|---|---|
| Kai Li 2015 | SA vs EA | -0.81536573 | -1.959296 | 0.3285643 | 1 |
| Kai Li 2015 | SA vs EOI | -1.5078074 | -4.319973 | 1.304358 | 1 |
| Kai Li 2015 | SA vs EspB | -0.90813356 | -2.993101 | 1.176834 | 1 |
| Kai Li 2015 | SA vs ITPB | -2.1475192 | -4.959347 | 0.6643083 | 1 |
| Kai Li 2015 | SA vs SAPB | -1.6 | -3.889249 | 0.6892486 | 1 |
| Kai Li 2015 | SA vs TAP | -1.3492104 | -2.948557 | 0.2501365 | 1 |
| Liu Fei 2017 | SA vs EA | -0.27424109 | -0.8709021 | 0.3224199 | 1 |
| Liu Fei 2017 | SA vs EOI | -1.4646458 | -2.740855 | -0.1884362 | 1 |
| Liu Fei 2017 | SA vs EspB | -0.64517542 | -1.646016 | 0.355665 | 1 |
| Liu Fei 2017 | SA vs ITPB | -2.1045705 | -3.380014 | -0.8291272 | 1 |
| Liu Fei 2017 | SA vs SAPB | -1.6 | -2.648083 | -0.5519165 | 1 |
| Liu Fei 2017 | SA vs TAP | -1.3049496 | -1.947565 | -0.6623338 | 1 |
| Ruizhu Liu 2019 | SA vs EA | -0.86490895 | -1.928409 | 0.1985909 | 1 |
| Ruizhu Liu 2019 | SA vs EOI | -1.9181374 | -4.524265 | 0.6879898 | 1 |
| Ruizhu Liu 2019 | SA vs EspB | -0.93283753 | -2.868968 | 1.003293 | 1 |
| Ruizhu Liu 2019 | SA vs ITPB | -2.5578919 | -5.163652 | 0.0478688 | 1 |
| Ruizhu Liu 2019 | SA vs SAPB | -1.6 | -3.721764 | 0.5217644 | 1 |
| Ruizhu Liu 2019 | SA vs TAP | -1.7596936 | -3.235574 | -0.2838136 | 1 |
| Weixiong H 2017 | SA vs EA | -1.0948261 | -2.294394 | 0.1047422 | 1 |
| Weixiong H 2017 | SA vs EOI | -1.6284715 | -4.058199 | 0.8012562 | 1 |
| Weixiong H 2017 | SA vs EspB | -1.0471345 | -2.97224 | 0.8779707 | 1 |
| Weixiong H 2017 | SA vs ITPB | -2.2682306 | -4.69756 | 0.1610983 | 1 |
| Weixiong H 2017 | SA vs SAPB | -1.6 | -3.684165 | 0.4841646 | 1 |
| Weixiong H 2017 | SA vs TAP | -1.4697373 | -2.67248 | -0.2669944 | 1 |
| Yiquan Wu 2013 | SA vs EA | -1.2041987 | -2.550041 | 0.1416442 | 1 |

| | | | | | |
|---|---|---|---|---|---|
| Yiquan Wu 2013 | SA vs EOI | -1.3043706 | -4.123326 | 1.514585 | 1 |
| Yiquan Wu 2013 | SA vs EspB | -1.1015984 | -3.21151 | 1.008313 | 1 |
| Yiquan Wu 2013 | SA vs ITPB | -1.9440808 | -4.762712 | 0.8745506 | 1 |
| Yiquan Wu 2013 | SA vs SAPB | -1.6 | -3.883442 | 0.6834425 | 1 |
| Yiquan Wu 2013 | SA vs TAP | -1.1455677 | -2.764961 | 0.4738258 | 1 |
| Chen Q 2025 | SA vs EA | -0.81524367 | -1.824863 | 0.1943754 | 1 |
| Chen Q 2025 | SA vs EOI | -1.5784275 | -3.937048 | 0.7801934 | 1 |
| Chen Q 2025 | SA vs EspB | -0.9081973 | -2.755785 | 0.9393904 | 1 |
| Chen Q 2025 | SA vs SAPB | -1.6 | -3.622966 | 0.4229662 | 1 |
| Chen Q 2025 | SA vs TAP | -1.419583 | -2.584454 | -0.254712 | 1 |
| Wang C 2025 | SA vs EA | -0.76983969 | -2.076121 | 0.5364415 | 1 |
| Wang C 2025 | SA vs EOI | -1.5737363 | -4.232339 | 1.084866 | 1 |
| Wang C 2025 | SA vs ITPB | -2.2134494 | -4.871694 | 0.444795 | 1 |
| Wang C 2025 | SA vs SAPB | -1.6 | -3.887582 | 0.6875821 | 1 |
| Wang C 2025 | SA vs TAP | -1.4152019 | -2.728092 | -0.1023114 | 1 |
| Fang Tang 2024 | SA vs EA | -0.81522559 | -1.824846 | 0.1943947 | 1 |
| Fang Tang 2024 | SA vs ESPB | -0.90818831 | -2.755781 | 0.9394044 | 1 |
| Fang Tang 2024 | SA vs ITPB | -2.2180607 | -4.576278 | 0.140157 | 1 |
| Fang Tang 2024 | SA vs SAPB | -1.6 | -3.622972 | 0.4229722 | 1 |
| Fang Tang 2024 | SA vs TAP | -1.4194446 | -2.584319 | -0.2545699 | 1 |
| Zhongping Cheng 2024 | SA vs EA | -0.8151022 | -1.824462 | 0.1942571 | 1 |
| Zhongping Cheng 2024 | SA vs EOI | -1.5775271 | -3.93542 | 0.7803653 | 1 |
| Zhongping Cheng 2024 | SA vs EspB | -0.90812728 | -2.755263 | 0.9390085 | 1 |
| Zhongping Cheng 2024 | SA vs ITPB | -2.2172987 | -4.574781 | 0.1401834 | 1 |
| Zhongping Cheng 2024 | SA vs SAPB | -1.4186814 | -2.582954 | -0.2544084 | 1 |

**Table S23** Sensitivity Analysis Using Long-term VAS pain scores at rest as the Outcome Measure

| dropped_id | comparison | eff | lci | uci | connected |
|---|---|---|---|---|---|
| Kai Li 2015 | SA vs EA | -0.77185417 | -1.204628 | -0.33908 | 1 |

| | | | | | |
|---|---|---|---|---|---|
| Kai Li 2015 | SA vs EOI | -1.3051166 | -2.389895 | -0.2203382 | 1 |
| Kai Li 2015 | SA vs EspB | -0.62670736 | -1.532249 | 0.2788342 | 1 |
| Kai Li 2015 | SA vs ITPB | -1.9651328 | -3.057817 | -0.8724491 | 1 |
| Kai Li 2015 | SA vs SAPB | 0.20000005 | -0.7204624 | 1.120462 | 1 |
| Kai Li 2015 | SA vs TAP | -0.86551813 | -1.407892 | -0.323144 | 1 |
| Kai Li 2015 | SA vs TEAS-TAP | -0.86882267 | -1.713003 | -0.0246427 | 1 |
| Liu Fei 2017 | SA vs EA | -0.74211206 | -1.351529 | -0.1326953 | 1 |
| Liu Fei 2017 | SA vs EOI | -1.6118292 | -3.016734 | -0.2069241 | 1 |
| Liu Fei 2017 | SA vs EspB | -0.61615277 | -1.797678 | 0.5653727 | 1 |
| Liu Fei 2017 | SA vs ITPB | -2.2718944 | -3.682968 | -0.8608204 | 1 |
| Liu Fei 2017 | SA vs SAPB | 0.20000005 | -1.046922 | 1.446922 | 1 |
| Liu Fei 2017 | SA vs TAP | -1.1727383 | -1.792591 | -0.5528856 | 1 |
| Liu Fei 2017 | SA vs TEAS-TAP | -1.0223698 | -2.147387 | 0.1026477 | 1 |
| Ruizhu Liu 2019 | SA vs EA | -0.82636703 | -1.344503 | -0.3082311 | 1 |
| Ruizhu Liu 2019 | SA vs EOI | -1.8111132 | -3.142567 | -0.4796591 | 1 |
| Ruizhu Liu 2019 | SA vs EspB | -0.65555623 | -1.74901 | 0.4378979 | 1 |
| Ruizhu Liu 2019 | SA vs ITPB | -2.4712003 | -3.809147 | -1.133254 | 1 |
| Ruizhu Liu 2019 | SA vs SAPB | 0.20000005 | -0.9491134 | 1.349113 | 1 |
| Ruizhu Liu 2019 | SA vs TAP | -1.3720033 | -2.018094 | -0.725913 | 1 |
| Ruizhu Liu 2019 | SA vs TEAS-TAP | -1.1224921 | -2.170506 | -0.0744779 | 1 |
| Liping Wang 2019 | SA vs EA | -0.78510184 | -1.358481 | -0.2117228 | 1 |
| Liping Wang 2019 | SA vs EOI | -1.6183623 | -3.002494 | -0.234231 | 1 |
| Liping Wang 2019 | SA vs EspB | -0.63662027 | -1.798999 | 0.5257583 | 1 |
| Liping Wang 2019 | SA vs ITPB | -2.2784273 | -3.668814 | -0.8880401 | 1 |
| Liping Wang 2019 | SA vs SAPB | 0.20000005 | -1.028165 | 1.428166 | 1 |
| Liping Wang 2019 | SA vs TAP | -1.1792497 | -1.789695 | -0.5688048 | 1 |
| Liping Wang 2019 | SA vs TEAS-TAP | -1.0256649 | -2.133796 | 0.0824661 | 1 |
| Weixiong H 2017 | SA vs EA | -0.69629733 | -1.304078 | -0.0885163 | 1 |
| Weixiong H 2017 | SA vs EOI | -1.6061914 | -2.977631 | -0.2347518 | 1 |

| | | | | | |
|---|---|---|---|---|---|
| Weixiong H 2017 | SA vs EspB | -0.59399494 | -1.751027 | 0.5630375 | 1 |
| Weixiong H 2017 | SA vs ITPB | -2.2662537 | -3.644004 | -0.8885035 | 1 |
| Weixiong H 2017 | SA vs SAPB | 0.20000005 | -1.016312 | 1.416312 | 1 |
| Weixiong H 2017 | SA vs TAP | -1.167055 | -1.772544 | -0.5615657 | 1 |
| Weixiong H 2017 | SA vs TEAS-TAP | -1.0195703 | -2.117165 | 0.0780245 | 1 |
| Yiquan Wu 2013 | SA vs EA | -0.97559372 | -1.596408 | -0.354779 | 1 |
| Yiquan Wu 2013 | SA vs EOI | -1.4804997 | -2.929907 | -0.0310925 | 1 |
| Yiquan Wu 2013 | SA vs EspB | -0.72805423 | -1.904556 | 0.4484471 | 1 |
| Yiquan Wu 2013 | SA vs ITPB | -2.1405432 | -3.595945 | -0.6851412 | 1 |
| Yiquan Wu 2013 | SA vs SAPB | 0.20000005 | -1.03888 | 1.43888 | 1 |
| Yiquan Wu 2013 | SA vs TAP | -1.041313 | -1.770262 | -0.3123643 | 1 |
| Yiquan Wu 2013 | SA vs TEAS-TAP | -0.95649068 | -2.091252 | 0.1782707 | 1 |
| Zhenxin Zhu 2013 | SA vs EA | -0.76569713 | -1.365615 | -0.1657797 | 1 |
| Zhenxin Zhu 2013 | SA vs EOI | -1.6151332 | -3.020983 | -0.2092836 | 1 |
| Zhenxin Zhu 2013 | SA vs EspB | -0.62747476 | -1.808607 | 0.5536578 | 1 |
| Zhenxin Zhu 2013 | SA vs ITPB | -2.275199 | -3.687214 | -0.8631845 | 1 |
| Zhenxin Zhu 2013 | SA vs SAPB | 0.20000005 | -1.047853 | 1.447853 | 1 |
| Zhenxin Zhu 2013 | SA vs TAP | -1.1760458 | -1.796168 | -0.5559235 | 1 |
| Zhenxin Zhu 2013 | SA vs TEAS-TAP | -1.0240265 | -2.149855 | 0.101802 | 1 |
| Chen Q 2025 | SA vs EA | -0.80477213 | -1.335821 | -0.2737234 | 1 |
| Chen Q 2025 | SA vs EOI | -1.6225939 | -2.960794 | -0.2843935 | 1 |
| Chen Q 2025 | SA vs EspB | -0.6456489 | -1.770106 | 0.4788079 | 1 |
| Chen Q 2025 | SA vs SAPB | 0.20000005 | -0.9863979 | 1.386398 | 1 |
| Chen Q 2025 | SA vs TAP | -1.1834274 | -1.773631 | -0.5932241 | 1 |
| Chen Q 2025 | SA vs TEAS-TAP | -1.0278332 | -2.098459 | 0.0427925 | 1 |
| Wang C 2025 | SA vs EA | -0.86882499 | -1.477669 | -0.2599815 | 1 |
| Wang C 2025 | SA vs EOI | -1.6297513 | -3.032415 | -0.2270874 | 1 |
| Wang C 2025 | SA vs ITPB | -2.2898193 | -3.698661 | -0.8809772 | 1 |
| Wang C 2025 | SA vs SAPB | 0.20000005 | -1.044883 | 1.444883 | 1 |

| | | | | | |
|---|---|---|---|---|---|
| Wang C 2025 | SA vs TAP | -1.1906693 | -1.809541 | -0.571798 | 1 |
| Wang C 2025 | SA vs TEAS-TAP | -1.0313628 | -2.15455 | 0.0918241 | 1 |
| Fang Tang 2024 | SA vs EA | -0.80477417 | -1.335833 | -0.2737152 | 1 |
| Fang Tang 2024 | SA vs EspB | -0.64565014 | -1.770129 | 0.4788288 | 1 |
| Fang Tang 2024 | SA vs ITPB | -2.2826755 | -3.627372 | -0.9379796 | 1 |
| Fang Tang 2024 | SA vs SAPB | 0.20000005 | -0.9864243 | 1.386424 | 1 |
| Fang Tang 2024 | SA vs TAP | -1.1834462 | -1.77368 | -0.593212 | 1 |
| Fang Tang 2024 | SA vs TEAS-TAP | -1.0278426 | -2.098495 | 0.0428093 | 1 |
| Zhongping Cheng 2024 | SA vs EA | -0.80473055 | -1.335749 | -0.273712 | 1 |
| Zhongping Cheng 2024 | SA vs EOI | -1.622225 | -2.960316 | -0.2841336 | 1 |
| Zhongping Cheng 2024 | SA vs EspB | -0.64562825 | -1.770019 | 0.4787627 | 1 |
| Zhongping Cheng 2024 | SA vs ITPB | -2.2822878 | -3.626838 | -0.9377372 | 1 |
| Zhongping Cheng 2024 | SA vs TAP | -1.1830582 | -1.773171 | -0.592945 | 1 |
| Zhongping Cheng 2024 | SA vs TEAS-TAP | -1.0276482 | -2.098197 | 0.0429 | 1 |
| Jeong H 2022 | SA vs EA | -0.83398944 | -1.320789 | -0.3471894 | 1 |
| Jeong H 2022 | SA vs EOI | -1.8717516 | -3.115547 | -0.6279562 | 1 |
| Jeong H 2022 | SA vs EspB | -0.65810116 | -1.681781 | 0.3655784 | 1 |
| Jeong H 2022 | SA vs ITPB | -2.5318377 | -3.782558 | -1.281117 | 1 |
| Jeong H 2022 | SA vs SAPB | 0.20000005 | -0.8650044 | 1.265005 | 1 |
| Jeong H 2022 | SA vs TAP | -1.4325525 | -2.04708 | -0.8180251 | 1 |

**Table S24** Sensitivity Analysis Using Short-term VAS pain scores during movement as the Outcome Measure

| dropped_id | comparison | eff | lci | uci | connected |
|---|---|---|---|---|---|
| Liu Fei 2017 | SA vs EA | -0.73043997 | -1.020973 | -0.4399068 | 1 |
| Liu Fei 2017 | SA vs EOI | -1.5029158 | -2.190536 | -0.8152954 | 1 |
| Liu Fei 2017 | SA vs EspB | -0.81908884 | -1.228257 | -0.4099211 | 1 |
| Liu Fei 2017 | SA vs ITPB | -1.9028907 | -2.62353 | -1.182252 | 1 |
| Liu Fei 2017 | SA vs TAP | -1.602958 | -2.068762 | -1.137154 | 1 |

| | | | | | |
|---|---|---|---|---|---|
| Ruizhu Liu 2019 | SA vs EA | -0.95025383 | -1.23299 | -0.6675177 | 1 |
| Ruizhu Liu 2019 | SA vs EOI | -1.6283121 | -2.844296 | -0.4123277 | 1 |
| Ruizhu Liu 2019 | SA vs EspB | -0.96661361 | -1.373314 | -0.5599135 | 1 |
| Ruizhu Liu 2019 | SA vs ITPB | -2.0282857 | -3.263233 | -0.7933385 | 1 |
| Ruizhu Liu 2019 | SA vs TAP | -1.7283578 | -2.834172 | -0.622544 | 1 |
| Yiquan Wu 2013 | SA vs EA | -1.4041428 | -1.804785 | -1.003501 | 1 |
| Yiquan Wu 2013 | SA vs EOI | -1.4997513 | -2.220529 | -0.7789738 | 1 |
| Yiquan Wu 2013 | SA vs EspB | -1.2712343 | -1.720342 | -0.8221269 | 1 |
| Yiquan Wu 2013 | SA vs ITPB | -1.8997262 | -2.652068 | -1.147385 | 1 |
| Yiquan Wu 2013 | SA vs TAP | -1.5997934 | -2.113285 | -1.086301 | 1 |
| Chen Q 2025 | SA vs EA | -0.94674221 | -1.227501 | -0.6659837 | 1 |
| Chen Q 2025 | SA vs EOI | -1.5228104 | -2.210404 | -0.8352168 | 1 |
| Chen Q 2025 | SA vs EspB | -0.96425684 | -1.370339 | -0.5581743 | 1 |
| Chen Q 2025 | SA vs TAP | -1.6228532 | -2.088618 | -1.157089 | 1 |
| Wang C 2025 | SA vs EA | -0.90738506 | -1.277614 | -0.5371559 | 1 |
| Wang C 2025 | SA vs EOI | -1.519123 | -2.207065 | -0.8311815 | 1 |
| Wang C 2025 | SA vs ITPB | -1.9190978 | -2.640043 | -1.198152 | 1 |
| Wang C 2025 | SA vs TAP | -1.6191657 | -2.085443 | -1.152888 | 1 |
| Fang Tang 2024 | SA vs EA | -0.94674244 | -1.227501 | -0.6659839 | 1 |
| Fang Tang 2024 | SA vs EspB | -0.96425699 | -1.37034 | -0.5581744 | 1 |
| Fang Tang 2024 | SA vs ITPB | -1.9227921 | -2.643407 | -1.202177 | 1 |
| Fang Tang 2024 | SA vs TAP | -1.6228602 | -2.088627 | -1.157094 | 1 |

**Table S25** Sensitivity Analysis Using Long-term VAS pain scores during movement as the Outcome Measure

| dropped_id | comparison | eff | lci | uci | connected |
|---|---|---|---|---|---|
| Liu Fei 2017 | SA vs EA | -0.52319703 | -0.7489567 | -0.2974373 | 1 |
| Liu Fei 2017 | SA vs EOI | -1.4228686 | -1.924982 | -0.9207555 | 1 |
| Liu Fei 2017 | SA vs EspB | -0.81463985 | -1.211889 | -0.4173903 | 1 |

| | | | | | |
|---|---|---|---|---|---|
| Liu Fei 2017 | SA vs ITPB | -1.7828454 | -2.202056 | -1.363635 | 1 |
| Liu Fei 2017 | SA vs TAP | -0.78288144 | -0.9718487 | -0.5939142 | 1 |
| Liu Fei 2017 | SA vs TEAS-TAP | -0.94763466 | -1.212775 | -0.6824942 | 1 |
| Ruizhu Liu 2019 | SA vs EA | -0.6532593 | -0.8762376 | -0.430281 | 1 |
| Ruizhu Liu 2019 | SA vs EOI | -1.5676595 | -2.083215 | -1.052104 | 1 |
| Ruizhu Liu 2019 | SA vs EspB | -0.86520238 | -1.262214 | -0.4681903 | 1 |
| Ruizhu Liu 2019 | SA vs ITPB | -1.9276339 | -2.362854 | -1.492414 | 1 |
| Ruizhu Liu 2019 | SA vs TAP | -0.927674 | -1.149909 | -0.705439 | 1 |
| Ruizhu Liu 2019 | SA vs TEAS-TAP | -1.0252892 | -1.297749 | -0.7528295 | 1 |
| Yiquan Wu 2013 | SA vs EA | -1.1770639 | -1.574893 | -0.7792346 | 1 |
| Yiquan Wu 2013 | SA vs EOI | -1.2923937 | -1.807508 | -0.7772797 | 1 |
| Yiquan Wu 2013 | SA vs EspB | -1.0688347 | -1.485996 | -0.6516732 | 1 |
| Yiquan Wu 2013 | SA vs ITPB | -1.6523727 | -2.08707 | -1.217675 | 1 |
| Yiquan Wu 2013 | SA vs TAP | -0.65240504 | -0.8736148 | -0.4311953 | 1 |
| Yiquan Wu 2013 | SA vs TEAS-TAP | -0.87765812 | -1.149878 | -0.6054386 | 1 |
| Zhenxin Zhu 2013 | SA vs EA | -0.58389233 | -0.8080676 | -0.3597171 | 1 |
| Zhenxin Zhu 2013 | SA vs EOI | -1.4403023 | -1.942357 | -0.9382478 | 1 |
| Zhenxin Zhu 2013 | SA vs EspB | -0.83823553 | -1.235349 | -0.4411216 | 1 |
| Zhenxin Zhu 2013 | SA vs ITPB | -1.8002788 | -2.219419 | -1.381139 | 1 |
| Zhenxin Zhu 2013 | SA vs TAP | -0.80031538 | -0.9891269 | -0.6115038 | 1 |
| Zhenxin Zhu 2013 | SA vs TEAS-TAP | -0.95698475 | -1.222093 | -0.6918762 | 1 |
| Chen Q 2025 | SA vs EA | -0.60660392 | -0.8248662 | -0.3883416 | 1 |
| Chen Q 2025 | SA vs EOI | -1.4468449 | -1.948685 | -0.9450048 | 1 |
| Chen Q 2025 | SA vs EspB | -0.84706481 | -1.243681 | -0.450449 | 1 |
| Chen Q 2025 | SA vs TAP | -0.80685804 | -0.9950987 | -0.6186174 | 1 |
| Chen Q 2025 | SA vs TEAS-TAP | -0.96049368 | -1.225485 | -0.6955019 | 1 |
| Wang C 2025 | SA vs EA | -0.51811098 | -0.759675 | -0.276547 | 1 |
| Wang C 2025 | SA vs EOI | -1.4214077 | -1.924127 | -0.9186882 | 1 |
| Wang C 2025 | SA vs ITPB | -1.7813845 | -2.201321 | -1.361448 | 1 |

| | | | | | |
|---|---|---|---|---|---|
| Wang C 2025 | SA vs TAP | -0.78142054 | -0.9719933 | -0.5908478 | 1 |
| Wang C 2025 | SA vs TEAS-TAP | -0.94685116 | -1.212322 | -0.6813803 | 1 |
| Fang Tang 2024 | SA vs EA | -0.6066012 | -0.8248634 | -0.388339 | 1 |
| Fang Tang 2024 | SA vs EspB | -0.84706375 | -1.24368 | -0.450448 | 1 |
| Fang Tang 2024 | SA vs ITPB | -1.8068143 | -2.225698 | -1.387931 | 1 |
| Fang Tang 2024 | SA vs TAP | -0.806851 | -0.9950913 | -0.6186107 | 1 |
| Fang Tang 2024 | SA vs TEAS-TAP | -0.96048991 | -1.225482 | -0.6954982 | 1 |
| Ruyi Xing 2022 | SA vs EA | -0.62394944 | -0.8515813 | -0.3963175 | 1 |
| Ruyi Xing 2022 | SA vs EOI | -1.4917613 | -2.020778 | -0.9627448 | 1 |
| Ruyi Xing 2022 | SA vs EspB | -0.85380798 | -1.251219 | -0.456397 | 1 |
| Ruyi Xing 2022 | SA vs ITPB | -1.851737 | -2.302822 | -1.400652 | 1 |
| Ruyi Xing 2022 | SA vs TAP | -0.85177494 | -1.103668 | -0.5998819 | 1 |

**Table S26** Sensitivity Analysis Using $CD4^{+}/CD8^{+}$ T-cell ratio as the Outcome Measure

| dropped_id | comparison | eff | lci | uci | connected |
|---|---|---|---|---|---|
| Liping Wang 2019 | SA vs EA | 0.19880129 | 0.145555 | 0.2520476 | 1 |
| Liping Wang 2019 | SA vs QLB | 0.18300009 | 0.0747193 | 0.2912808 | 1 |
| Liping Wang 2019 | SA vs TAP | 0.02000004 | -0.2484704 | 0.2884704 | 1 |
| Liping Wang 2019 | SA vs TEAS-TAP | 0.29000002 | 0.1617838 | 0.4182162 | 1 |
| Li Xin 2019 | SA vs EA | 0.26416069 | 0.2023154 | 0.326006 | 1 |
| Li Xin 2019 | SA vs QLB | 0.18300009 | 0.0747193 | 0.2912808 | 1 |
| Li Xin 2019 | SA vs TAP | 0.02000004 | -0.2484704 | 0.2884704 | 1 |
| Li Xin 2019 | SA vs TEAS-TAP | 0.29000002 | 0.1617838 | 0.4182162 | 1 |
| Min Zhou 2021 | SA vs EA | 0.22695545 | 0.1739027 | 0.2800082 | 1 |
| Min Zhou 2021 | SA vs QLB | 0.18300009 | 0.0747193 | 0.2912808 | 1 |
| Min Zhou 2021 | SA vs TAP | 0.02000004 | -0.2484704 | 0.2884704 | 1 |
| Min Zhou 2021 | SA vs TEAS-TAP | 0.29000002 | 0.1617838 | 0.4182162 | 1 |
| Fuchun Wang 2025 | SA vs EA | 0.19898801 | 0.1500641 | 0.2479119 | 1 |
| Fuchun Wang 2025 | SA vs TAP | 0.02000004 | -0.2484704 | 0.2884704 | 1 |

| | | | | | |
|---|---|---|---|---|---|
| Fuchun Wang 2025 | SA vs TEAS-TAP | 0.29000002 | 0.1617838 | 0.4182162 | 1 |
| T Hashimoto 1995 | SA vs EA | 0.10420674 | 0.044859 | 0.1635545 | 1 |
| T Hashimoto 1995 | SA vs QLB | 0.18300009 | 0.0747193 | 0.2912808 | 1 |
| T Hashimoto 1995 | SA vs TAP | 0.02000004 | -0.2484704 | 0.2884704 | 1 |
| T Hashimoto 1995 | SA vs TEAS-TAP | 0.29000002 | 0.1617838 | 0.4182162 | 1 |
| Ruyi Xing 2022 | SA vs EA | 0.19898801 | 0.1500641 | 0.2479119 | 1 |
| Ruyi Xing 2022 | SA vs QLB | 0.18300009 | 0.0747193 | 0.2912808 | 1 |

**Table S27** Sensitivity Analysis Using Postoperative opioid consumption as the Outcome Measure

| dropped_id | comparison | eff | lci | uci | connected |
|---|---|---|---|---|---|
| Ruizhu Liu 2019 | disconnected | | | | 0 |
| Yiquan Wu 2013 | SA vs EOI | -1.946827 | -4.283957 | 0.3903027 | 1 |
| Yiquan Wu 2013 | SA vs EspB | -0.18814623 | -2.091217 | 1.714925 | 1 |
| Yiquan Wu 2013 | SA vs QLB | -1.3032113 | -3.229721 | 0.6232983 | 1 |
| Yiquan Wu 2013 | SA vs TAP | -1.3391559 | -2.695898 | 0.0175862 | 1 |
| Fuchun Wang 2025 | SA vs EA | -2.2569528 | -3.529005 | -0.9849009 | 1 |
| Fuchun Wang 2025 | SA vs EOI | -1.8643533 | -3.429881 | -0.298826 | 1 |
| Fuchun Wang 2025 | SA vs EspB | -0.18814623 | -1.538185 | 1.161892 | 1 |
| Fuchun Wang 2025 | SA vs TAP | -1.2562783 | -2.04771 | -0.4648469 | 1 |
| Chen Q 2025 | disconnected | | | | 0 |
| Fang Tang 2024 | SA vs EA | -2.2570885 | -3.529373 | -0.984804 | 1 |
| Fang Tang 2024 | SA vs EspB | -0.18814623 | -1.538433 | 1.16214 | 1 |
| Fang Tang 2024 | SA vs QLB | -1.3032113 | -2.686337 | 0.0799138 | 1 |
| Fang Tang 2024 | SA vs TAP | -1.256538 | -2.048195 | -0.4648808 | 1 |
| Jeong H 2022 | SA vs EA | -2.2569528 | -3.529005 | -0.9849009 | 1 |
| Jeong H 2022 | SA vs EOI | -1.8643533 | -3.429881 | -0.298826 | 1 |
| Jeong H 2022 | SA vs QLB | -1.3032113 | -2.686094 | 0.0796714 | 1 |
| Jeong H 2022 | SA vs TAP | -1.2562783 | -2.04771 | -0.4648469 | 1 |

**Table S28** Meta-Regression Analysis of Short-term VAS pain scores at rest Using Time Point (1 hour) as a Moderator

| Intervention | Covariate | Coefficient | Standard Error | Z-statistic | P>z | lower confidence interval | upper confidence interval |
|---|---|---|---|---|---|---|---|
| EA vs EOI | Constant | -1.187794 | 0.6990035 | -1.7 | 0.089 | -2.557815 | 0.182228 |
| EA vs EspB | Constant | -0.3698681 | 0.511296 | -0.72 | 0.469 | -1.37199 | 0.6322537 |
| EA vs ITPB | Constant | -0.4000002 | 26.88729 | -0.01 | 0.988 | -53.09812 | 52.29812 |
| EA vs SA | 1 hour | -2.523594 | 0.7315079 | -3.45 | 0.876 | -3.957323 | -1.089865 |
| | Constant | 2.8 | 0.6658489 | 4.21 | 0 | 1.49496 | 4.10504 |
| EA vs SAPB | Constant | -1.323103 | 0.6130795 | -2.16 | 0.031 | -2.524717 | -0.1214896 |
| EA vs TAP | 1 hour | -1.428035 | 26.89062 | -0.05 | 0.958 | -54.13268 | 51.27661 |
| | Constant | 0.3999999 | 26.88739 | 0.01 | 0.988 | -52.29832 | 53.09832 |

**Table S29** Meta-Regression Analysis of Short-term VAS pain scores at rest Using Mean Age as a Moderator

| Intervention | Covariate | Coefficient | Standard Error | Z-statistic | P>z | lower confidence interval | upper confidence interval |
|---|---|---|---|---|---|---|---|
| EA vs EOI | Constant | 0.0783315 | 1.417707 | 0.06 | 0.956 | -2.700323 | 2.856986 |
| EA vs EspB | Constant | -0.2252457 | 0.9488058 | -0.24 | 0.812 | -2.084871 | 1.634379 |
| EA vs ITPB | Constant | -4.894082 | 4.766184 | -1.03 | 0.304 | -14.23563 | 4.447467 |
| EA vs SA | Mean age | -0.1216284 | 0.0927884 | -1.31 | 0.19 | -0.3034902 | 0.0602335 |
| | Constant | 8.731827 | 6.019177 | 1.45 | 0.147 | -3.065542 | 20.5292 |
| EA vs SAPB | Constant | 0.5593505 | 1.51294 | 0.37 | 0.712 | -2.405957 | 3.524658 |
| EA vs TAP | Mean age | -0.7166824 | 0.8489397 | -0.84 | 0.399 | -2.380574 | 0.9472089 |
| | Constant | 42.95272 | 51.15205 | 0.84 | 0.401 | -57.30346 | 143.2089 |

**Table S30** Meta-Regression Analysis of Short-term VAS Pain Scores at Rest Using Analgesic Technique (Single-shot vs Continuous Techniques) as a Moderator

| Intervention | Covariate | Coefficient | Standard Error | Z-statistic | P>z | lower confidence interval | upper confidence interval |
|---|---|---|---|---|---|---|---|
| EA vs EOI | Constant | 0.077958 | 18.92942 | 0 | 0.997 | -37.02302 | 37.17893 |
| EA vs EspB | Constant | -0.0008326 | 1.165701 | 0 | 0.999 | -2.285565 | 2.2839 |
| EA vs ITPB | Constant | -0.5617379 | 18.92967 | -0.03 | 0.976 | -37.66321 | 36.53974 |
| EA vs SA | Analgesic Technique | -0.2305841 | 1.327799 | -0.17 | 0.862 | -2.833022 | 2.371854 |
| | Constant | 1.228927 | 2.402672 | 0.51 | 0.609 | -3.480223 | 5.938077 |
| EA vs SAPB | Constant | -0.6024841 | 1.628584 | -0.37 | 0.711 | -3.79445 | 2.589482 |
| EA vs TAP | Analgesic Technique | -0.887363 | 18.93009 | -0.05 | 0.963 | -37.98966 | 36.21494 |
| | Constant | 1.125473 | 37.83241 | 0.03 | 0.976 | -73.02469 | 75.27563 |

**Table S31** Meta-Regression Analysis of Short-term VAS Pain Scores at Rest Using Surgical Approach (Laparoscopic vs Open Surgery) as a Moderator

| Intervention | Covariate | Coefficient | Standard Error | Z-statistic | P>z | lower confidence interval | upper confidence interval |
|---|---|---|---|---|---|---|---|
| EA vs EOI | Constant | 0.077958 | 18.92942 | 0 | 0.997 | -37.02302 | 37.17893 |
| EA vs EspB | Constant | -0.0008326 | 1.165701 | 0 | 0.999 | -2.285565 | 2.2839 |
| EA vs ITPB | Constant | -0.5617379 | 18.92967 | -0.03 | 0.976 | -37.66321 | 36.53974 |
| EA vs SA | Surgical Approach | -0.2305841 | 1.327799 | -0.17 | 0.862 | -2.833022 | 2.371854 |

| | | | | | | | |
|---|---|---|---|---|---|---|---|
| | Constant | 1.228927 | 2.402672 | 0.51 | 0.609 | -3.480223 | 5.938077 |
| EA vs SAPB | Constant | -0.6024841 | 1.628584 | -0.37 | 0.711 | -3.79445 | 2.589482 |
| EA vs TAP | Surgical Approach | -0.887363 | 18.93009 | -0.05 | 0.963 | -37.98966 | 36.21494 |
| | Constant | 1.125473 | 37.83241 | 0.03 | 0.976 | -73.02469 | 75.27563 |

**Table S32** Meta-Regression Analysis of Long-term VAS pain scores at rest Using Mean Age as a Moderator

| Intervention | Covariate | Coefficient | Std. err. | z | P>z | [95% conf. | interval] |
|---|---|---|---|---|---|---|---|
| EA vs EOI | Constant | -1.134695 | 0.7465253 | -1.52 | 0.129 | -2.597858 | 0.3284673 |
| EA vs EspB | Constant | 0.193515 | 0.5704512 | 0.34 | 0.734 | -0.9245488 | 1.311579 |
| EA vs ITPB | Constant | -1.093523 | 0.7550813 | -1.45 | 0.148 | -2.573455 | 0.386409 |
| EA vs SA | Mean age | 0.0132988 | 0.0498356 | 0.27 | 0.79 | -0.0843772 | 0.1109748 |
| | Constant | -0.024508 | 3.178313 | -0.01 | 0.994 | -6.253886 | 6.20487 |
| EA vs SAPB | Constant | 0.8929433 | 0.8011 | 1.11 | 0.265 | -0.6771839 | 2.46307 |
| EA vs TAP | Mean age | 0.1159434 | 0.0867579 | 1.34 | 0.181 | -0.0540989 | 0.2859858 |
| | Constant | -7.605439 | 5.437948 | -1.4 | 0.162 | -18.26362 | 3.052743 |
| EA vs TEAS-TAP | Constant | 0.237739 | 0.7078839 | 0.34 | 0.737 | -1.149688 | 1.625166 |

**Table S33** Meta-Regression Analysis of Long-term VAS pain scores at Rest Using Analgesic Technique (Single-shot vs Continuous Techniques) as a Moderator

| Intervention | Covariate | Coefficient | Std. err. | z | P>z | [95% conf. | interval] |
|---|---|---|---|---|---|---|---|
| EA vs EOI | Constant | -1.178565 | 0.5234156 | -2.25 | 0.024 | -2.204441 | -0.1526892 |
| EA vs EspB | Constant | 0.2737485 | 0.3725151 | 0.73 | 0.462 | -0.4563676 | 1.003865 |
| EA vs ITPB | Constant | -1.838574 | 0.5276738 | -3.48 | 0 | -2.872795 | -0.8043522 |

| | | | | | | | |
|---|---|---|---|---|---|---|---|
| EA vs SA | Analgesic Technique | 1.011086 | 0.4307605 | 2.35 | 0.019 | 0.1668109 | 1.855361 |
| | Constant | -1.028088 | 0.7910868 | -1.3 | 0.194 | -2.57859 | 0.5224139 |
| EA vs SAPB | Constant | 0.1829731 | 0.515163 | 0.36 | 0.722 | -0.8267278 | 1.192674 |
| EA vs TAP | Analgesic Technique | -0.3670687 | 0.5420243 | -0.68 | 0.498 | -1.429417 | 0.6952794 |
| | Constant | -0.371701 | 0.8553076 | -0.43 | 0.664 | -2.048073 | 1.304671 |
| EA vs TEAS-TAP | Constant | -0.8141123 | 0.4733864 | -1.72 | 0.085 | -1.741933 | 0.113708 |

**Table S34** Meta-Regression Analysis of Long-term VAS Pain Scores at Rest Using Surgical Approach (Laparoscopic vs Open Surgery) as a Moderator

| Intervention | Covariate | Coefficient | Std. err. | z | P>z | [95% conf. | interval] |
|---|---|---|---|---|---|---|---|
| EA vs EOI | Constant | -0.3702103 | 1.018015 | -0.36 | 0.716 | -2.365483 | 1.625062 |
| EA vs EspB | Constant | -0.0003801 | 0.6198636 | 0 | 1 | -1.21529 | 1.21453 |
| EA vs ITPB | Constant | -1.0301 | 1.020305 | -1.01 | 0.313 | -3.02986 | 0.9696601 |
| EA vs SA | Surgical Approach | 0.4051475 | 0.675282 | 0.6 | 0.549 | -0.918381 | 1.728676 |
| | Constant | 0.0941278 | 1.257472 | 0.07 | 0.94 | -2.370472 | 2.558728 |
| EA vs SAPB | Constant | 0.6987913 | 0.8360498 | 0.84 | 0.403 | -0.9398362 | 2.337419 |
| EA vs TAP | Surgical Approach | -0.6086286 | 0.9284072 | -0.66 | 0.512 | -2.428273 | 1.211016 |
| | Constant | 0.6783386 | 1.723862 | 0.39 | 0.694 | -2.700369 | 4.057047 |
| EA vs TEAS-TAP | Constant | -0.1505534 | 0.8373322 | -0.18 | 0.857 | -1.791694 | 1.490588 |

**Table S35** Meta-Regression Analysis of Short-term VAS pain scores during movement Using

Time Point (1 hour) as a Moderator

| Intervention | Covariate | Coefficient | Standard Error | Z-statistic | P>z | lower confidence interval | upper confidence interval |
|---|---|---|---|---|---|---|---|
| EA vs EOI | Constant | -0.7725961 | 0.3755319 | -2.06 | 0.04 | -1.508625 | -0.0365672 |
| EA vs EspB | Constant | -0.088641 | 0.189894 | -0.47 | 0.641 | -0.4608265 | 0.2835444 |
| EA vs ITPB | Constant | -0.1500001 | 34.78552 | 0 | 0.997 | -68.32836 | 68.02836 |
| EA vs SA | 1 hour | -3.269536 | 0.5763098 | -5.67 | 0.781 | -4.399082 | -2.13999 |
| | Constant | 4 | 0.5569213 | 7.18 | 0 | 2.908454 | 5.091545 |
| EA vs TAP | 1 hour | -1.022618 | 34.78679 | -0.03 | 0.977 | -69.20348 | 67.15824 |
| | Constant | 0.1499999 | 34.78572 | 0 | 0.997 | -68.02876 | 68.32876 |

**Table S36** Meta-Regression Analysis of Short-term VAS pain scores during movement Using Surgical Approach (Laparoscopic vs Open Surgery) as a Moderator

| Intervention | Covariate | Coefficient | Standard Error | Z-statistic | P>z | lower confidence interval | upper confidence interval |
|---|---|---|---|---|---|---|---|
| EA vs EOI | Constant | 0.1399982 | 23.33585 | 0.01 | 0.995 | -45.59743 | 45.87743 |
| EA vs EspB | Constant | 8.01e-16 | 0.1972459 | 0 | 1 | -0.3865949 | 0.3865949 |
| EA vs ITPB | Constant | -0.2599949 | 23.33585 | -0.01 | 0.991 | -45.99743 | 45.47744 |
| EA vs SA | Surgical Approach | -0.092609 | 0.2897714 | -0.32 | 0.749 | -0.6605504 | 0.4753324 |
| | Constant | 1.092609 | 0.4783641 | 2.28 | 0.022 | 0.1550325 | 2.030185 |
| EA vs TAP | Surgical Approach | -0.7519496 | 23.3369 | -0.03 | 0.974 | -46.49143 | 44.98753 |
| | Constant | 0.7919504 | 46.67104 | 0.02 | 0.986 | -90.68161 | 92.26551 |

**Table S37** Meta-Regression Analysis of Short-term VAS pain scores during movement Using Analgesic Technique (Single-shot vs Continuous Techniques) as a Moderator

| Intervention | Covariate | Coefficient | Standard Error | Z-statistic | P>z | lower confidence interval | upper confidence interval |
|---|---|---|---|---|---|---|---|
| EA vs EOI | Constant | -0.9769042 | 0.3951601 | -2.47 | 0.013 | -1.751404 | -0.2024046 |
| EA vs EspB | Constant | 0.1329084 | 0.1954543 | 0.68 | 0.497 | -0.250175 | 0.5159919 |
| EA vs ITPB | Constant | -1.376885 | 0.4101888 | -3.36 | 0.001 | -2.18084 | -0.5729297 |
| EA vs SA | Analgesic Technique | 0.8986762 | 0.2865269 | 3.14 | 0.002 | 0.3370937 | 1.460259 |
| | Constant | -0.3932097 | 0.4506011 | -0.87 | 0.383 | -1.276372 | 0.4899522 |
| EA vs TAP | Constant | -1.076932 | 0.2992554 | -3.6 | 0 | -1.663462 | -0.4904022 |

**Table S38** Meta-Regression Analysis of Short-term VAS pain scores during movement Using Mean Age as a Moderator

| Intervention | Covariate | Coefficient | Standard Error | Z-statistic | P>z | lower confidence interval | upper confidence interval |
|---|---|---|---|---|---|---|---|
| EA vs EOI | Constant | -0.3566247 | 0.4273622 | -0.83 | 0.404 | -1.194239 | 0.4809898 |
| EA vs EspB | Constant | -0.0788621 | 0.1967748 | -0.4 | 0.689 | -0.4645336 | 0.3068094 |
| EA vs ITPB | Constant | -1.880544 | 2.603596 | -0.72 | 0.47 | -6.983499 | 3.222411 |
| EA vs SA | Mean age | -0.0501269 | 0.0423315 | -1.18 | 0.236 | -0.1330951 | 0.0328413 |
| | Constant | 4.131231 | 2.690151 | 1.54 | 0.125 | -1.141367 | 9.403829 |
| EA vs TAP | Mean age | -0.1857809 | 0.4503714 | -0.41 | 0.68 | -1.068493 | 0.6969309 |
| | Constant | 10.6159 | 26.99795 | 0.39 | 0.694 | -42.2991 | 63.53091 |

**Table S39** Meta-Regression Analysis of Long-term VAS pain scores during movement Using Mean Age as a Moderator

| Intervention | Covariate | Coefficient | Standard Error | Z-statistic | P>z | lower confidence interval | upper confidence interval |
|---|---|---|---|---|---|---|---|
| EA vs EOI | Constant | -0.5114875 | 0.7885528 | -0.65 | 0.517 | -2.057023 | 1.034048 |
| EA vs EspB | Constant | -0.1490972 | 0.6007114 | -0.25 | 0.804 | -1.32647 | 1.028276 |
| EA vs ITPB | Constant | -1.069336 | 0.8221076 | -1.3 | 0.193 | -2.680637 | 0.5419655 |
| EA vs SA | Mean age | -0.0420003 | 0.0909097 | -0.46 | 0.644 | -0.22018 | 0.1361795 |
| | Constant | 3.537838 | 5.637947 | 0.63 | 0.53 | -7.512335 | 14.58801 |
| EA vs TAP | Mean age | -0.0327234 | 0.1166975 | -0.28 | 0.779 | -0.2614464 | 0.1959996 |
| | Constant | 2.078783 | 7.26737 | 0.29 | 0.775 | -12.165 | 16.32257 |
| EA vs TEAS-TAP | Constant | -0.3416261 | 0.9461826 | -0.36 | 0.718 | -2.19611 | 1.512858 |

**Table S40** Meta-Regression Analysis of Long-term VAS pain scores during movement Using Analgesic Technique (Single-shot vs Continuous Techniques) as a Moderator

| Intervention | Covariate | Coefficient | Standard Error | Z-statistic | P>z | lower confidence interval | upper confidence interval |
|---|---|---|---|---|---|---|---|
| EA vs EOI | Constant | -1.015285 | 0.2711901 | -3.74 | 0 | -1.546808 | -0.4837626 |
| EA vs EspB | Constant | 0.1082292 | 0.2333794 | 0.46 | 0.643 | -0.349186 | 0.5656444 |
| EA vs ITPB | Constant | -1.375269 | 0.2316494 | -5.94 | 0 | -1.829294 | -0.9212446 |
| EA vs SA | Analgesic Technique | 0.8160568 | 0.2427702 | 3.36 | 0.001 | 0.3402359 | 1.291878 |
| | Constant | -0.4550498 | 0.3348942 | -1.36 | 0.174 | -1.11143 | 0.2013307 |
| EA vs TAP | Constant | -0.3752937 | 0.1311916 | -2.86 | 0.004 | -0.6324245 | -0.1181628 |
| EA vs TEAS-TAP | Constant | -0.5616423 | 0.1751508 | -3.21 | 0.001 | -0.9049317 | -0.218353 |

**Table S41** Meta-Regression Analysis of Long-term VAS pain scores during movement Using

Surgical Approach (Laparoscopic vs Open Surgery) as a Moderator

| Intervention | Covariate | Coefficient | Standard Error | Z-statistic | P>z | lower confidence interval | upper confidence interval |
|---|---|---|---|---|---|---|---|
| EA vs EOI | Constant | -0.3900657 | 0.3804074 | -1.03 | 0.305 | -1.135651 | 0.3555192 |
| EA vs EspB | Constant | -0.0000377 | 0.2535635 | 0 | 1 | -0.4970129 | 0.4969376 |
| EA vs ITPB | Constant | -0.7500596 | 0.3533056 | -2.12 | 0.034 | -1.442526 | -0.0575934 |
| EA vs SA | Surgical Approach | -0.4698107 | 0.2904463 | -1.62 | 0.106 | -1.039075 | 0.0994535 |
| | Constant | 1.469749 | 0.5356053 | 2.74 | 0.006 | 0.419982 | 2.519516 |
| EA vs TAP | Surgical Approach | -0.527269 | 0.3264387 | -1.62 | 0.106 | -1.167077 | 0.112539 |
| | Constant | 0.7772024 | 0.6096704 | 1.27 | 0.202 | -0.4177297 | 1.972135 |
| EA vs TEAS-TAP | Constant | 0.0699283 | 0.2985441 | 0.23 | 0.815 | -0.5152073 | 0.6550639 |

**Table S42** Meta-Regression Analysis of Postoperative opioid consumption Using Mean Age as a Moderator

| Intervention | Covariate | Coefficient | Standard Error | Z-statistic | P>z | lower confidence interval | upper confidence interval |
|---|---|---|---|---|---|---|---|
| EA vs EOI | Constant | -0.2114226 | 4.284291 | -0.05 | 0.961 | -8.608479 | 8.185634 |
| EA vs EspB | Constant | 0.8927392 | 22.55675 | 0.04 | 0.968 | -43.31767 | 45.10315 |
| EA vs EOI | Constant | 0.1537296 | 16.05205 | 0.01 | 0.992 | -31.30772 | 31.61517 |
| EA vs SA | Mean age | 0.1747079 | 3.027699 | 0.06 | 0.954 | -5.759474 | 6.10889 |
| | Constant | -8.274669 | 184.6838 | -0.04 | 0.964 | -370.2483 | 353.699 |
| EA vs TAP | Mean age | 0.3663989 | 3.029269 | 0.12 | 0.904 | -5.57086 | 6.303658 |
| | Constant | -21.44034 | 184.7827 | -0.12 | 0.908 | -383.6078 | 340.7271 |

**Table S43** Meta-Regression Analysis of Postoperative opioid consumption Using Surgical

Approach (Laparoscopic vs Open Surgery) as a Moderator

| Intervention | Covariate | Coefficient | Standard Error | Z-statistic | P>z | lower confidence interval | upper confidence interval |
|---|---|---|---|---|---|---|---|
| EA vs EOI | Constant | -21.33396 | 130.0309 | -0.16 | 0.87 | -276.1898 | 233.5219 |
| EA vs EspB | Constant | -10.80978 | 77.17832 | -0.14 | 0.889 | -162.0765 | 140.4569 |
| EA vs EOI | Constant | -6.594268 | 129.7499 | -0.05 | 0.959 | -260.8994 | 247.7109 |
| EA vs SA | Surgical Approach | 42.18519 | 129.851 | 0.32 | 0.745 | -212.318 | 296.6884 |
| | Constant | -39.27952 | 259.5345 | -0.15 | 0.88 | -547.9579 | 469.3988 |
| EA vs TAP | Surgical Approach | 24.75422 | 129.8501 | 0.19 | 0.849 | -229.7473 | 279.2557 |
| | Constant | -25.24857 | 259.5388 | -0.1 | 0.923 | -533.9352 | 483.4381 |

**Table S44** Meta-Regression Analysis of Postoperative opioid consumption Using Morphine Consumption as a Moderator

| Intervention | Covariate | Coefficient | Standard Error | Z-statistic | P>z | lower confidence interval | upper confidence interval |
|---|---|---|---|---|---|---|---|
| EA vs EOI | Constant | -0.3042229 | 31.6342 | -0.01 | 0.992 | -62.30612 | 61.69767 |
| EA vs EspB | Constant | 0.1890653 | 22.37242 | 0.01 | 0.993 | -43.66007 | 44.03821 |
| EA vs QLB | Constant | -0.9254369 | 22.37264 | -0.04 | 0.967 | -44.77501 | 42.92413 |
| EA vs SA | Morphine analgesia use | 1.87829 | 22.37649 | 0.08 | 0.933 | -41.97883 | 45.73541 |
| | Constant | 0.377232 | 22.36711 | 0.02 | 0.987 | -43.46149 | 44.21596 |
| EA vs TAP | Morphine analgesia | 0.6954298 | 31.6407 | 0.02 | 0.982 | -61.3192 | 62.71006 |

| | | | | | | | |
|---|---|---|---|---|---|---|---|
| | use | | | | | | |
| | Constant | 0.3042227 | 31.6342 | 0.01 | 0.992 | -61.69768 | 62.30612 |

**Table S45** Meta-Regression Analysis of Postoperative opioid consumption Using Country as a Moderator

| Intervention | Covariate | Coefficient | Standard Error | Z-statistic | P>z | lower confidence interval | upper confidence interval |
|---|---|---|---|---|---|---|---|
| EA vs EOI | Constant | 0.3902352 | 0.9399754 | 0.42 | 0.678 | -1.452083 | 2.232553 |
| EA vs EspB | Constant | -0.0972922 | 31.62869 | 0 | 0.998 | -62.08838 | 61.8938 |
| EA vs QLB | Constant | 0.9514843 | 0.9568588 | 0.99 | 0.32 | -0.9239244 | 2.826893 |
| EA vs SA | Country | 2.163814 | 31.63528 | 0.07 | 0.945 | -59.84019 | 64.16782 |
| | Constant | 0.0908071 | 31.62865 | 0 | 0.998 | -61.90021 | 62.08182 |
| EA vs TAP | Constant | 0.9988452 | 0.6405669 | 1.56 | 0.119 | -0.2566428 | 2.254333 |

**Table S46** Meta-Regression Analysis of $CD4^+/CD8^+$ T-cell ratio Using Country as a Moderator

| Intervention | Covariate | Coefficient | Standard Error | Z-statistic | P>z | lower confidence interval | upper confidence interval |
|---|---|---|---|---|---|---|---|
| EA vs QLB | Constant | 0.0761267 | 0.0808181 | 0.94 | 0.346 | -0.0822738 | 0.2345272 |
| EA vs SA | Country | -0.2931259 | 0.0736498 | -3.98 | 0.876 | -0.4374768 | -0.1487749 |
| | Constant | 0.1862517 | 0.1015598 | 1.83 | 0.067 | -0.0128018 | 0.3853052 |
| EA vs TAP | Constant | -0.0868741 | 0.149137 | -0.58 | 0.56 | -0.3791772 | 0.205429 |
| EA vs TEAS-TAP | Constant | 0.1831259 | 0.0880856 | 2.08 | 0.038 | 0.0104812 | 0.3557705 |

**Table S47** Meta-Regression Analysis of $CD4^+/CD8^+$ T-cell ratio Using Analgesic Technique (Single-shot vs Continuous Techniques) as a Moderator

| Intervention | Covariate | Coefficient | Standard Error | Z-statistic | P>z | lower confidence interval | upper confidence interval |
|---|---|---|---|---|---|---|---|
| EA vs QLB | Constant | 0.1429356 | 0.2325258 | 0.61 | 0.539 | -0.3128066 | 0.5986778 |
| EA vs SA | Analgesic Technique | -0.1901874 | 0.1903629 | -1 | 0.318 | -0.5632918 | 0.182917 |
| | Constant | 0.1501401 | 0.3449749 | 0.44 | 0.663 | -0.5259982 | 0.8262785 |
| EA vs TAP | Constant | -0.0200524 | 0.2641871 | -0.08 | 0.939 | -0.5378495 | 0.4977447 |
| EA vs TEAS-TAP | Constant | 0.2499476 | 0.2351851 | 1.06 | 0.288 | -0.2110068 | 0.710902 |

**Table S48** Meta-Regression Analysis of Short-term VAS Pain Scores at Rest Using Surgical Approach (Laparoscopic vs Open Surgery) as a Moderator

| Intervention | Covariate | Coefficient | Standard Error | Z-statistic | P>z | lower confidence interval | upper confidence interval |
|---|---|---|---|---|---|---|---|
| EA vs QLB | Constant | 0.1429356 | 0.2325258 | 0.61 | 0.539 | -0.3128066 | 0.5986778 |
| EA vs SA | Surgical Approach | -0.1901874 | 0.1903629 | -1 | 0.318 | -0.5632918 | 0.182917 |
| | Constant | 0.1501401 | 0.3449749 | 0.44 | 0.663 | -0.5259982 | 0.8262785 |
| EA vs TAP | Constant | -0.0200524 | 0.2641871 | -0.08 | 0.939 | -0.5378495 | 0.4977447 |
| EA vs TEAS-TAP | Constant | 0.2499476 | 0.2351851 | 1.06 | 0.288 | -0.2110068 | 0.710902 |

**Table S49** Meta-Regression Analysis of $CD4^+/CD8^+$ T-cell ratio Using Mean Age as a Moderator

| Intervention | Covariate | Coefficient | Standard Error | Z-statistic | P>z | lower confidence interval | upper confidence interval |
|---|---|---|---|---|---|---|---|
| EA vs QLB | Constant | -0.1883006 | 0.1559532 | -1.21 | 0.227 | -0.4939631 | 0.117362 |

| Intervention | Covariate | Coefficient | Standard Error | Z-statistic | P>z | lower confidence interval | upper confidence interval |
|---|---|---|---|---|---|---|---|
| EA vs SA | Mean age | 0.0378475 | 0.0184502 | 2.05 | 0.06 | 0.0016857 | 0.0740093 |
|  | Constant | -2.47945 | 1.118779 | -2.22 | 0.027 | -4.672218 | -0.2866828 |
| EA vs TAP | Constant | 0.1690482 | 0.2426905 | 0.7 | 0.486 | -0.3066164 | 0.6447128 |
| EA vs TEAS-TAP | Constant | 0.4390482 | 0.2107508 | 2.08 | 0.037 | 0.0259841 | 0.8521122 |

**Table S50** Confidence ratings for each comparison for Short-term VAS pain scores at rest based on the CINeMA framework

| Comparison | Number of studies | Within-study bias | Reporting bias | Indirectness | Imprecision | Heterogeneity | Incoherence | Confidence rating |
|---|---|---|---|---|---|---|---|---|
| EA:EspB | 1 | No concerns | Low risk | No concerns | No concerns | Some concerns | No concerns | Moderate |
| EA:SA | 4 | No concerns | Low risk | No concerns | No concerns | Some concerns | No concerns | Moderate |
| EA:TAP | 1 | No concerns | Low risk | No concerns | No concerns | Some concerns | No concerns | Moderate |
| EOI:TAP | 1 | No concerns | Low risk | No concerns | No concerns | Some concerns | No concerns | Moderate |
| EspB:SA | 1 | No concerns | Low risk | No concerns | Some concerns | No concerns | No concerns | Moderate |
| ITPB:TAP | 1 | Some concerns | Low risk | No concerns | Some concerns | No concerns | No concerns | Low |
| SA:SAPB | 1 | No concerns | Low risk | No concerns | No concerns | Some concerns | No concerns | Moderate |
| SA:TAP | 3 | No concerns | Low risk | No concerns | No concerns | Some concerns | No concerns | Moderate |
| EA:EOI | 0 | No concerns | Low risk | No concerns | Some concerns | Some concerns | No concerns | Low |
| EA:ITPB | 0 | No concerns | Low risk | No concerns | Some concerns | No concerns | No concerns | Moderate |
| EA:SAPB | 0 | No concerns | Low risk | No concerns | Some concerns | No concerns | No concerns | Moderate |
| EOI:EspB | 0 | No concerns | Low risk | No concerns | Some concerns | Some concerns | No concerns | Low |
| EOI:ITPB | 0 | No concerns | Low risk | No concerns | Some concerns | Some concerns | No concerns | Low |

| | | | | | | | | |
|---|---|---|---|---|---|---|---|---|
| EOI:SA | 0 | No concerns | Low risk | No concerns | Some concerns | No concerns | No concerns | Moderate |
| EOI:SAPB | 0 | No concerns | Low risk | No concerns | Major concerns | No concerns | No concerns | Low |
| EspB:ITPB | 0 | No concerns | Low risk | No concerns | Some concerns | Some concerns | No concerns | Low |
| EspB:SAPB | 0 | No concerns | Low risk | No concerns | Some concerns | Some concerns | No concerns | Low |
| EspB:TAP | 0 | No concerns | Low risk | No concerns | No concerns | Some concerns | No concerns | Moderate |
| ITPB:SA | 0 | No concerns | Low risk | No concerns | No concerns | Some concerns | No concerns | Moderate |
| ITPB:SAPB | 0 | No concerns | Low risk | No concerns | Some concerns | Some concerns | No concerns | Low |
| SAPB:TAP | 0 | No concerns | Low risk | No concerns | No concerns | Major concerns | No concerns | Low |

**Table S51** Confidence ratings for each comparison for Long-term VAS pain scores at rest based on the CINeMA framework

| Comparison | Number of studies | Within-study bias | Reporting bias | Indirectness | Imprecision | Heterogeneity | Incoherence | Confidence rating |
|---|---|---|---|---|---|---|---|---|
| EA:EspB | 1 | No concerns | Low risk | No concerns | Some concerns | No concerns | No concerns | Moderate |
| EA:SA | 6 | No concerns | Low risk | No concerns | No concerns | No concerns | No concerns | High |
| EA:TAP | 1 | No concerns | Low risk | No concerns | Some concerns | Some concerns | No concerns | Low |
| EOI:TAP | 1 | No concerns | Low risk | No concerns | No concerns | No concerns | No concerns | High |

| | | | | | | | | |
|---|---|---|---|---|---|---|---|---|
| EspB:SA | 1 | No concerns | Low risk | No concerns | Some concerns | Some concerns | No concerns | Low |
| ITPB:TAP | 1 | Some concerns | Low risk | No concerns | Some concerns | No concerns | No concerns | Low |
| SA:SAPB | 1 | No concerns | Low risk | No concerns | No concerns | No concerns | No concerns | High |
| SA:TAP | 4 | No concerns | Low risk | No concerns | No concerns | Some concerns | No concerns | Moderate |
| SA:TEAS-TAP | 1 | Some concerns | Low risk | No concerns | Some concerns | No concerns | No concerns | Low |
| TAP:TEAS-TAP | 1 | Some concerns | Low risk | No concerns | No concerns | No concerns | No concerns | Moderate |
| EA:EOI | 0 | No concerns | Low risk | No concerns | No concerns | No concerns | No concerns | High |
| EA:ITPB | 0 | No concerns | Low risk | No concerns | Some concerns | Some concerns | No concerns | Low |
| EA:SAPB | 0 | No concerns | Low risk | No concerns | Some concerns | Some concerns | No concerns | Low |
| EA:TEAS-TAP | 0 | No concerns | Low risk | No concerns | Some concerns | No concerns | No concerns | Moderate |
| EOI:EspB | 0 | No concerns | Low risk | No concerns | Some concerns | No concerns | No concerns | Moderate |
| EOI:ITPB | 0 | No concerns | Low risk | No concerns | No concerns | No concerns | No concerns | High |
| EOI:SA | 0 | No concerns | Low risk | No concerns | Some concerns | Some concerns | No concerns | Low |
| EOI:SAPB | 0 | No concerns | Low risk | No concerns | No concerns | Some concerns | No concerns | Moderate |
| EOI:TEAS-TAP | 0 | No concerns | Low risk | No concerns | Some concerns | No concerns | No concerns | Moderate |
| EspB:ITPB | 0 | No concerns | Low risk | No concerns | Some concerns | Some concerns | No concerns | Low |
| EspB:SAPB | 0 | No concerns | Low risk | No concerns | No concerns | No concerns | No concerns | High |

| | | | | | | | | |
|---|---|---|---|---|---|---|---|---|
| EspB:TAP | 0 | No concerns | Low risk | No concerns | No concerns | No concerns | No concerns | High |
| EspB:TEAS-TAP | 0 | No concerns | Low risk | No concerns | No concerns | No concerns | No concerns | High |
| ITPB:SA | 0 | Some concerns | Low risk | No concerns | No concerns | Some concerns | No concerns | Low |
| ITPB:SAPB | 0 | No concerns | Low risk | No concerns | No concerns | Some concerns | No concerns | Moderate |
| ITPB:TEAS-TAP | 0 | No concerns | Low risk | No concerns | Some concerns | Some concerns | No concerns | Low |
| SAPB:TAP | 0 | No concerns | Low risk | No concerns | No concerns | Some concerns | No concerns | Moderate |
| SAPB:TEAS-TAP | 0 | No concerns | Low risk | No concerns | Some concerns | Some concerns | No concerns | Low |

**Table S52** Confidence ratings for each comparison for Short-term VAS pain scores during movement based on the CINeMA framework

| Comparison | Number of studies | Within-study bias | Reporting bias | Indirectness | Imprecision | Heterogeneity | Incoherence | Confidence rating |
|---|---|---|---|---|---|---|---|---|
| EA:EspB | 1 | No concerns | Low risk | No concerns | No concerns | Some concerns | No concerns | Moderate |
| EA:SA | 3 | No concerns | Low risk | No concerns | No concerns | Some concerns | No concerns | Moderate |
| EA:TAP | 1 | No concerns | Low risk | No concerns | No concerns | Some concerns | No concerns | Moderate |
| EOI:TAP | 1 | No concerns | Low risk | No concerns | Some concerns | No concerns | Some concerns | Low |
| EspB:SA | 1 | No concerns | Low risk | No concerns | Some concerns | No concerns | No concerns | Moderate |

| | | | | | | | | |
|---|---|---|---|---|---|---|---|---|
| ITPB:TAP | 1 | Some concerns | Low risk | No concerns | No concerns | No concerns | Some concerns | Low |
| SA:TAP | 2 | No concerns | Low risk | No concerns | No concerns | Major concerns | No concerns | Low |
| EA:EOI | 0 | No concerns | Low risk | No concerns | Some concerns | No concerns | Some concerns | Low |
| EA:ITPB | 0 | No concerns | Low risk | No concerns | Some concerns | No concerns | Some concerns | Low |
| EOI:EspB | 0 | No concerns | Low risk | No concerns | No concerns | No concerns | Some concerns | Moderate |
| EOI:ITPB | 0 | No concerns | Low risk | No concerns | No concerns | No concerns | Some concerns | Moderate |
| EOI:SA | 0 | No concerns | Low risk | No concerns | No concerns | No concerns | Some concerns | Moderate |
| EspB:ITPB | 0 | No concerns | Low risk | No concerns | Some concerns | No concerns | Some concerns | Low |
| EspB:TAP | 0 | No concerns | Low risk | No concerns | No concerns | Some concerns | No concerns | Moderate |
| ITPB:SA | 0 | No concerns | Low risk | No concerns | Some concerns | Some concerns | No concerns | Low |

**Table S53** Confidence ratings for each comparison for Long-term VAS pain scores during movement based on the CINeMA framework

| Comparison | Number of studies | Within-study bias | Reporting bias | Indirectness | Imprecision | Heterogeneity | Incoherence | Confidence rating |
|---|---|---|---|---|---|---|---|---|
| EA:EspB | 1 | No concerns | Low risk | No concerns | No concerns | No concerns | No concerns | High |
| EA:SA | 4 | No concerns | Low risk | No concerns | No concerns | Some concerns | No concerns | Moderate |
| EA:TAP | 1 | No concerns | Low risk | No concerns | No concerns | No concerns | No concerns | High |

| | | | | | | | | |
|---|---|---|---|---|---|---|---|---|
| EOI:TAP | 1 | No concerns | Low risk | No concerns | No concerns | Some concerns | No concerns | Moderate |
| EspB:SA | 1 | No concerns | Low risk | No concerns | No concerns | Some concerns | No concerns | Moderate |
| ITPB:TAP | 1 | Some concerns | Low risk | No concerns | No concerns | Some concerns | No concerns | Low |
| SA:TAP | 3 | No concerns | Low risk | No concerns | No concerns | No concerns | Some concerns | Moderate |
| SA:TEAS-TAP | 1 | Some concerns | Low risk | No concerns | No concerns | Some concerns | No concerns | Low |
| TAP:TEAS-TAP | 1 | Some concerns | Low risk | No concerns | No concerns | No concerns | No concerns | Moderate |
| EA:EOI | 0 | No concerns | Low risk | No concerns | No concerns | Some concerns | No concerns | Moderate |
| EA:ITPB | 0 | No concerns | Low risk | No concerns | No concerns | Some concerns | No concerns | Moderate |
| EA:TEAS-TAP | 0 | No concerns | Low risk | No concerns | No concerns | No concerns | No concerns | High |
| EOI:EspB | 0 | No concerns | Low risk | No concerns | No concerns | Some concerns | No concerns | Moderate |
| EOI:ITPB | 0 | No concerns | Low risk | No concerns | No concerns | Some concerns | No concerns | Moderate |
| EOI:SA | 0 | No concerns | Low risk | No concerns | No concerns | Some concerns | No concerns | Moderate |
| EOI:TEAS-TAP | 0 | No concerns | Low risk | No concerns | No concerns | Some concerns | No concerns | Moderate |
| EspB:ITPB | 0 | No concerns | Low risk | No concerns | Some concerns | No concerns | No concerns | Moderate |
| EspB:TAP | 0 | No concerns | Low risk | No concerns | No concerns | No concerns | No concerns | High |
| EspB:TEAS-TAP | 0 | No concerns | Low risk | No concerns | No concerns | No concerns | No concerns | High |
| ITPB:SA | 0 | Some concerns | Low risk | No concerns | No concerns | No concerns | No concerns | Moderate |

| | | | | | | | | |
|---|---|---|---|---|---|---|---|---|
| ITPB:TEAS-TAP | 0 | Some concerns | Low risk | No concerns | No concerns | Some concerns | No concerns | Low |

**Table S54** Confidence ratings for each comparison for Postoperative opioid consumption based on the CINeMA framework

| Comparison | Number of studies | Within-study bias | Reporting bias | Indirectness | Imprecision | Heterogeneity | Incoherence | Confidence rating |
|---|---|---|---|---|---|---|---|---|
| EA:SA | 1 | No concerns | Low risk | No concerns | No concerns | Some concerns | No concerns | Moderate |
| EA:TAP | 1 | No concerns | Low risk | No concerns | No concerns | Some concerns | No concerns | Moderate |
| EOI:TAP | 1 | No concerns | Low risk | No concerns | Some concerns | No concerns | No concerns | Moderate |
| ESPB:SA | 1 | Some concerns | Low risk | No concerns | Some concerns | No concerns | No concerns | Low |
| QLB:SA | 1 | No concerns | Low risk | No concerns | Some concerns | No concerns | No concerns | Moderate |
| SA:TAP | 3 | No concerns | Low risk | No concerns | No concerns | Some concerns | No concerns | Moderate |
| EA:EOI | 0 | No concerns | Low risk | No concerns | Some concerns | No concerns | No concerns | Moderate |
| EA:ESPB | 0 | No concerns | Low risk | No concerns | Some concerns | No concerns | No concerns | Moderate |
| EA:QLB | 0 | No concerns | Low risk | No concerns | Some concerns | Some concerns | No concerns | Low |
| EOI:ESPB | 0 | No concerns | Low risk | No concerns | Some concerns | No concerns | No concerns | Moderate |
| EOI:QLB | 0 | No concerns | Low risk | No concerns | Some concerns | No concerns | No concerns | Moderate |
| EOI:SA | 0 | No concerns | Low risk | No concerns | No concerns | Some concerns | No concerns | Moderate |

| | | | | | | | | |
|---|---|---|---|---|---|---|---|---|
| ESPB:QLB | 0 | No concerns | Low risk | No concerns | Some concerns | No concerns | No concerns | Moderate |
| ESPB:TAP | 0 | Some concerns | Low risk | No concerns | Some concerns | No concerns | No concerns | Low |
| QLB:TAP | 0 | No concerns | Low risk | No concerns | Some concerns | No concerns | No concerns | Moderate |

**Table S55** Confidence ratings for each comparison for $CD4^{+}$ /$CD8^{+}$ T-cell ratio based on the CINeMA framework

| Comparison | Number of studies | Within-study bias | Reporting bias | Indirectness | Imprecision | Heterogeneity | Incoherence | Confidence rating |
|---|---|---|---|---|---|---|---|---|
| EA:SA | 4 | No concerns | Low risk | No concerns | No concerns | Major concerns | No concerns | Low |
| QLB:SA | 1 | No concerns | Low risk | No concerns | Some concerns | Some concerns | No concerns | Low |
| SA:TAP | 1 | Some concerns | Low risk | No concerns | No concerns | No concerns | Some concerns | Low |
| SA:TEAS-TAP | 1 | No concerns | Low risk | No concerns | Some concerns | Some concerns | No concerns | Low |
| TAP:TEAS-TAP | 1 | No concerns | Low risk | No concerns | Some concerns | Some concerns | No concerns | Low |
| EA:QLB | 0 | No concerns | Low risk | No concerns | Some concerns | No concerns | Some concerns | Low |
| EA:TAP | 0 | Some concerns | Low risk | No concerns | Some concerns | No concerns | No concerns | Low |
| EA:TEAS-TAP | 0 | No concerns | Low risk | No concerns | Some concerns | No concerns | No concerns | Moderate |
| QLB:TAP | 0 | No concerns | Low risk | No concerns | Some concerns | No concerns | No concerns | Moderate |
| QLB:TEAS-TAP | 0 | No concerns | Low risk | No concerns | Some concerns | No concerns | No concerns | Moderate |

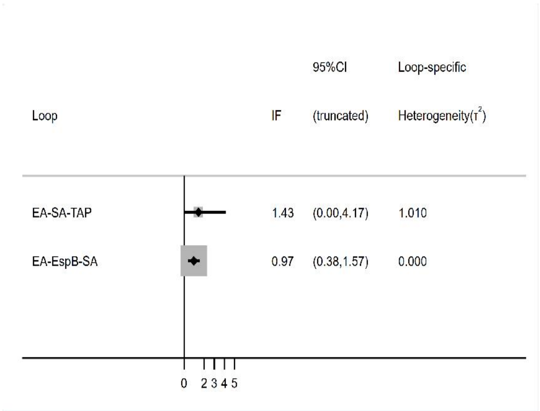


**Figure S1** Loop inconsistency analysis of Short-term VAS pain scores at rest

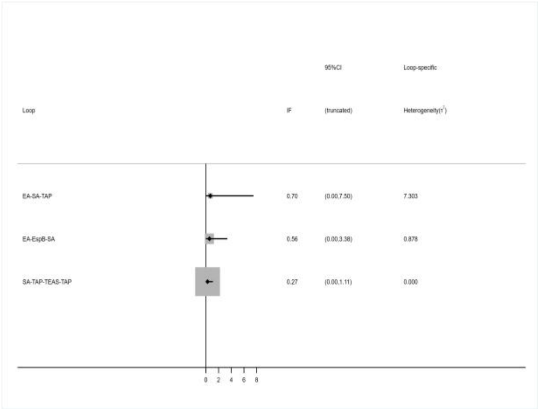


**Figure S2** Loop inconsistency analysis of Long-term VAS pain scores at rest

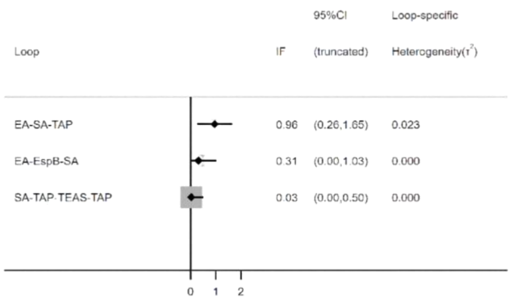


**Figure S3** Loop inconsistency analysis of Long-term VAS pain scores during movement

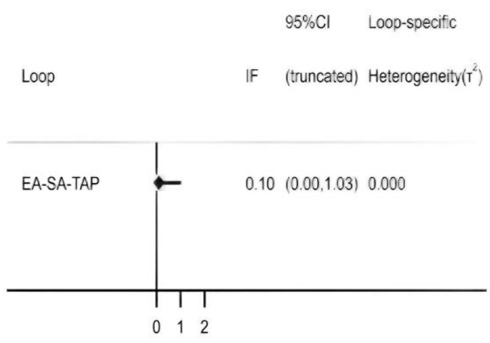


**Figure S4** Loop inconsistency analysis of Postoperative opioid consumption

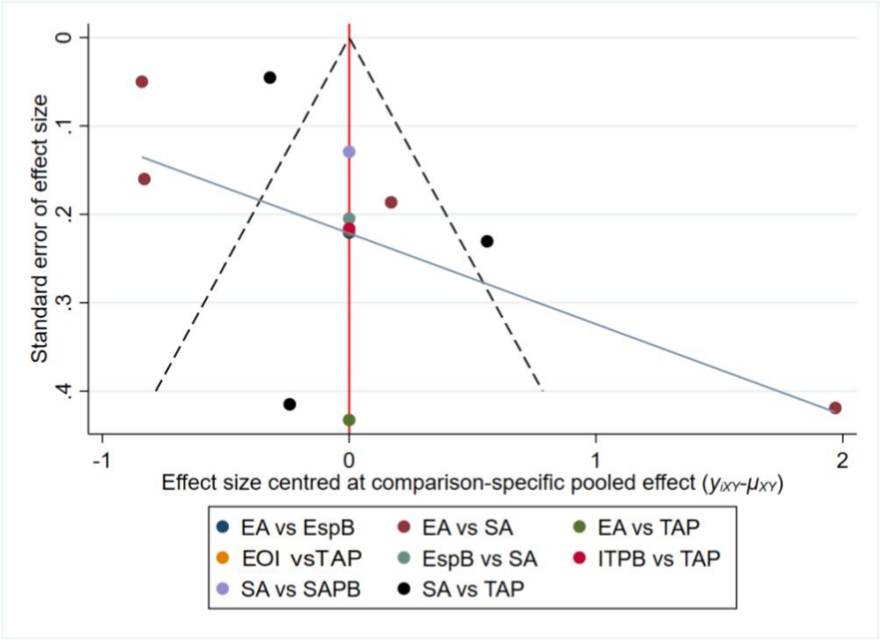


**Figure S5** The funnel plot of Short-term VAS pain scores at rest

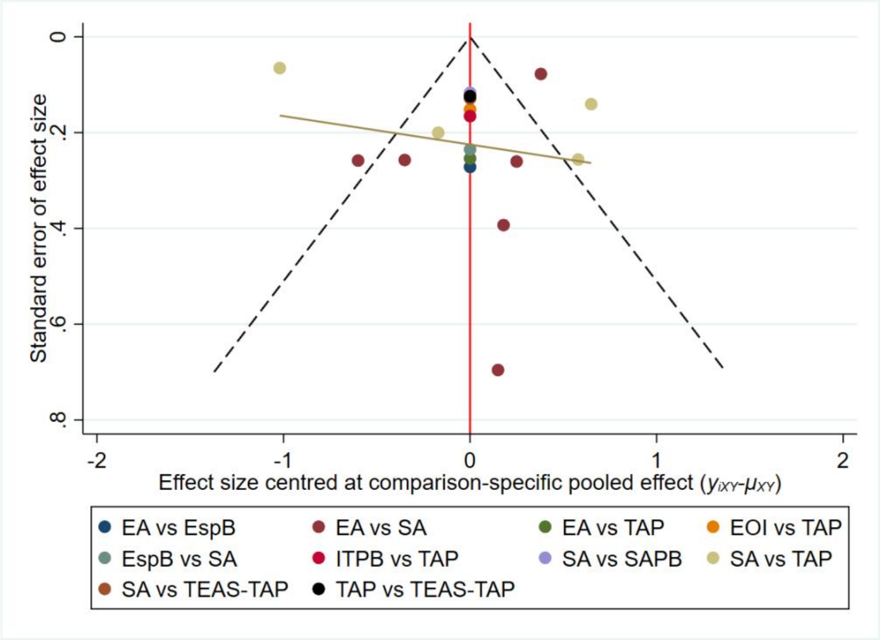


**Figure S6** The funnel plot of Long-term VAS pain scores at rest

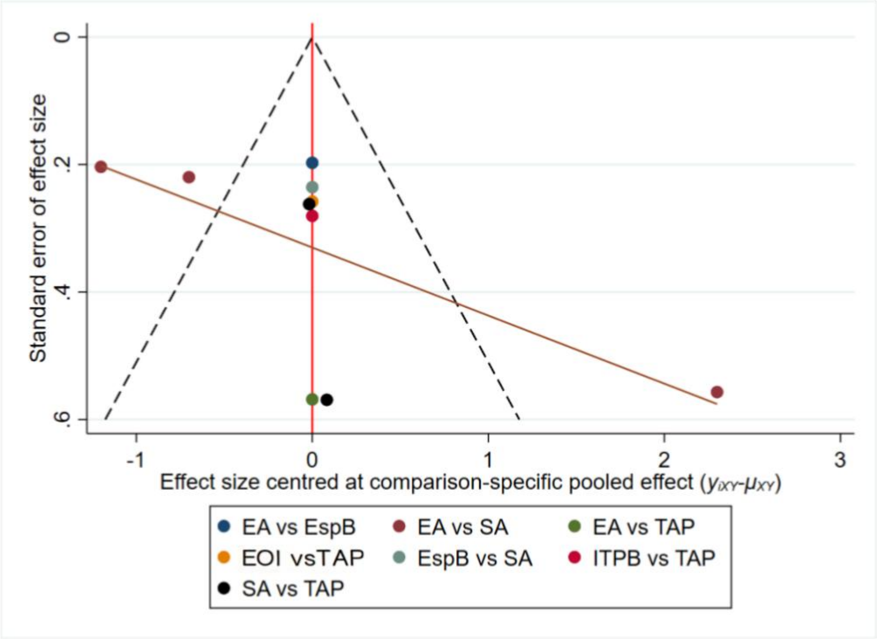


**Figure S7** The funnel plot of Short-term VAS pain scores during movement

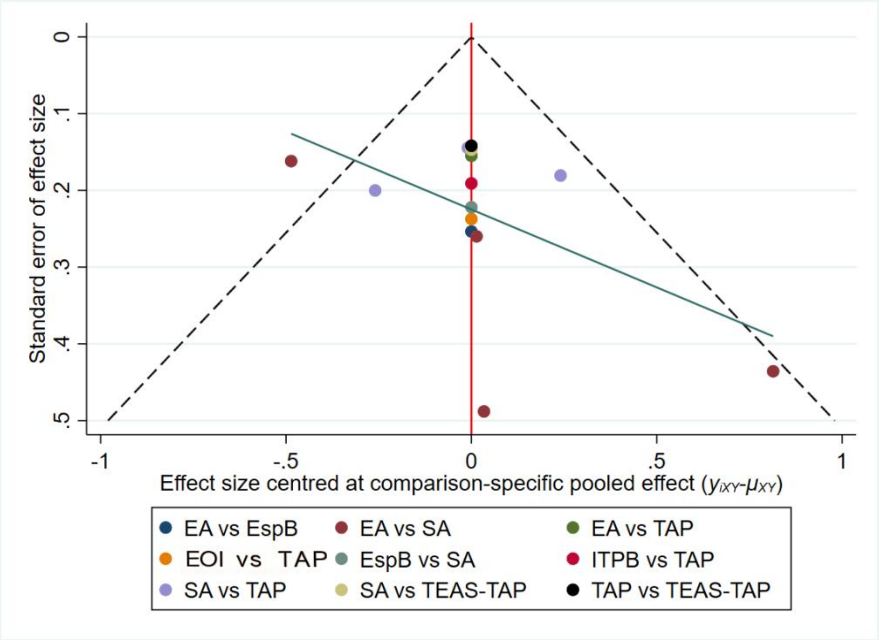


**Figure S8** The funnel plot of Long-term VAS pain scores during movement

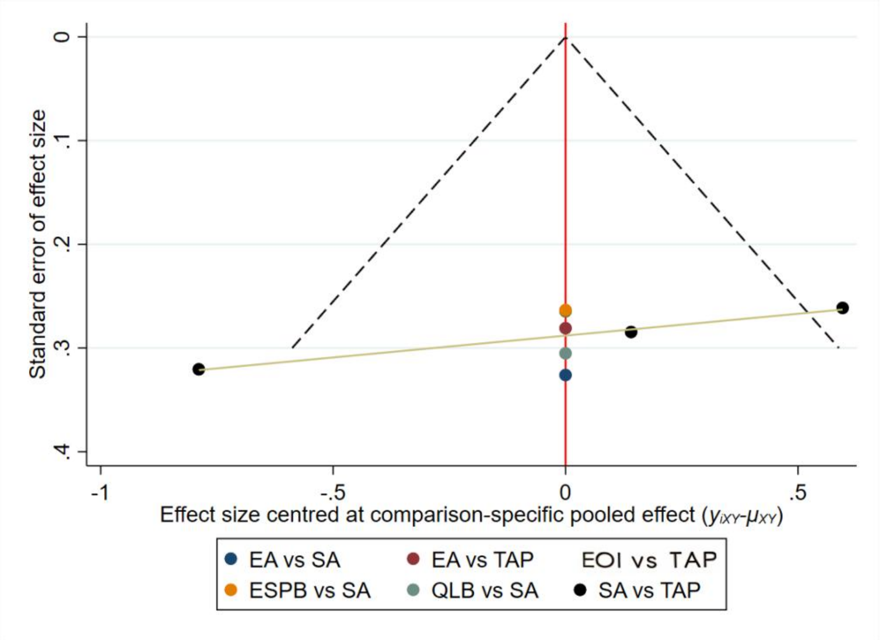


**Figure S9** The funnel plot of Postoperative opioid consumption

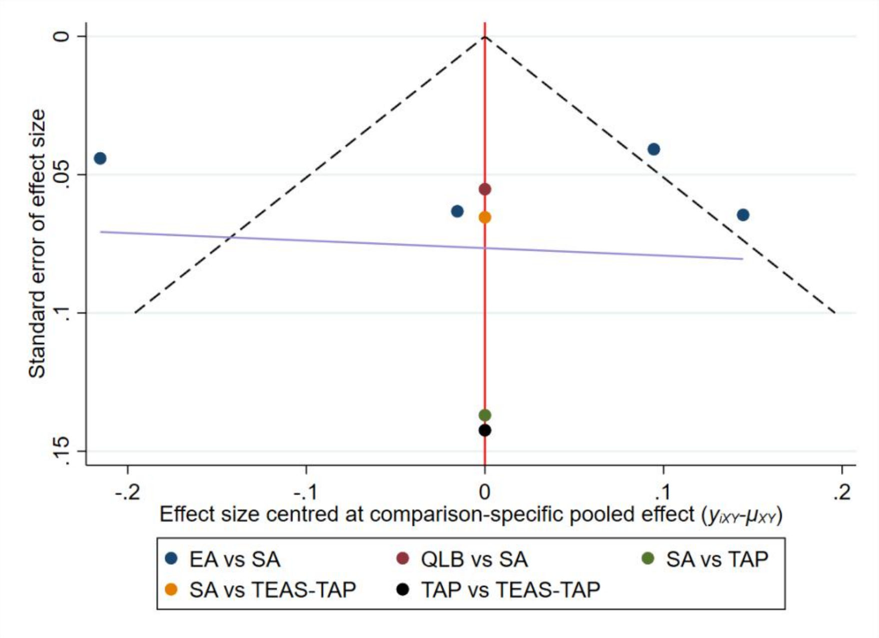


**FigureS10** The funnel plot of $CD4^{+}$ /$CD8^{+}$ T-cell ratio
